# Non-Targeted Analysis (NTA) of Plasma and Liver from Sprague Dawley Rats Exposed to Perfluorohexanesulfonamide (PFHxSA), a Precursor to Perfluorohexane Sulfonic Acid (PFHxS)

**DOI:** 10.3390/toxics13070523

**Published:** 2025-06-21

**Authors:** Denise K. MacMillan, Jackson G. Bounds, William A. Willis, Mark J. Strynar, Barbara A. Wetmore, Richard J. Liberatore, James P. McCord, Michael J. Devito

**Affiliations:** 1 Center for Computational Toxicology and Exposure, Office of Research and Development, U.S. Environmental Protection Agency (USEPA), Durham, NC 27709, USA; wetmore.barbara@epa.gov (B.A.W.); devito.michael@epa.gov (M.J.D.); 2 Oak Ridge Associated Universities (ORAU), Oak Ridge, TN 37830, USA; bounds.jackson@epa.gov (J.G.B.); willis.william@epa.gov (W.A.W.); 3 Center for Environmental Measurement and Modeling, Office of Research and Development, U.S. Environmental Protection Agency (USEPA), Durham, NC 27709, USA; strynar.mark@epa.gov (M.J.S.); liberatore.richard@epa.gov (R.J.L.); mccord.james@epa.gov (J.P.M.)

**Keywords:** PFAS, perfluoroalkylsulfonamide, PFASA, PFHxSA, PFHxS, non-targeted analysis (NTA), biotransformation

## Abstract

High-resolution accurate mass non-targeted analysis (NTA) is a useful discovery tool for metabolite characterization of in vivo dosing studies since it enables detection of both predicted and unexpected biotransformation products. We used NTA to investigate biotransformation of perfluorohexanesulfonamide (PFHxSA) in plasma and liver from male and female Sprague Dawley rats after a 5-day repeat exposure study. PFHxSA is an emerging per- and polyfluoroalkyl substance (PFAS) with unknown toxicity and a potentially reactive headgroup. NTA revealed the presence of predicted in vivo biotransformation products (BP) such as perfluorohexane sulfonic acid (PFHxS) and perfluorohexanesulfinic acid (PFHxSi). PFHxSi also has unknown toxicity and has not, to our knowledge, been previously reported as a PFHxSA BP in mammals. Multiple perfluoroalkyl ether sulfonamides, associated BPs, and novel PFAS were also detected in rat plasma and liver. We observed sex-specific distributions of the dosed compound and BPs, suggesting different toxicokinetics and biological responses. The presence of a complex mixture of predicted and unexpected PFAS in plasma and liver not only mimics the complexity of environmental exposure but also highlights the need for toxicity testing with mixtures and a more complete assessment of dosing solution purity.

## 1. Introduction

Non-targeted analysis (NTA) is a powerful analytical chemistry technique to probe media for analytes without prior anticipation of their presence or need for chemical standards. Because the acquisition is not solely focused on a predefined panel of chemicals, NTA can detect a substantially greater number of chemicals than targeted methods [1]. Both targeted and NTA techniques, however, are limited by implementation parameters such as extraction methods, ionization modes, and type of chromatography that define the chemical space amenable to detection [2,3]. The technique is used frequently for determination of an ever-growing number of per- and polyfluoroalkyl substances (PFAS) in the environment [4,5,6,7,8,9,10,11,12,13], food packaging materials [14,15], cosmetics [16], and aqueous film-forming foam (AFFF) for combating fires [17,18,19,20]. NTA has also proven useful for probing biological tissues to understand the metabolism of drugs and metabolic biotransformation products (BPs) [21,22,23]. Here, we applied NTA to plasma and liver from a five-day repeat dose study of male and female Sprague Dawley (SD) rats exposed to perfluorohexanesulfonamide (PFHxSA), an emerging PFAS compound with unknown toxicity. The dosing study was performed as part of a USEPA Transcriptomic Assessment Product (ETAP) [24,25], a new approach to address the lack of publicly available in vivo toxicity data for priority PFAS [26,27,28]. The PFHxSA ETAP is reported elsewhere [29]. The NTA component of the study was undertaken to inform chemical and biological disposition of the dosed compound with emphasis on potential biochemical reactivity of PFHxSA for the characterization of biotransformation products along with the occurrence of other xenobiotics. As biotransformation often creates products with increased water solubility, we selected liquid chromatography, an effective tool for the separation of water-soluble compounds in mixtures, as the method for the introduction of samples for mass spectrometric analysis.

PFHxSA is a perfluoroalkylsulfonamide (PFASA), a class of PFAS found in AFFF, water-resistant surface coatings, and heat-resistant fluids [30]. Their usefulness and widespread application led to detection of PFASA in the environment [31,32] and brought them to the attention of governmental agencies [27,33]. PFHxSA has also been detected in fish [34] and invertebrates [32] at sites that are contaminated with AFFF.

PFAS are often called “forever” chemicals because they are environmentally persistent as well as chemically inert. Biological transformations may occur for specific classes of PFAS, however [35]. PFASAs including PFHxSA and perfluorooctanesulfonamide (PFOSA) are known to undergo Phase I hydrolysis and N-dealkylation mediated by cytochrome P450 (CYP450) and/or other hepatic microsomal enzymes to highly toxic and persistent perfluorohexane sulfonic acid (PFHxS) and perfluorooctane sulfonic acid (PFOS), respectively [35,36,37,38]. Abiotic degradation of PFASA may preferentially generate perfluoroalkyl carboxylic acids [39]. Enzymatic hydrolysis is the major metabolic pathway for biotransformation [40] and is conserved across species including fish [35,41], rat [42,43,44], polar bear [45], earthworm [46], zebrafish embryo [40], and a selection of plants [47,48]. However, the transformation rate is noted to be very low because the high energy barrier for deamination is rate-limiting [43].

The Phase I hydrolysis reaction may occur through a perfluoroalkylsulfinic acid transient species [49,50]. The proposed intermediate has been observed in aerobic sludge where perfluorooctanesulfinic acid (PFOSi) was detected in the presence of PFOSA and PFOS [50]. A later study of biotransformation of N-ethyl-perfluorooctane sulfonic acid (N-EtFOSA) to PFOSA and onto PFOS in aerobic soil did not observe a sulfinic acid intermediate, however the absence was attributed to its increased reactivity [51]. More recently, perfluoropentane sulfinate (PFPeSi), PFHxSi, and PFOSi were detected in liver from fish exposed to AFFF-impacted groundwater [52]; all three sulfinates were also detected in the groundwater. The authors suggested that the sulfinate homologs may have resulted from biotransformation of PFASA by nitrifying bacteria or by the exposed fish. We are not aware of reports of PFASA biotransformation to PFOSi or PFHxSi in mammalian systems.

Phase I oxidation reactions may also occur with PFASA and other sulfonamides. N-hydroxylation by CYP450 was demonstrated for sulfonamide antibiotics [53]. For primary sulfonamides, N-hydroxylation proceeds via hydrogen abstraction from the amine to yield a nitrogen-centered radical [54,55]. Atmospheric abiotic hydroxylation of sulfonamides was shown to be a feasible pathway for the global distribution of perfluoroalkyl sulfonic acids [53]. The abstraction of hydrogen from the PFASA amine as a step in the formation of the sulfonic acid, however, requires higher energy than would be needed for removal from an N-methyl or N-ethyl side chain [53].

The biological metabolism of xenobiotic chemicals including PFAS through conjugation often leads to beneficial effects such as improved solubility that aids the elimination of potential hazards [56,57]. Fluorotelomer alcohols (FTOH), for example, become more soluble upon conjugation with cysteine, sulfate, or taurine [58]. Glucuronidation is another common Phase II conjugation reaction for PFASA [35,36] that leads to increased solubility. The formation of PFHxSA-N-glucuronide was demonstrated recently in mice exposed to AFFF [59]. The specific uridine diphospho-glucuronosyltransferases responsible for glucuronidation observed in rats and humans were identified from microsomes [43] and the reactions were shown to occur at a faster rate than the biotransformation to a sulfonic acid.

The presence of PFHxSA and other PFASAs at hundreds of industrial sites as well as in water and soil creates the potential for exposure and bioaccumulation in wildlife [60], increasing the possibility of human exposure [61], and potential health hazards. Biotransformation of PFHxSA to PFHxS is well known, but little data are available concerning the presence and relative abundance of other BPs, distribution in mammalian systems, toxicity, potential for bioaccumulation, and impacts of co-exposure.

## 2. Materials and Methods

### 2.1. In Vivo Study Design

The exposure study was performed under contract to Integrated Laboratory Systems (now Inotiv, Morrisville, NC, USA; Contract No.: 68HERC22D0010) as part of the USEPA Transcriptomic Assessment Product (ETAP) [24,25,29]. Animal handling and treatment procedures followed Animal Welfare Act Regulations 9 CFR 1A, 1–4 and the National Research Council Guide for the Care and Use of Laboratory Animals: Eighth Edition [62]. Reporting for this study is consistent with the ARRIVE guidelines [63,64]. Detailed descriptions of animal handling, procedures for dosing, clinical observations, termination, and collection of tissues were described previously [22,24,25,29]. Briefly, ninety-six male and female Sprague Dawley rats (*n* = 48/sex; 8–10 weeks old) purchased from Charles River Laboratory (Raleigh, NC, USA) were acclimated for ≥5 days, then dosed by oral gavage (5 mL/kg bodyweight; *n* = 5/sex, dose, and time point plus 8 vehicle controls per sex) once daily for five days with neat corn oil (Welch, Holme & Clark Co., Newark, NJ, USA; Lot#12-0768) vehicle or PFHxSA (Synquest Laboratories, Alachua, FL, USA; Lot No.: 797100) formulations. Prior to dose administration, RTI International (Durham, NC, USA; Contract No.: 68HERC21D0004) confirmed the purity of the PFHxSA standard by gas chromatography/mass spectrometry (GC/MS) on an Agilent (Santa Clara, CA, USA) 7890A GC coupled with an Agilent 5975C Mass Selective Detector (MSD). The PFHxSA purity was approximately 99.8% and consisted of 5 isomers with the primary isomer contributing 78.4% and the remaining 4 isomers contributing 21.4% to the total composition. One unknown impurity of 0.02% was also observed. Neither PFOS nor perfluorooctanoic acid (PFOA) were detected above the method detection limit of 5 ppm.

Animals were distributed to dose groups to minimize body weight differences as described in Mutlu et al. [29]. The minimum number of experimental animals and dose levels plus vehicle controls were used as required by ETAP Standard Methods [25]. At the time of study initiation, PFHxSA did not have any existing acute or repeat dose toxicity studies to assist in identifying the potential dose range in the study. The high dose was set at 100 mg/kg/day (mkd) based on the maximum solubility achieved in corn oil. The formulation concentrations decreased from 100 mkd at half log_10_ intervals except for the lowest dose level which was a full log_10_ interval below the next higher dose, for a total of 8 dose levels plus vehicle controls. The PFHxSA dosing formulations were prepared on Day 0 and used over the course of the study. Doses were administered to rats in randomized order and within ±1 h on each day of dosing. All rats survived until termination and were euthanized 24 h after the last dose. The study adhered to guidance regarding euthanasia prior to termination for humane reasons in accordance with the American Veterinary Medical Association (AVMA) Guidelines for the Euthanasia of Animals [65]. Tissue samples were collected immediately after termination. Blood was collected via cardiac puncture with tripotassium ethylene diamine tetraacetic acid (K_3_EDTA) for an anti-coagulant. Liver was collected and flash-frozen in liquid nitrogen. Frozen liver and plasma samples were transferred along with dosing solutions to the United States Environmental Protection Agency (USEPA), Office of Research and Development (ORD), Center for Computational Toxicology and Exposure (CCTE), Advanced Analytical Chemistry Methods Branch (AACMB), Durham, NC, USA. All samples were stored at ≤−70 °C prior to extraction.

### 2.2. Analytical Chemistry

#### 2.2.1. Sample Preparation

Plasma and liver from four dose groups (1, 10, 30, and 100 mkd) and the vehicle controls were selected for study by NTA for the identification of PFHxSA biotransformation products. The three dose groups below 1 mkd (0.01, 0.1, and 0.3 mkd) were not included in this study, as we anticipated only low concentrations of BPs would be present and likely would be undetectable by NTA. Preparation was performed as described in Bounds et al. [66] with modifications. Briefly, individual aliquots of plasma (25 µL) and liver (~10 mg) were thawed on ice and extracted as a batch with a mobile phase blank, a solvent blank, a process blank, and a method blank to monitor for the presence of laboratory contamination. Method blanks were prepared with commercial plasma (Sprague Dawley Rat Plasma K2EDTA Sex-Pooled Not Filtered; BioIVT, Westbury, NY, USA) and liver (Sprague Dawley Rat Liver Sex-Unspecified; BioIVT). A background signal of features in the blanks was multiplied by 3 and used to establish minimum detection limits for features in samples. A system suitability sample (SSS) was formed by pooling aliquots of plasma from each of the high dose samples from female rats. The SSS was analyzed at the start of each batch to assess instrument performance prior to study sample analysis. All study samples were extracted once due to limited sample volume and analyzed in randomized order individually, in triplicate. Liver samples were homogenized by using an Omni International (Kennesaw, GA, USA) Bead Ruptor 24. For both plasma and liver, proteins were precipitated with cold acidified acetonitrile (LC/MS-grade; Honeywell Burdick & Jackson, Charlotte, NC, USA). Supernatants were removed after centrifugation and stored at −20 °C until analysis. Immediately prior to analysis, aliquots were spiked with a mixture of isotopically labeled PFAS (MPFAC-24ES; Wellington Laboratories, Guelph ON, CA) that were used as tracers and retention time markers. Extracts were also diluted with an 80:20 water:methanol mixture containing 10 mM ammonium acetate (99%; Millipore Sigma; St. Louis, MO, USA). Water and methanol were both LC/MS-grade and obtained from Honeywell Burdick & Jackson.

The PFHxSA dosing solutions were extracted with Oasis Prime HLB solid-phase extraction cartridges (Waters, Milford, MA, USA) [66]. Extracts were handled in the same manner as the tissue extracts.

#### 2.2.2. Non-Targeted Analysis (NTA)

All plasma and liver sample extracts plus dosing solution extracts and an aliquot of PFHxSA standard (Synquest Laboratories, Lot No.: 797100) were analyzed by high-resolution accurate mass liquid chromatography/tandem mass spectrometry (LC/MS/MS) on a Sciex (Framingham, MA, USA) X500R QTOF mass spectrometer interfaced to a Shimadzu LC20 LC system. PFHxS (95% pure) was also purchased from SynQuest Laboratories. Chromatographic separation was achieved using a Phenomenex (Torrance, CA, USA) Kinetex EVO C18 column (100 mm × 2.1 mm, 2.6 µm particle size). The injection volume was 10 µL. Gradient elution was performed using 95:5 H_2_O:methanol (mobile phase A) and 95:5 methanol:H_2_O (mobile phase B), both containing 10 mM ammonium acetate. Gradient details are found in Supplementary Information (SI) Table S1. The mass spectrometer was operated in negative electrospray ionization (ESI) mode with both Information Dependent Analysis (IDA) and Sequential Window Acquisition of all Theoretical Mass Spectra (SWATH) scanning [67,68]. Mass calibration was verified to within 5 ppm before analysis and after every five injections. Samples were analyzed in triplicate in randomized order in a single analytical sequence. A web application, Random.org, was used to randomize the sample order. Analytical sequences started with the analysis of a mobile phase blank, a solvent blank, a process blank, and a method blank, followed by ~15 sample injections, after which the blanks were re-injected. The analytical sequence ended with analysis of the blanks. Instrument conditions are presented in SI Table S2.

A small subset of the plasma and dosing solution extracts were also analyzed by NTA in a different laboratory and by a different analyst for the confirmation of observed PFAS features. The confirmation analysis was performed on an Agilent 6546 QTOFMS coupled to a MOBILion Systems (Chadds Ford, PA, USA) MOBIE HRIM system operated in negative ESI mode. Sample components were chromatographically separated via a mobile phase gradient on an Agilent 1290 Infinity II LC system and a Waters Acquity UPLC BEH C18 1.7 µm, 2.1 mm × 50 mm column. The mobile phase gradient consisted of 2.5 mM ammonium acetate in water (%A) and 2.5 mM ammonium acetate in methanol (%B).

#### 2.2.3. NTA Data Analysis

The NTA Study Reporting Tool (SRT) was used in the preparation of this manuscript [69] (BP4NTA(2022): NTA Study Reporting Tool (PDF). doi:10.6084/m9.figshare.19763482 [PDF]).

A screening list of potential BPs was generated using all metabolite transition pathways available in the USEPA web application CTS: Chemical Transformation Simulator 1.0 [70] and Biotransformer 3.0 [71,72] prediction tools. In some instances, molecular structure predictions were performed using the open-source software tool MetFrag [73,74,75] and observed MS/MS fragment masses.

Workflows using Sciex OS 3.3, MarkerView 1.3.1, and the open-source mass spectrometry data-processing software mzMine 3 [76,77] were applied to the raw data. One plasma sample, r88, was excluded from data processing due to a preparation error. Data files processed with Sciex OS 3.3 were searched against the Sciex Fluorochemical library and an in-house spectral library created with 130 spectra from synthesized and commercial PFAS analyzed on the same instrument using the same IDA instrument acquisition conditions. Search parameters are given in SI Table S3. For use with mzMine 3, raw data files were first converted from the vendor format (.wiff) to .mzML files [78] by using the open-source file conversion tool MSConvert, included in the open-source package ProteoWizard [79]. Data handling procedures for both open-source software packages used parameters previously described in Whitehead et al. [13]. The raw data files for all samples at all dose levels were also processed together by matrix using Sciex MarkerView 1.3.1. Data-processing parameters are given in SI Table S4. Peak areas for features corresponding to the tracers were plotted for each sample using INTERPRET NTA [80]. See SI Table S5 for INTERPRET NTA data-processing parameters, and SI Figure S1A,B for tracer responses and other quality control results. Most peak areas were within 30% of the average peak area of the matrix blanks, with deviations above and below the average due to matrix suppression or the enhancement of ionization of some masses for high-dose tissue samples and the dosing solutions. Features occurring in the MS1 peak list for the 100 mkd dose plasma samples were filtered for CF_2_ Kendrick mass defect within the range, −0.11 to 0.12, that is expected for most probable PFAS. Significance testing (Student’s *t*-test) was applied for the 100 mkd dose compared to vehicle controls, and non-significant features (*p* > 0.05) were not considered further. The feature list was further reduced by removing isotopes and only selecting features with −log fold-change ≥ 0.01 compared to those for the same sex vehicle controls and features that appeared in at least 2 of the 3 replicate injections. The masses in the reduced feature list were assumed to be (M-H)^−^ ions, converted to the corresponding neutral species, and searched against the USEPA CompTox Chemicals Dashboard [81] list “PFASSTRUCTV5” (https://comptox.epa.gov/dashboard/chemical-lists/PFASSTRUCTV5 (accessed on 22 January 2025)). Results from the processing methods were assessed for consistency. Search results that included metals, halogens other than fluorine, and cyclic structures were not considered further. The remaining identifications were evaluated for plausible presence in the samples. Spectra for features not already identified by library matches were manually annotated and evaluated for similarity to spectra in the USEPA prototype tool Analytical Methods and Open Spectra (AMOS) database [13,82]. Identification confidence is reported using the Schymanski scale [83]. Features were background subtracted for relative quantitation.

#### 2.2.4. Glucuronide Formation Assay

An Evoluchem (HepatoChem, Beverley, MA, USA) Glucuronidation Screening Kit was used to investigate the potential for glucuronidation of PFHxSA. The assay was performed according to vendor instructions. The kit uses a proprietary glucuronidation reagent and solvents to yield a glucuronide adduct of the substrate based on the Koenigs–Knorr reaction [84]. Aliquots of PFHxSA (~5 mg) were dissolved in Solvents 1 and 2 provided in the kit. Brief sonication was required to dissolve PFHxSA in Solvent 1; however, PFHxSA dissolved readily in Solvent 2. An aliquot of the Solvent 1 mixture (50 µL) was added to each of the four vials (A1 through D1) containing one of four different salts (Ag_2_CO_3_, Ag_2_O, Cs_2_CO_3_, or Na_2_CO_3_), to generate four test conditions. Similarly, an aliquot of the Solvent 2 mixture (50 µL) was added to a second set of vials (E1 through H1) with the same salts to yield a second set of four test conditions. The reactions were allowed to proceed at room temperature overnight to form a protected intermediate that was then decoupled by addition of methanolic NaOH in vials A2 through H2. Post-reaction, aliquots (5 µL) were taken from each of the decoupling reaction vials and diluted with 85 µL of 50:50 water:acetonitrile and 10 µL of Wellington MPFAC-24ES tracers for NTA as described above for the analysis of tissue samples.

## 3. Results

### 3.1. Data Processing and Filtering

NTA data for plasma processed initially with Sciex MarkerView yielded a peak list with 5566 features. After filtering out early eluting features (*n* = 227), isotopes (*n* = 137), features outside the KMD range (*n* = 3049), and features not statistically different between 100 mkd dose and control (*n* = 1354) and with −log fold-change ≤ 0.01 (*n* = 560), only 239 features were retained for potential identification. The resulting list of 239 putative chemicals was assessed for concordance with the chemical space of the extraction technique, analytical method, and exposure study. Sciex OS processing was used to search spectral libraries for matching product ion spectra with matches discussed below. Product ion spectra for features of interest without library matches were manually annotated. NTA data for liver, dosing solutions, and a PFHxSA standard were processed in the same manner.

### 3.2. Dosing Solution and PFHxSA Standard NTA Results Summary

The NTA of the 20 mg/mL dosing solution and the ~500 ng/mL PFHxSA standard by LC/MS/MS detected several impurities not observed in the pre-administration GC/MS purity evaluation of the neat chemical. The qualitative NTA results for the dosing solution are presented in SI Table S7 along with results for plasma, liver, and the PFHxS standard. The most abundant impurity in the dosing solution and PFHxSA standard was PFHxS with peak abundances that were ~3% and ~6% of the PFHxSA signal, respectively. Note that the high concentrations of PFHxSA in the analyzed aliquots of dosing solution and standard likely led to ion signal suppression in the source of the mass spectrometer. This phenomenon would give the relative amounts of other ions a higher-than-actual value. Thus, the relative percentages of PFHxS and other compounds discussed here should be considered high estimates.

In addition to PFHxS, we identified a homologous series of perfluoroalkylsulfonamides with from four to eight carbons (C4 to C8) in the CF2 chains with level 2a confidence by matching to the Sciex Fluorochemical library or in-house PFAS library. By using NTA, we were also able to find features we tentatively identified as the C4 to C9 homologs of perfluoroalkyl ether sulfonamides in the dosing solution. A trace amount of a potential PFHxSA BP, perfluorohexanesulfinic acid, was also found in the dosing solution at ~0.003% of the PFHxSA signal. The identity of the feature was assigned based on the MS1 mass in the dosing solution and the corresponding product ion spectrum from plasma since the abundance of the feature was too low in the dosing solution to produce the spectrum. PFOA and PFOS were not detected in the dosing solution or the PFHxSA standard, in agreement with the GC/MS analysis. The peak areas for the sulfonamide impurities were less than 4% of the PFHxSA peak area in the standard, and peak areas for the other impurities, save PFHxS, were all less than 0.05%. Percentages tended to be slightly higher in the dosing solution, likely due to suppression of the higher abundance PFHxSA signal. A more-detailed discussion on the identification of these impurities is described the following sections.

### 3.3. Feature Annotation and Identification of Predicted Biotransformation Products PFHxS, PFHxSi, and PFHxSA-N-glucuronide

**Figure 1:**
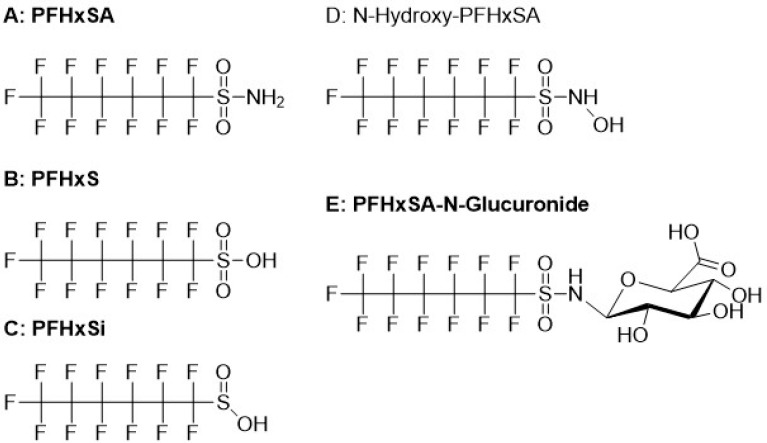
Chemical structures of PFHxSA and predicted biotransformation products. Bolded text indicates the product was observed. (**A**): Perfluorohexanesulfonamide (PFHxSA); (**B**): Perfluorohexane sulfonic acid (PFHxS); (**C**): Perfluorohexanesulfinic acid (PFHxSi); (**D**): N-Hydroxyperfluorohexanesulfonamide (N-Hydroxy-PFHxSA); (**E**): Perfluorohexane-N-glucuronide. See SI Table S6 for further information on biotransformation predictions.

Four BPs were predicted for PFHxSA under environmental, gut microbiome, and mammalian metabolism conditions: PFHxS, PFHxSA-N-glucuronide, PFHxSi, and N-hydroxy-PFHxSA (See SI Table S6). Chemical structures for the predicted BPs are shown in Figure 1. Using NTA, PFHxS, PFHxSA-N-glucuronide, and PFHxSi, as well as intact PFHxSA, were identified in dosed rat plasma and liver. See SI Figure S2 for annotated spectra of select features. See SI Table S7 for details of the identification of the PFHxSA BPs and other features of interest detected in the study samples and dosing solution. The observed features were confirmed to be present in the plasma and dosing solution samples by analysis on the Agilent 6546 QTOFMS.

PFHxSA and PFHxS were observed to be the major PFAS constituents of plasma and liver samples from dosed animals that were examined using NTA. For the feature identified as PFHxS, the mass of the MS1 molecular ion was within 5 ppm of the theoretical monoisotopic mass of the (M-H)^−^ ion of C_6_HF_13_O_3_S. The product ion spectrum gave a library match (Sciex Fluorochemical library) to PFHxS (fit = 100; reverse fit = 100). The spectrum included fragment ions annotated as SO_3_^−^ and FSO_3_^−^ that are characteristic fragments of perfluoroalkyl sulfonic acids. PFAS backbone fragments C_2_F_5_^−^, C_3_F_7_^−^, and C_5_F_9_^−^ were also observed. The retention time of the feature agreed with that of an authentic PFHxS standard. The dosed compound PFHxSA was also confirmed with a commercial standard, enabling identification of both compounds with level 1 confidence (matched against the analytical standard).

**Figure 2:**
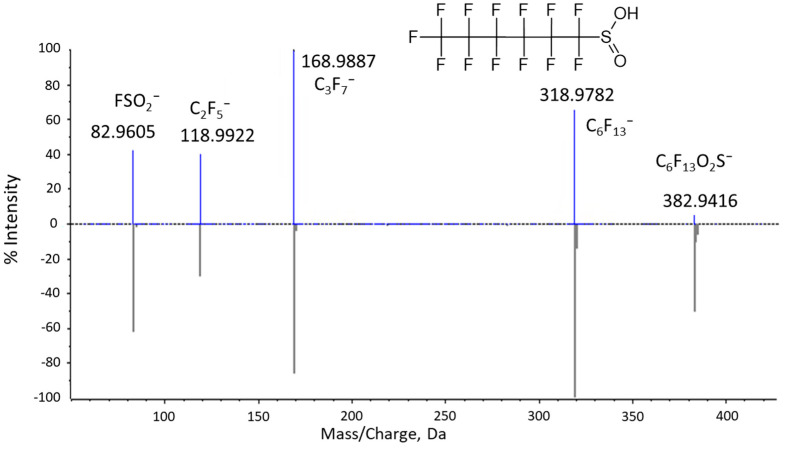
Product ion spectrum of the ion of *m*/*z* 382.9416 (top) detected in plasma mirrored with the perfluorohexanesulfinic acid spectrum from the Sciex Fluorochemical library (bottom).

A feature with an MS1 monoisotopic mass that fits to within 5 ppm of the theoretical mass of the (M-H)^−^ of the formula C_6_HF_13_SO_2_ for PFHxSi was observed in both plasma and liver from dosed animals. The feature was observed in the 20 mg/mL dosing solution and PFHxSA standard at 0.003% of the PFHxSA signal. The product ion spectrum from plasma matched to PFHxSi in the Sciex Fluorochemical library (fit = 91.5; reverse fit = 100). All fragment ions were representative of PFAS (Figure 2) including the FSO_2_^−^ ion which is characteristic of a perfluoroalkylsulfinic acid. A confidence level of 2a (library spectrum match) was assigned to identification of the feature, as a commercial standard was not available to provide level 1 confidence.

In addition to PFHxSi, a low abundance, later eluting feature with a mass ~100 Da higher was detected in plasma and liver. Based on the exact mass, we assigned the formula C_8_HF_17_SO_2_ to the feature. The feature was not detected in the dosing solution or the PFHxSA standard. The product ion spectrum included the FSO_2_^−^ ion and perfluoroalkyl fragment ions, consistent with a longer chain homolog of PFHxSi (SI Figure S2A2). The observed spectrum was consistent with the reference spectrum for perfluorooctanesulfinic acid (PFOSi) in AMOS [13], permitting level 2a confidence for the identification of the ion as PFOSi.

NTA also revealed the presence of three closely eluting MS1 features in plasma and liver with exact masses that were consistent with PFHxSA-N-glucuronide (SI Figure S3). The features eluted ahead of PFHxSA and PFHxS, indicating comparatively higher polarity. Synthesis of PFHxSA-N-glucuronide in our laboratory (Section 2.2.4) yielded a product that provided a similar chromatographic pattern, matching MS1 spectrum, and similar product ion spectra for all three features. The glucuronidated PFHxSA species was observed for all synthesis conditions, with the highest abundance occurring for condition F2. The product ion spectra from the synthesized compound included fragments annotated as C_2_HO_3_^−^, C_5_H_5_O_3_^−^, C_5_H_7_O_4_^−^, and C_6_H_7_O_6_^−^ that are indicative of a glucuronide moiety [36], the alkyl PFAS fragment C_6_F_13_^−^, the perfluorohexanesulfonyl backbone fragment C_6_HF_13_NO_2_S^−^, and the fragment C_8_H_3_F_13_NO_3_S^−^, which corresponds to the PFHxSA backbone with a portion of the glucuronide moiety. The product ion spectra from plasma matched the in-house PFAS library spectrum of synthesized PFHxSA-N-glucuronide (fit = 93.9; reverse fit = 100.0) and allowed the assignment of confidence level 1 to the identification. The PFHxSA-N-glucuronide was not found in the dosing solution.

NTA data from both plasma and liver were interrogated for the mass of the (M-H)^−^ ion of the predicted N-hydroxylation product having the formula C_6_H_2_F_13_NO_3_S (See SI Table S6), though the presence of the hydroxyl moiety on the headgroup (see Figure 1D) suggests the BP may be more amenable to gas chromatography/mass spectrometry (GC/MS). Surprisingly, a feature matching the theoretical mass for the N-hydroxy-PFHxSA anion (*m*/*z* 413.9475) was observed at low abundance in the highest dose plasma samples. However, the feature eluted after PFHxSA which suggested the feature was less polar than PFHxSA. Hydroxylation at the site of the amide nitrogen would lead to increased polarity. The product ion spectrum (SI Figure S2B3) included fragment ions annotated as SO_2_^−^ and NO_2_S^−^ as well as ions annotated as CF_3_O^−^, C_2_F_5_O^−^, C_3_F_7_O^−^, and C_4_F_9_O^−^ that suggest the presence of positional isomers of perfluoroalkyl ether sulfonamides. The spectrum did not match any spectra in the Sciex Fluorochemical library or the in-house PFAS library and the formula C_6_H_2_F_13_NO_3_S was not found in the USEPA CompTox Chemicals Dashboard [81]. The formula appeared in PubChem [85] but for compounds such as sulfamoyl fluorides and perfluorinated alcohols that would not generate the observed product ion spectrum or not be amenable to LC/MS/MS. A MetFrag [73,74,75] query using the product ion spectrum peak list did not suggest any plausible candidates. Based on annotations of the product ion spectrum and formula fit, we tentatively identified the feature as a co-eluting mixture of positional isomers of perfluorohexane ether sulfonamides with a six carbon C-F chain and the generic formula CF_3_(CF_2_)_x_-O-(CF_2_)_y_-SO_2_NH_2_, where the sum of x and y is 5.

### 3.4. Feature Annotation and Identification of Precursor Perfluoroalkyl Ether Sulfonamide Impurities and Perfluoroalkyl Ether Sulfonic Acid Biotransformation Products

The isomeric mixture of perfluorohexane ether sulfonamides with the formula C_6_H_2_F_13_NO_3_S discussed above was not an anticipated component of the three matrices (plasma, liver, and dosing solution) investigated here but demonstrated the value of the NTA approach. By using NTA, we were also able to find features that we tentatively identified as the 4 to 9 carbon (C4 to C9) homologs of the perfluorohexane ether sulfonamide species in the dosing solution. Product ion spectra for these features (SI Figure S2B) showed a mix of fragments indicative of the methoxy isomer and isomers with other positions along the carbon chain for the ether oxygen, and an unknown co-eluting PFAS. The product ion spectrum from the proposed C4 perfluoroalkyl ether sulfonamide homolog (SI Figure S2B1) included fragment ions annotated as CF_3_O^−^, CF_2_SO_2_N^−^, and C_2_F_4_SO_2_N⁻. The presence of the methoxy fragment and the two fragments originating from the headgroup suggests the presence of at least three C4 ether isomers: methoxy, ethoxy, and propoxy.

The product ion spectrum for the ion fitting the C5 perfluoroalkyl ether sulfonamide formula was only observed in the dosing solution. The spectrum included some representative fragments of polyfluoroalkyl ether sulfonamides (NSO_2_^−^, CF_3_O^−^, C_2_F_5_O^−^, and C_4_F_8_SO_2_N^−^) (See SI Figure S2B2). However, the spectrum also included fragments annotated as multiple HF losses from the molecular ion and an unidentified fragment pair separated by ~50 Da. The feature could be a mixture of co-eluting species, at least one of which is not the C5 sulfonamide. This ion is not included with the list of identified features (SI Table S7) due to the ambiguity associated with its identity. The spectrum, however, is shown in SI Figure S2B2 with the perfluoroalkyl ether sulfonamide homologs, for reference.

In addition to the perfluorohexane ether sulfonamide isomers described at the end of Section 3.3 above, only the putative perfluoroheptane ether sulfonamide homolog was sufficiently abundant to generate a product ion spectrum in plasma in addition to the one observed from the dosing solution. The peak area of the perfluoroheptane ether sulfonamide homolog had a low abundance in plasma; however, it was more abundant in the dosing solution. The product ion spectra of the C6 homolog in plasma included only one perfluoroalkyl ether fragment ion which suggested the chemical was a single perfluoro(methoxy)hexane ether sulfonamide isomer. Similarly, only the methoxy isomer was observed for the C7 species in plasma. We identified the C7 species as CF_3_-O-C_6_F_12_-SO_2_NH_2_ (perfluoro-6-methoxyhexanesulfonamide) with level 2b confidence (expert assignment based on diagnostic analytical data). Spectra for the C8 and C9 homologues both include multiple perfluorinated alkoxylate fragment ions, indicating each was a co-eluting mixture of perfluoroalkyl ether sulfonamides.

Features with MS1 exact masses that corresponded to a homologous series of C6 to C9 perfluoroalkyl ether sulfonic acids were detected in rat plasma and liver. The proposed ether sulfonic acids were not detected in the dosing solution or PFHxSA standard. The presence of the perfluoroalkyl ether sulfonamides in the dosing solution and some biological samples, and the presence of the perfluoroalkyl ether sulfonic acids only in the rat tissue samples are consistent with the biotransformation of the dosed compound PFHxSA to PFHxS in plasma and liver. The product ion spectra for the perfluoroalkyl ether sulfonic acid series (SI Figure S2C) included ions annotated as SO_3_^−^ and FSO_3_^−^ that are characteristic of sulfonic acids. Spectra for the C6 and C8 homologs (SI Figures S2C1 and C3, respectively) exhibited multiple terminal perfluoroether fragments indicating the features included co-eluting multiple isomers with differing ether positions.

Legacy PFAS, including perfluorobutanesulfonamide (PFBSA) perfluorpentanesulfonamide (PFPeSA), perfluoroheptanesulfonamide (PFHpSA), and PFOSA, were also observed as impurities in the dosing solution with low abundance features also appearing in the biological samples. Peak areas for the legacy PFAS in the dosing solution were less than 4% of the PFHxSA signal. The co-occurring sulfonamides likely biotransformed in the rat to the legacy sulfonic acid BPs, including PFHpS and PFOS, that were detected in both liver and plasma. A feature having a mass consistent with perfluorobutane sulfonic acid was not detected. A feature that corresponded to perfluorononane sulfonic acid was observed with only trace abundance in plasma and liver. As with the perfluoroalkyl ether sulfonic acids, the legacy sulfonic acids were not detected in the dosing solution.

### 3.5. Feature Annotation and Identification of Proposed Polyfluorinated Biotransformation Products

**Figure 3:**
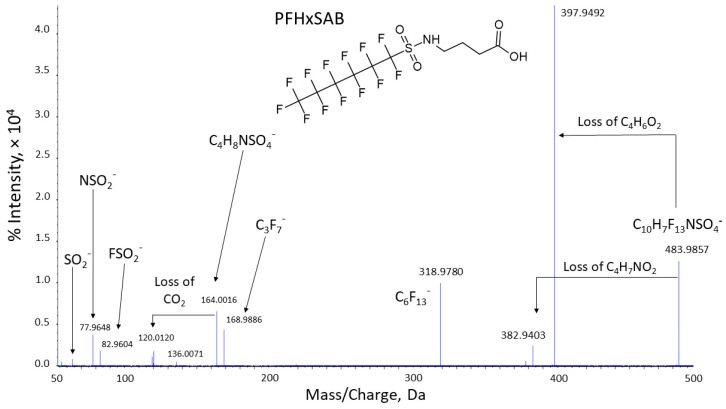
Product ion spectrum of the 4-[(perfluorohexane-1-sulfonyl)amino]butyric acid (PFHxSAB).

An abundant ion fitting the formula C_10_H_8_F_13_NO_4_S was observed in both plasma and liver. The ion was not detected in the dosing solution, suggesting it may be a BP. The product ion spectrum (Figure 3) included fragment ions resulting from neutral losses of C_4_H_6_O_2_ and C_4_H_7_NO_2_ from the headgroup of the molecular ion. Two other non-fluorinated ions were annotated as C_4_H_8_NSO_4_^−^ and C_2_H_8_NSO_2_^−^. The 44 Da mass difference between the two fragments is consistent with CO_2_ loss from C_4_H_8_NSO_4_^−^ which could occur through cleavage of an alkylcarboxylic acid tail from the sulfonamide headgroup. The losses of C_4_H_6_O_2_ and CO_2_ without evidence of other lower mass non-fluorinated fragmentation suggest that the amide nitrogen is bonded to single alkyl chain rather than to two alkyl chains. Characteristic PFAS fragments C_6_F_13_^−^ and C_3_F_7_^−^ and sulfonamide fragments SO_2_^−^, NSO_2_^−^, and FSO_2_^−^ were also present. Based on the annotated fragments shown in Figure 3 (see also SI Figure S2D3), we identified the C_10_H_8_F_13_NO_4_S species as a six-carbon C-F chain species with a four-carbon alkylcarboxy chain on the amide nitrogen: 4-[(perfluorohexane-1-sulfonyl)amino]butyric acid (PFHxSAB) with a level 2b confidence (expert assignment based on analytical data). The proposed chemical is not found in the CompTox Chemistry Dashboard [81] or PubChem [85]. Branched forms of the butyric acid headgroup or a di-alkylated amide cannot be definitively eliminated from consideration. Another isomer with a four-carbon headgroup, N-ethyl-N-((tridecafluorohexyl)sulfonyl)glycine (CAS: 68957-32-4; DTXSID0071889) can be disregarded since the acquired spectrum does not agree with the chemical’s AMOS reference spectrum [13].

Two other features of interest were observed in plasma only: *m*/*z* 433.9918 and *m*/*z* 455.9570. The product ion spectrum from *m*/*z* 433.9918 (SI Figure S2D1) included the same neutral losses as were observed for PFHxSAB, with all other fragments the same between the two spectra. Both species exhibited earlier retention times, which, along with the fragmentation similarities, lead to identification of the *m*/*z* 433.9918 feature as a C5 homolog of PFHxSAB with the formula C_9_H_8_F_11_NO_4_S. We identified the feature as 4-[perfluoropentane-1-sulphonyl)amino]butanoic acid) (PFPeSAB) (CAS: 68957-31-3; DTXSID 5071888).

The exact mass of the second feature, *m*/*z* 455.9570, matched with the formula C_8_H_4_F_13_NO_4_S. The product ion spectrum (SI Figure S2D2) includes the following annotations: characteristic sulfonic acid fragments, SO_2_^−^, NSO_2_^−^, and FSO_2_^−^; perfluoroalkyl chain fragments, C_2_F_5_^−^, C_3_F_7_^−^, and C_6_F_13_^−^; and fragments corresponding to PFHxSi^−^ and PFHxSA^−^, formed through loss of an alkyl chain from the amide moiety. With these findings, the feature can be identified as 2-([perfluorohexane-1-sulfonyl)amino)acetic acid (PFHxSAA; CAS: 1003193-99-4; DTXSID 401026647). Note that this chemical can also be named N-((perfluorohexyl)sulfonyl)glycine. The product ion spectrum from plasma agrees with the PFHxSAA spectrum in AMOS [13,82].

### 3.6. Distribution of PFHxSA and Predicted BPs

**Figure 4:**
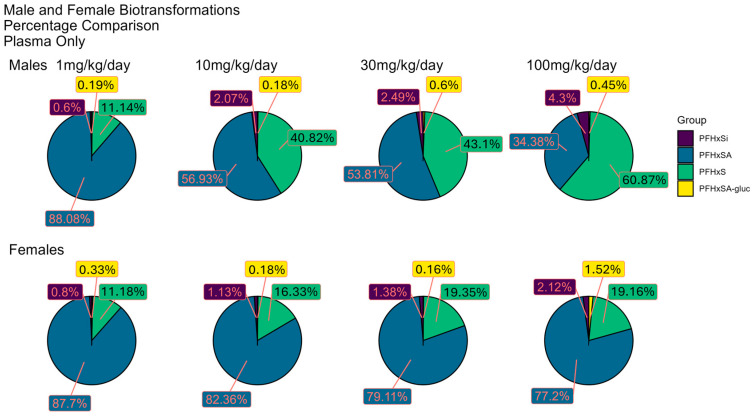
Relative distribution of peak areas for PFHxSA and biotransformation products in plasma from male and female rats dosed with PFHxSA. (PFHxSA-gluc: PFHxSA-N-glucuronide.) Refer to SI Figure S4A for box plots of peak areas for individual plasma samples by dose.

**Figure 5:**
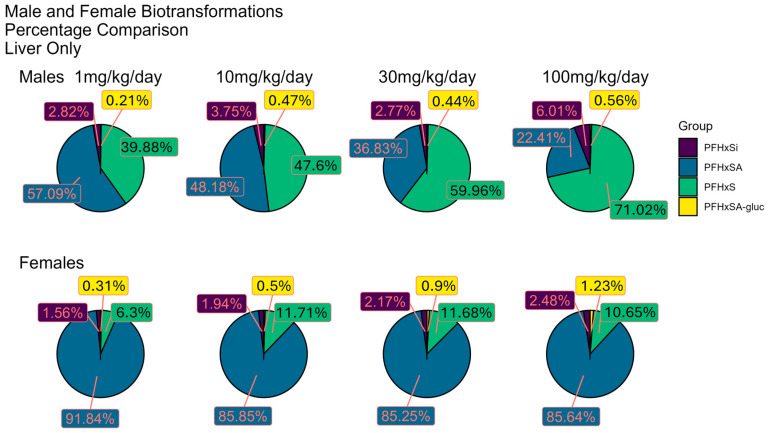
Relative distribution of peak areas for PFHxSA and biotransformation products in liver from male and female rats dosed with PFHxSA. (PFHxSA-gluc: PFHxSA-N-glucuronide.) Refer to SI Figure S4A for box plots of peak areas for individual plasma samples by dose.

The same PFHxSA biotransformation products were observed in rat plasma and liver; however, the distribution of the BPs differed by sex. The pie charts (Figure 4 and Figure 5) depict the distribution of PFHxSA and predicted BPs as the median percentage of the sum of their peak areas by sex and dose level. Median peak area is used here as an estimated measure of relative concentration, with the assumption that all species have similar ionization potentials and experience the same degree of suppression or enhancement in the ion source.

The proportion of PFHxSA relative to the BPs observed in both plasma and liver is higher for female rats than for male rats and is consistent with the measured values we reported in Bounds et al. [66]. The distribution of the dosed chemical and BPs differs between male and female rats. The percentage of PFHxSA observed in plasma from male rats dropped in a dose-dependent manner, from 88.1% at the lowest dose to 34.4% at the highest dose tested. Conversely, increases in the most abundant metabolite, PFHxS, were noted, with levels increasing from 11.1 to 40.8 to 60.9% of total median peak areas at 3, 10, and 100 mkd, respectively. This contrasts with the observation of little change in percentages of PFHxSA and PFHxS (~80% and ~20%, respectively) over the dose range in the plasma of female rats. PFHxSi, the next most abundant plasma BP, exhibited the highest percentage levels of 4.3 and 2.12% (males and females, respectively) at 100 mkd dosage. PFHxSA-N-glucuronide, with maxima of 0.6% in male rat plasma and 1.52% in female, is the least abundant BP. For female rats, the proportion of PFHxSA-N-glucuronide jumps by a factor of ten from the 10 mkd to the 30 mkd dose. The glucuronide conjugate is the only BP that has a higher percentage in female plasma compared male plasma, and only for the two highest doses.

As noted in male rat plasma, the percentage of liver PFHxSA decreases, and PFHxS increases, with increasing dose. The percentages of PFHxSA and PFHxS in female rat liver showed little change over the dose range with the values for PFHxSA and PFHxS calculated to be ~86% and ~11%, respectively. The percentage of PFHxSi increases over the dose range to 6% in male liver, whereas the proportion observed in female rat liver hovers at ~2%. The percentage of PFHxSA-N-glucuronide in liver is slightly higher for female rats than for males. The highest abundance of PFHxSA-N-glucuronide in female rat liver is 1.52% for the 30 mkd dose.

The novel PFAS ions identified as PFHxSAB and PFHxSAA are two of the most abundant co-occurring PFAS in plasma. The distribution pie charts of the median peak areas of these ions and the predicted BPs are shown in SI Figure S5. Box plots with peak areas of the ions in individual samples appear in SI Figure S4. For male rat plasma, the relative percentage of the novel PFHxSAB species rises from 0.23% at the 1 mkd dose to 3.4% at the 30 mkd dose before decreasing by about half to 1.53% at the high dose. PFHxSAB exhibits a similar response in female rat plasma. The PFHxSAA ion is present at the lowest percentage for males (<0.3%) and females (<0.1%) for all dose levels.

## 4. Discussion

With this study, we used NTA to evaluate biotransformation of PFHxSA in SD rats after a short-term exposure study. We analyzed plasma and liver from dosed and control animals. The dosing solution was also analyzed. The findings can be grouped into three categories: 1. Predicted PFHxSA BPs, 2. Dosing solution impurities and their BPs, and 3. Novel PFAS that are potential BPs.

### 4.1. Predicted PFHxSA Biotransformation Products

PFHxSA and three predicted BPs were observed in plasma and liver, with different distributions observed for tissues from male and female rats. PFHxSA and the BP PFHxS were predominant: NTA confirmed PFHxS as the most abundant metabolite with PFHxSi and PFHxS-N-glucuronide present at much lower levels (Figure 4 and Figure 5). The relative percentage of PFHxS was higher in male rats in both tissues, and at the two highest doses, the percentage was higher than that for PFHxSA. Both observations agree with our quantitative analysis [66] and reinforce the findings that the sex of the rat is a factor in exposure response.

The changing distributions of PFHxSA and PFHxS in male rat plasma and liver over the dose range provide insights into PFHxSA metabolism and clearance. In the liver, a dose-dependent increase in PFHxSA along with a concomitant increase in PFHxS was noted, with similar, albeit weaker, trends in the plasma. A shift is apparent between the 10 and 30 mkd doses: plasma levels at 1 and 10 mkd had approximately 53.8–56.9% PFHxSA, whereas levels decreased to 34–44% at higher doses, although not in a dose-dependent manner. These findings, in conjunction with a recent National Toxicology Program (NTP) PFHxSA study [86] where the benchmark dose (BMD) for relative liver weight changes was 16.25 mg/kg, suggest that hepatic liver enzyme induction is causing the PFHxS/PFHxSA shifts and differences observed. Indeed, recent PFHxSA transcriptomic evaluations conducted as a part of the ETAP study [29] showed, in males only, at least a 2-fold increase (FI) in mRNAs corresponding to CYPs 3A2 (2.34 FI), 4A1 (6.94 FI), 2J4 (2.8 FI), all with a lowest observed transcriptomic effect level (LOTEL) of 30 mkd. CYP1A1 mRNA was also upregulated by 4.43 FI, but with a LOTEL of 100 mkd. This evidence supplements previous reports that other CYP isoforms metabolize other PFASAs. In silico modeling and liver microsome exposures suggest the involvement of CYP450 in the biotransformation of some PFASA to the corresponding perfluoroalkylsulfonic acid [37,41]: typically, through N-dealkylation of other functional groups. Although induction of carboxylesterases, another candidate for amide hydrolysis, has been noted in the literature by PFOA, PFOS, and 7:1 FTOH, these enzymes were not upregulated in the Mutlu study and likely not involved in PFHxSA metabolism [87,88].

In essence, a study of a PFAS mixture, our evaluations of PFHxSA-PFHxS tissue levels and sex differences reveal unique patterns dictated by differing toxicokinetic considerations. Here, PFHxSA plasma and liver levels for female rats predominated over the other PFAS with little change in relative abundance across all four dose levels, whereas in males the percentage of PFHxSA present decreased with increasing dose, suggesting higher clearance/metabolism in males than females. These data are consistent with a recent NTP report, where PFHxSA half-lives were approximately 2-fold higher (i.e., the chemical is more stable) in the females than the males [86,89]. Conversely, PFHxS levels in males are consistently much higher than in females—on average 54% of the total liver distribution vs. 10% in the females—suggesting much higher accumulation in the males. With significant sex differences noted in PFHxSA-induced upregulation of several CYP isozymes, metabolism through this family is likely a key driver.

Transporter contribution to PFAS toxicokinetics is a rapidly emerging research area and has also been shown to be responsible for some sex differences in PFAS toxicokinetics. Renal reuptake has recently been documented for PFHxS, through organic anion transporter OAT4 [90,91]. Earlier evaluations have demonstrated hormonal control for others in this family, with male OAT2 and OAT3 mRNA levels much lower than females: responsible for basolateral uptake, these differences resulted in significantly higher PFOA renal elimination rates in females [92]. Indeed, male-female PFAS toxicokinetic comparison studies published to date show varied responses, with either females consistently displaying shorter (as for PFOA, PFHpA, PFBS, PFHpS, PFHxA) or similar (as for PFDA and PFOS) half-lives [93,94,95]. As some trends may be emerging, a greater understanding of transporter involvement and mixture effects are needed across the larger PFAS chemical space to ensure relevant toxicokinetic factors are adequately considered.

While metabolism of PFHxSA to the sulfonic acid species is widely recognized as the predominant biotransformation reaction, less-well-known is the metabolic generation of a perfluoroalkylsulfinic acid from PFASA. Formation of perfluoroalkylsulfinic acids is predicted to occur as an intermediate step in biotransformation of PFASA to perfluoroalkyl sulfonic acids [35]. The biotransformation to perfluoroalkylsulfinic acids has rarely been observed, however. A recent study reported PFHxSi in the liver of fish exposed to AFFF-impacted groundwater that also had PFHxSA and PFHxSi present [52], leaving it unclear as to whether biotransformation occurred in vivo. Here, NTA showed that PFHxSi occurred in rat plasma and liver at all doses levels after dosing with PFHxSA. PFHxSi was detected in the dosing solution and the PFHxSA standard at ~0.003% compared to PFHxSA. This low percentage suggests that PFHxSi present in the dosing solution is not entirely responsible for the ~3 orders of magnitude higher abundances in plasma and liver (see Figure 4 and Figure 5). As PFHxSi is an intermediate in the pathway to PFHxS formation, the higher percentages observed in males for both tissues and all doses are likely related to the previously described sex differences for this pathway. To our knowledge, this is the first report of biotransformation of PFHxSA to PFHxSi in mammals.

NTA indicated occurrence of a third predicted biotransformation product—PFHxSA-N-glucuronide. We observed features corresponding to PFHxSA-N-glucuronide eluting at three separate retention times. The product ion spectra from the individual peaks were highly similar and suggest the chromatographic separation is due to the presence of br- and n-PFHxSA species, with the linear species having the highest abundance and eluting last. It was not clear by LC/MS/MS whether the linear isomer of PFHxSA was also more abundant than the branched isomers as the isomers did not separate chromatographically. However, synthesis of PFHxS by ECF has been reported to produce approximately 95% n-PFHxS [96] and the pre-administration GC/MS analysis of the PFHxSA standard here indicated n-PFHxSA comprised 78.4% of the isomeric distribution. Detection of the PFHxSA-N-glucuronide after administration of PFHxSA to SD rats adds support to reports that the Phase II formation of the N-glucuronide conjugate is well conserved across widely varied species [35]. N-glucuronidation of short chain PFASAs including PFHxSA was recently demonstrated in mice [59] by the detection of glucuronide conjugates in urine—though not in mouse liver or kidney.

In the current study, PFHxSA-N-glucuronide was detected in both plasma and liver. The highest percentage contribution of PFHxSA-N-glucuronide to the total PFHxSA and BP signal was the 1.52% observed in plasma from female rats at the 30 mkd dose level. In contrast to the higher percent abundances for PFHxSA, PFHxS, and PFHxSi in plasma and liver from male rats, the abundance percentage of PFHxSA-N-glucuronide was equal to or higher in tissues from female rats. This is likely secondary to the fact that in males the primary biotransformation route is PFHxS formation, resulting in lower levels of PFHxSA and as a result lower levels of the glucuronidated sulfonamide—not due to any independent sex differences in glucuronidation.

### 4.2. Dosing Solution Impurities and Their Biotransformation Products

We observed PFHxS in the dosing solution at ~3% compared to the average peak area of PFHxSA. It is unclear if the PFHxS observed comes from abiotic degradation of PFHxSA or results from the side reactions during synthesis of the parent compound. Chemical modeling of PFHxSA and related PFHxS shows high bond dissociation energies and suggest it should degrade to PFHxA and PFBA rather than PFHxS by photodegradation [39], though neither carboxylic acid species was observed here. However, the amount of PFHxS present in the dosing solution is sufficiently low, so as not to contribute significantly to the total amount observed in vivo.

Trace levels of perfluoroalkyl ether sulfonamides were detected in the plasma, liver, and dosing solution. Presence of the chemicals in the dosing solution suggests they are impurities that were dosed along with PFHxSA and not biotransformation products. The source material for PFASA, perfluoroalkylsulfonyl fluorides, are synthesized by electrochemical fluorination (ECF), a process that gives decreasing yields of intended product with increasing chain lengths and leads to a mixture that includes both branched and linear species. Perfluoroalkyl ether sulfonamides potentially may be formed during electrochemical fluorination [97]. Subsequent use of perfluoroalkylsulfonyl fluorides as starting materials for PFASA would be accompanied by formation of multiple byproducts that may generate some of the impurities observed in the dosing solution. Interestingly, multiple ether isomers, in which the position of the ether moved along the C-F chain, were observed for all except the C7 homolog in the dosing solution; however, only perfluoro methoxy ether sulfonamides were detected in the plasma for the two homologs that were sufficiently abundant to generate product ion spectra. It may be that the perfluoro methoxy sulfonamides were the most abundant of the isomers in the dosing solution and not preferentially converted to BPs in the rat, leaving some intact species to be detected in plasma. An orthogonal separation method such as ion mobility with a more concentrated sample may provide clarity on the composition of the isomer distribution.

Perfluoroalkyl ether sulfonic acid homologs (C6 through C9) corresponding to BPs of the perfluoroalkyl ether sulfonamides in the dosing solution were detected in plasma and liver. The product ion spectra provided evidence for the presence of multiple ether sulfonic acids isomers in the C6 through C8 homologs but only an ethoxy sulfonic acid for the low abundance C9 entity.

### 4.3. Novel Potential Biotransformation Products PFPeSAB, PFHxSAA, and PFHxSAB

The sources of the species proposed as the (M-H)^−^ ions of PFPeSAB, PFHxSAA, and PFHxSAB in plasma and liver of rats dosed with PFHxSA are not known. The chemicals may be BPs of precursors such as sulfonamido-alcohols or aldehydes that would be incompatible with detection by LC/MS/MS as they would likely decompose in an aqueous environment. N-EtFOSA-alcohol and N-EtFOSA-aldehyde were proposed as intermediates during transformation of N-EtFOSA to FOSAA in aerobic soil [35]. Here, the PFASA precursors could be mono-N-alkylated and would be expected to be observed by LC/MS. Mono-N-hydroxy alkylated FASA intermediates, however, would be more amenable to detection by GC/MS. Sulfonamide synthesis could occur through addition of a primary or secondary amine to the perfluoroalkylsulfonyl fluoride followed by cleavage of the amine side chain [98,99]. Mono-N-alkylated species could occur due to incomplete cleavage. For example, the formation of perfluoro-N-(4-hydroxybutyl) hexane-1-sulfonamide (C_10_H_10_F_13_NO_3_S; PubChem CID 141055320) was reported to occur during synthesis of fluorochemical esters [100]. The presence of this compound along with a perfluoropentane sulfonamide with a hydroxybutyl headgroup and perfluorobutane-1-sulfonamidoethanol (FBSE) (CAS 34454-99-4; DTXSID00881351) as impurities in the dosing solution, could lead to oxidation by an alcohol dehydrogenase in the rat to the perfluoroalkyl-N-(alkylcarboxylic) sulfonamides suggested by the product ion spectra. None of these compounds have known toxicity profiles.

Interestingly, the feature proposed as PFHxSAB has an amino butyric acid headgroup that, depending on the isomeric form, shares structural similarity to GABA (δ-aminobutyric acid), BABA (β-aminobutyric acid), and AABA (α-aminobutyric acid) which are bioactive neurotransmitters. A variety of PFAS have been associated with neurotoxicity [101,102,103,104], and some have been shown to act as GABA receptor agonists [105]. It may be possible that PFHxSAB could bind with GABA receptors and disrupt neurotransmitter homeostasis. It is also conceivable that at high concentrations, PFHxSA could provide an additional nitrogen source for the glutamate decarboxylase pathway of the GABA synthesis cycle [106,107]. In vitro studies may provide clarity on the potential for GABA receptor binding and interference with GABA biosynthesis.

## 5. Conclusions

This study aimed to understand biotransformations of a single PFAS in rats and evolved into an evaluation of a complex mixture of the dosed chemical and its BPs plus a variety of minor impurities and their BPs. These findings emphasize not only the need to investigate toxicity of mixtures of PFAS but also to use NTA for discovery and identification of unknown and unexpected chemicals present in the samples as well as the standard of the dosed chemical and the dosing formulation. By using NTA, we observed the BPs of PFHxSA, including the rarely observed PFHxSi, and legacy PFAS and their BPs, including PFOSi, homologs of perfluoroalkyl ether sulfonamides and sulfonic acids, and unusual mono-N-alkylcarboxylate perfluoroalkyl sulfonamides. To our knowledge, PFHxSi and PFOSi have not been previously reported as BPs in mammals. In addition, we are not aware of previous reports of detection of PFHxSAA and the novel compounds PFHxSAB and PFPeSAB from in vivo exposure studies.

Most of the PFAS observed in the rat tissues as well as the trace level PFAS detected in the dosing solution have unknown toxicological profiles. And as most do not have commercially available standards, targeted analytical methods and accurate quantitation are hampered. NTA with GC/MS could reveal chemicals missed by LC/MS and clarify the sources of some of the questions about precursors. Follow-on in vitro studies with purified materials may help differentiate the contributions of the individual PFAS to the toxicity of the mixture. Though, because PFAS occur as mixtures in the environment, knowledge of effects of exposure to multiple PFAS and their biotransformation products together is important. Additionally, in vitro studies sufficiently robust to evaluate human and rat biotransformation of lower turnover PFAS are critically needed to inform cross-species comparisons and data extrapolation.

## Data Availability

Considering the funding of this effort by the US EPA and in compliance with the US EPA Public Access policy, the accepted, non-formatted version of the accepted manuscript and any associated data files will be made available on PubMed Central one year after acceptance by the journal.

## Supplementary Materials

The following supporting information can be downloaded at: https://www.mdpi.com/article/10.3390/toxics13070523/s1. Table S1: Liquid Chromatography Gradient; Table S2: MS Instrument Conditions; Table S3: NTA Sciex OS Data Processing Parameters; Table S4: MarkerView Data Processing Parameters; Table S5: INTERPRET NTA Data Processing Parameters; Table S6: PFHxSA Predicted Biotransformation Products; Table S7: Feature Identifications. Figure S1: NTA Batch Quality Control. (A): Plasma Internal Standard Peak Areas (B): Coefficient of Variation (CV) vs Abundance (C): Occurrence Heatmap, Sample Replicate Threshold 66% (D): Occurrence Heatmap, Sample Replicate Threshold 100%; Figure S2: Annotated Spectra of Select Features (See Figure 2 Annotated Spectra.pdf). (A): Perfluoroalkyl sulfinic acid homologues (B): Perfluoroalkyl ether sulfonamides (C): Perfluoroalkyl ether sulfonic acid homologues (D): [(Perfluoroalkyl-1-sulfonyl)amido]alkylcarboxylic acids; Figure S3: PFHxSA-N-glucuronide extracted ion chromatogram from a high dose plasma sample; Figure S4: Peak Areas for Detections in Plasma and Liver. (A): Plasma (See Figure S4A-Peak Areas for Detections in Plasma.pdf) (B): Liver (See Figure S4B-Peak Areas for Detections in Liver.pdf); Figure S5: Median percent of total peak areas for 6 co-occuring PFAS.

## References

[1] Strynar M.J. (2025). A paradigm shift in environmental monitoring—The time for non-targeted analysis (NTA) is now. Environ. Int..

[2] Black G., Lowe C., Anumol T., Bade J., Favela K., Feng Y.-L., Knolhoff A., McEachran A., Nuñez J., Fisher C. (2023). Exploring chemical space in non-targeted analysis: A proposed ChemSpace tool. Anal. Bioanal. Chem..

[3] Reymond J.-L. (2015). The Chemical Space Project. Acc. Chem. Res..

[4] Strynar M.J., Dagnino S., McMahen R., Liang S., Lindstrom A., Andersen E., McMillan L., Thurman M., Ferrer I., Ball C. (2015). Identification of novel perfluoroalkyl ether carboxylic acids (PFECAs) and sulfonic acids (PFESAs) in natural waters using accurate mass time-of-flight mass spectrometry (TOFMS). Environ. Sci. Tech..

[5] McCord J.P., Strynar M.J., Washington J.W., Bergman E.L., Goodrow S.M. (2020). Emerging Chlorinated Polyfluorinated Polyether Compounds Impacting the Waters of Southwestern New Jersey Identified by Use of Nontargeted Analysis. Environ. Sci. Technol. Lett..

[6] Evich M.G., Davis M.J.B., McCord J.P., Acrey B., Awkerman J.A., Knappe D.R.U., Lindstrom A.B., Speth T.s.F., Tebes-Stevens C., Strynar M.J. (2022). Per- and polyfluoroalkyl substances in the environment. Science.

[7] Megson D., Niepsch D., Spencer J., dos Santos C., Florance H., MacLeod C., Ross I. (2024). Non-targeted analysis reveals hundreds of per- and polyfluoroalkyl substances (PFAS) in UK freshwater in the vicinity of a fluorochemical plant. Chemosphere.

[8] Bangma J., Guillette T.C., Strynar M.J., Lindstrom A.B., McCord J.P., Hill D., Lau C., Chernoff N., Lang J.R. (2022). A rapid assessment bioaccumulation screening (RABS) study design for emerging per-and polyfluoroalkyl substances in mice exposed to industrially impacted surface water. Chemosphere.

[9] Boettger J.D., DeLuca N.M., Zurek-Ost M.A., Miller K.E., Fuller C., Bradham K.D., Ashley P., Friedman W., Pinzer E.A., Cox D.C. (2025). Emerging Per- and Polyfluoroalkyl Substances in Tap Water from the American Healthy Homes Survey II. Environ. Sci. Technol..

[10] Manz K.E., Feerick A., Braun J.M., Feng Y.-L., Hall A., Koelmel J., Manzano C., Newton S.R., Pennell K.D., Place B.J. (2023). Non-targeted analysis (NTA) and suspect screening analysis (SSA): A review of examining the chemical exposome. J. Expo. Sci. Environ. Epidemiol..

[11] Liu J., Zhong G., Li W., Mejia Avendaño S. (2019). Isomer-specific biotransformation of perfluoroalkyl sulfonamide compounds in aerobic soil. Sci. Tot. Environ..

[12] Whitehead H., Buckley T., Sobus J., Bangma J., MacMillan D., Williams A., Janesch G., Coombs J., Newman E., Dahlmeier A. (2025). Non-Targeted Analysis of Surface and Groundwaters Impacted by Historic PFAS Waste Sites. Environ. Sci. Technol. Accept..

[13] Whitehead H.D., Buckley T.J., Sobus J.R., Bangma J., MacMillan D.K., Ferland T.M., Chao A., Williams A.J., Janesch G., Cofield K. (2025). The ENTAiLS Toolkit: An Integrated Workflow to Perform Non-Targeted Analysis of Per- and Polyfluoroalkyl Substances. Anal. Bioanal. Chem..

[14] Curtzwiler G.W., Silva P., Hall A., Ivey A., Vorst K. (2021). Significance of Perfluoroalkyl Substances (PFAS) in Food Packagin. Integr. Environ. Assess. Manag..

[15] Seltenrich N. (2020). PFAS in Food Packaging: A Hot, Greasy Exposure. Environ. Health Perspect..

[16] Harris K.J., Munoz G., Woo V., Sauvé S., Rand A.A. (2022). Targeted and Suspect Screening of Per- and Polyfluoroalkyl Substances in Cosmetics and Personal Care Products. Environ. Sci. Technol..

[17] Annunziato K.M., Doherty J., Lee J., Clark J.M., Liang W., Clark C.W., Nguyen M., Roy M.A., Timme-Laragy A.R. (2020). Chemical Characterization of a Legacy Aqueous Film-Forming Foam Sample and Developmental Toxicity in Zebrafish (*Danio rerio*). Environ. Health Perspect..

[18] Dubocq F., Wang T., Yeung L.W.Y., Sjöberg V., Kärrman A. (2020). Characterization of the Chemical Contents of Fluorinated and Fluorine-Free Firefighting Foams Using a Novel Workflow Combining Nontarget Screening and Total Fluorine Analysis. Environ. Sci. Technol..

[19] Megson D., Bruce-Vanderpuije P., Idowu I.G., Ekpe O.D., Sandau C.D. (2025). A systematic review for non-targeted analysis of per- and polyfluoroalkyl substances (PFAS). Sci. Tot. Environ..

[20] Ruyle B.J., Thackray C.P., McCord J.P., Strynar M.J., Mauge-Lewis K.A., Fenton S.E., Sunderland E.M. (2021). Reconstructing the Composition of Per- and Polyfluoroalkyl Substances in Contemporary Aqueous Film-Forming Foams. Environ. Sci. Technol. Lett..

[21] Choi Y.J., Helbling D.E., Liu J., Olivares C.I., Higgins C.P. (2022). Microbial biotransformation of aqueous film-forming foam derived polyfluoroalkyl substances. Sci. Tot. Environ..

[22] Renyer A., Ravindra K., Wetmore B.A., Ford J.L., DeVito M., Hughes M.F., Wehmas L.C., MacMillan D.K. (2023). Dose Response, Dosimetric, and Metabolic Evaluations of Replacement PFAS Perfluoro-(2,5,8-trimethyl-3,6,9-trioxadodecanoic) Acid (HFPO-TeA). Toxics.

[23] Ye L., Li J., Gong S., Herczegh S.M., Zhang Q., Letcher R.J., Su G. (2023). Established and emerging organophosphate esters (OPEs) and the expansion of an environmental contamination issue: A review and future directions. J. Haz. Mater..

[24] Chang D., Cowden J., Davidson-Fritz S., Dean J., Devito M., Everett L., Harrill A., Hester S., Hughes M., Lambert J. (2024). Scientific Studies Supporting Development of Transcriptomic Points of Departure for EPA Transcriptomic Assessment Products (ETAPs). https://epa.figshare.com/articles/online_resource/Scientific_Studies_Supporting_Development_of_Transcriptomic_Points_of_Departure_for_EPA_Transcriptomic_Assessment_Products_ETAPs_/25365550.

[25] Brennan A., Chang D., Cowden J., Davidson-Fritz S., Dean J., Devito M., Ford J., Everett L., Harrill A., Hester S. (2024). Standard Methods for Development of EPA Transcriptomic Assessment Products (ETAPs). https://epa.figshare.com/articles/online_resource/Standard_Methods_for_Development_of_EPA_Transcriptomic_Assessment_Products_ETAPs_/25365496/1.

[26] Patlewicz G., Richard A.M., Williams A.J., Grulke C.M., Sams R., Lambert J., Noyes P.D., DeVito M.J., Hines R.N., Strynar M. (2019). A Chemical Category-Based Prioritization Approach for Selecting 75 Per- and Polyfluoroalkyl Substances (PFAS) for Tiered Toxicity and Toxicokinetic Testing. Environ. Health Perspect..

[27] Patlewicz G., Richard A.M., Williams A.J., Judson R.S., Thomas R.S. (2022). Towards reproducible structure-based chemical categories for PFAS to inform and evaluate toxicity and toxicokinetic testing. Comput. Toxicol..

[28] Patlewicz G., Shah I. (2023). Towards systematic read-across using Generalised Read-Across (GenRA). Comput. Toxicol..

[29] Mutlu E., Wehmas L.C., Harrill A.H., DeVito M.J., Thomas R.S., Hughes M.F., MacMillan D.K., Brennan A.A., Bounds J.G., Weitekemp C.A. (2025). Transcriptomic dose response of PFAS chemicals 3:3 fluorotelomer carboxylic acid, 7:3 fluorotelomer alcohol, and perfluorohexanesulfonamide. Toxicology.

[30] Glüge J., Scheringer M., Cousins I.T., DeWitt J.C., Goldenman G., Herzke D., Lohmann R., Ng C.A., Trier X., Wang Z. (2020). An overview of the uses of per- and polyfluoroalkyl substances (PFAS). Environ. Sci. Process. Impacts.

[31] McDonough C.A., Choyke S., Barton K.E., Mass S., Starling A.P., Adgate J.L., Higgins C.P. (2021). Unsaturated PFOS and Other PFASs in Human Serum and Drinking Water from an AFFF-Impacted Community. Environ. Sci. Technol..

[32] Munoz G., Mercier L., Duy S.V., Liu J., Sauvé S., Houde M. (2022). Bioaccumulation and trophic magnification of emerging and legacy per- and polyfluoroalkyl substances (PFAS) in a St. Lawrence River food web. Environ. Pollut..

[33] All POPs Listed in the Stockholm Convention. http://chm.pops.int/TheConvention/ThePOPs/AllPOPs/tabid/2509/Default.aspx.

[34] Carrizo J.C., Munoz G., Vo Duy S., Liu M., Houde M., Amé M.V., Liu J., Sauvé S. (2023). PFAS in fish from AFFF-impacted environments: Analytical method development and field application at a Canadian international civilian airport. Sci. Tot. Environ..

[35] Kolanczyk R.C., Saley M.R., Serrano J.A., Daley S.M., Tapper M.A. (2023). PFAS Biotransformation Pathways: A Species Comparison Study. Toxics.

[36] Liu S.D., Dukes D.A., Koelmel J.P., Stelben P., Finch J., Okeme J., Lowe C., Williams A., Godri D., Rennie E.E. (2025). Expanding PFAS Identification with Transformation Product Libraries: Nontargeted Analysis Reveals Biotransformation Products in Mice. Environ. Sci. Technol..

[37] Fu Z., Wang Y., Wang Z., Xie H., Chen J. (2015). Transformation Pathways of Isomeric Perfluorooctanesulfonate Precursors Catalyzed by the Active Species of P450 Enzymes: In Silico Investigation. Chem. Res. Toxicol..

[38] Benskin J.P., De Silva A.O., Martin L.J., Arsenault G., McCrindle R., Riddell N., Mabury S.A., Martin J.W. (2009). Disposition of perfluorinated acid isomers in Sprague-Dawley rats; part 1: Single dose. Environ. Toxicol. Chem..

[39] Zhong H., Liu W., Li N., Ma D., Zhao C., Li J., Wang Y., Jiang G. (2022). Assessment of perfluorohexane sulfonic acid (PFHxS)-related compounds degradation potential: Computational and experimental approaches. J. Haz. Mater..

[40] Han J., Gu W., Barrett H., Yang D., Tang S., Sun J., Liu J., Krause H.M., Houck K.A., Peng H.A. (2021). A Roadmap to the Structure-Related Metabolism Pathways of Per- and Polyfluoroalkyl Substances in the Early Life Stages of Zebrafish (*Danio rerio*). Environ. Health Perspect..

[41] Tomy G.T., Tittlemier S.A., Palace V.P., Budakowski W.R., Braekevelt E., Brinkworth L., Friesen K. (2004). Biotransformation of N-ethyl perfluorooctanesulfonamide by rainbow trout (*Onchorhynchus mykiss*) liver microsomes. Environ. Sci. Technol..

[42] Xu L., Krenitsky D.M., Seacat A.M., Butenhoff J.L., Anders M.W. (2004). Biotransformation of N-ethyl-N-(2-hydroxyethyl)perfluorooetanesulfonamide by rat liver microsomes, cytosol, and slices and by expressed rat and human cytochromes P450. Chem. Res. Toxicol..

[43] Xu L., Krenitsky D.M., Seacat A.M., Butenhoff J.L., Tephly T.R., Anders M.W. (2006). N-glucuronidation of perfluorooctanesulfonamide by human, rat, dog, and monkey liver microsomes and by expressed rat and human UDP-glucuronosyltransferases. Drug Metab. Dispos. Biol. Fate Chem..

[44] Ross M.S., Wong C.S., Martin J.W. (2012). Isomer-specific biotransformation of perfluorooctane sulfonamide in Sprague-Dawley rats. Environemental Sci. Technol..

[45] Letcher R.J., Chu S., McKinney M.A., Tomy G.T., Sonne C., Dietz R. (2014). Comparative hepatic in vitro depletion and metabolite formation of major perfluorooctane sulfonate precursors in arctic polar bear, beluga whale and ringed seal. Chemosphere.

[46] Zhao S., Wang B., Zhong Z., Liu T., Liang T., Zhan J. (2020). Contributions of enzymes and gut microbes to biotransformation of perfluorooctane sulfonamide in earthworms (*Eisneia fetida*). Chemosphere.

[47] Zhao S., Liang T., Zhou T., Li D., Wang B., Zhan J., Liu L. (2018). Biotransformation and responses of antioxidant enzymes in hydroponically cultured soybean and pumpkin exposed to perfluorooctane sulfonamide (FOSA). Ecotoxicol. Environ. Saf..

[48] Zhao S., Liang T., Zhu L., Yang L., Liu T., Fu J., Wang B., Zhan J., Liu L. (2019). Fate of 6:2 fluorotelomer sulfonic acid in pumpkin (*Cucurbita maxima* L.) based on hydroponic culture: Uptake, translocation and biotransformation. Environ. Pollut..

[49] Lange C.C. (2000). Biodegradation Study Report: The Aerobic Biodegradation of N-EtFOSE Alcohol by the Microbial Activity Present in Municipal Wastewater Treatment Sludge. https://static.ewg.org/reports/2003/pfcs/226-1030a078.pdf.

[50] Rhoads K.R., Janssen E.M.-L., Luthy R.G., Criddle C.S. (2008). Aerobic Biotransformation and Fate of N-Ethyl Perfluorooctane Sulfonamidoethanol (N-EtFOSE) in Activated Sludge. Environ. Sci. Technol..

[51] Avendano S.J., Liu J. (2015). Production of PFOS from aerobic soil biotransformation of two perfluoroalkyl sulfonamide derivatives. Chemosphere.

[52] Hill N.I., Becanova J., Vojta S., Barber L.B., LeBlanc D.R., Vajda A.M., Pickard H.M., Lohmann R. (2024). Bioconcentration of Per- and Polyfluoroalkyl Substances and Precursors in Fathead Minnow Tissues Environmentally Exposed to Aqueous Film-Forming Foam–Contaminated Waters. Environ. Toxicol. Chem..

[53] Dooley M.R., Nixon S.P., Payton B.E., Hudak M.A., Odei F., Vyas S. (2024). Atmospheric Oxidation of PFAS by Hydroxyl Radical: A Density Functional Theory Study. Environ. Sci. Technol. Air.

[54] Seger S.T., Rydberg P., Olsen L. (2015). Mechanism of the N-hydroxylation of primary and secondary amines by cytochrome P450. Chem. Res. Toxicol..

[55] Zhang H., Wang X., Song R., Ding W., Li F., Ji L. (2023). Emerging Metabolic Profiles of Sulfonamide Antibiotics by Cytochromes P450: A Computational–Experimental Synergy Study on Emerging Pollutants. Environ. Sci. Technol..

[56] Almazroo O.A., Miah M.K., Venkataramanan R. (2017). Drug metabolism in the liver. Clin. Liver Dis..

[57] Issa N.T., Wathieu H., Ojo A., Byers S.W., Dakshanamurthy S. (2017). Drug Metabolism in Preclinical Drug Development: A Survey of the Discovery Process, Toxicology, and Computational Tools. Curr. Drug Metab..

[58] Ali J.M., Roberts S.M., Gordon D.S., Stuchal L.D. (2019). Derivation of a chronic reference dose for perfluorohexane sulfonate (PFHxS) for reproductive toxicity in mice. Regul. Toxicol. Pharmacol..

[59] Dukes D.A., McDonough C.A. (2024). N-glucuronidation and Excretion of Perfluoroalkyl Sulfonamides in Mice Following Ingestion of Aqueous Film-Forming Foam. Environ. Toxicol. Chem..

[60] Pickard H.M., Haque F., Sunderland E.M. (2024). Bioaccumulation of Perfluoroalkyl Sulfonamides (FASA). Environ. Sci. Technol. Lett..

[61] Holder C., DeLuca N., Luh J., Alexander P., Minucci J.M., Vallero D.A., Thomas K., Cohen Hubal E.A. (2023). Systematic Evidence Mapping of Potential Exposure Pathways for Per- and Polyfluoroalkyl Substances Based on Measured Occurrence in Multiple Media. Environ. Sci. Technol..

[62] (2011). Guide for the Care and Use of Laboratory Animals: Eighth Edition.

[63] Kilkenny C., Browne W.J., Cuthill I.C., Emerson M., Altman D.G. (2010). Improving Bioscience Research Reporting: The ARRIVE Guidelines for Reporting Animal Research. PLoS Biol..

[64] Percie du Sert N., Hurst V., Ahluwalia A., Alam S., Avey M.T., Baker M., Browne W.J., Clark A., Cuthill I.C., Dirnagl U. (2020). The ARRIVE guidelines 2.0: Updated guidelines for reporting animal research. PLoS Biol..

[65] (2020). Guidelines for the Euthanasia of Animals.

[66] Bounds J.G., Renyer A., Brennan A.A., Ford J.L., Ravindra K., Wetmore B.A., Devito M., Hughes M.F., Wehmas L.C., MacMillan D.K. (2025). Evaluations of Thyroid Effects, Dosimetry, and Metabolism following Perfluorohexanesulfonamide Exposure in Sprague Dawley Rats. Toxics.

[67] Lambert J.P., Ivosev G., Couzens A.L., Larsen B., Taipale M., Lin Z.Y., Zhong Q., Lindquist S., Vidal M., Aebersold R. (2013). Mapping differential interactomes by affinity purification coupled with data-independent mass spectrometry acquisition. Nat. Methods.

[68] Roemmelt A.T., Steuer A.E., Poetzsch M., Kraemer T. (2014). Liquid Chromatography, in Combination with a Quadrupole Time-of-Flight Instrument (LC QTOF), with Sequential Window Acquisition of All Theoretical Fragment-Ion Spectra (SWATH) Acquisition: Systematic Studies on Its Use for Screenings in Clinical and Forensic Toxicology and Comparison with Information-Dependent Acquisition (IDA). Anal. Chem..

[69] Peter K.T., Phillips A.L., Knolhoff A.M., Gardinali P.R., Manzano C.A., Miller K.E., Pristner M.l., Sabourin L., Sumarah M.W., Warth B. (2021). Nontargeted Analysis Study Reporting Tool: A Framework to Improve Research Transparency and Reproducibility. Anal. Chem..

[70] USEPA Chemical Transformation Simulator. https://qed.epa.gov/cts/.

[71] Djoumbou-Feunang Y., Fiamoncini J., Gil-de-la-Fuente A., Greiner R., Manach C., Wishart D.S. (2019). BioTransformer: A comprehensive computational tool for small molecule metabolism prediction and metabolite identification. J. Cheminform..

[72] Weber E.J., Tebes-Stevens C., Washington J.W., Gladstone R. (2022). Development of a PFAS reaction library: Identifying plausible transformation pathways in environmental and biological systems. Env. Sci. Process Impacts.

[73] Wolf S., Schmidt S., Müller-Hannemann M., Neumann S. (2010). In silico fragmentation for computer assisted identification of metabolite mass spectra. BMC Bioinform..

[74] Ruttkies C., Schymanski E.L., Wolf S., Hollender J., Neumann S. (2016). MetFrag relaunched: Incorporating strategies beyond in silico fragmentation. J. Cheminform..

[75] Ruttkies C., Neumann S., Posch S. (2019). Improving MetFrag with statistical learning of fragment annotations. BMC Bioinform..

[76] Schmid R., Heuckeroth S., Korf A., Smirnov A., Myers O., Dyrlund T.S., Bushuiev R., Murray K.J., Hoffmann N., Lu M. (2023). Integrative analysis of multimodal mass spectrometry data in MZmine 3. Nat. Biotechnol..

[77] Heuckeroth S., Damiani T., Smirnov A., Mokshyna O., Brungs C., Korf A., Smith J.D., Stincone P., Dreolin N., Nothias L.-F. (2024). Reproducible mass spectrometry data processing and compound annotation in MZmine 3. Nat. Protoc..

[78] Deutsch E.W. (2010). Mass Spectrometer Output File Format mzML. Methods Mol. Biol..

[79] Chambers M.C., Maclean B., Burke R., Amode D., Ruderman D.L., Neumann S., Gatto L., Fischer B., Pratt B., Egertson J. (2012). A cross-platform toolkit for mass spectrometry and proteomics. Nat. Biotechnol..

[80] Sobus J.R., Sayre-Smith N.A., Chao A., Ferland T.M., Minucci J.M., Carr E.T., Brunelle L.D., Batt A.L., Whitehead H.D., Cathey T. (2025). Automated QA/QC reporting for non-targeted analysis: A demonstration of “INTERPRET NTA” with de facto water reuse data. Anal. Bioanal. Chem..

[81] Williams A.J., Grulke C.M., Edwards J., McEachran A.D., Mansouri K., Baker N.C., Patlewicz G., Shah I., Wambaugh J.F., Judson R.S. (2017). The CompTox Chemistry Dashboard: A community data resource for environmental chemistry. J. Chemoinform..

[82] Janesch G., Carr E.T., Sivasupramaniam S., Charest N., Tkachenko V., Williams A.J. (2025). Applying Chemoinformatics to Develop a Structure Searchable Database of Analytical Methods. JoVE.

[83] Schymanski E.L., Jeon J., Gulde R., Fenner K., Ruff M., Singer H.P., Hollender J. (2014). Identifying Small Molecules via High Resolution Mass Spectrometry: Communicating Confidence. Environ. Sci. Technol..

[84] Igarashi K. (1977). The Koenigs-Knorr Reaction. Advances in Carbohydrate Chemistry and Biochemistr.

[85] Kim S., Chen J., Cheng T., Gindulyte A., He J., He S., Li Q., Shoemaker B.A., Thiessen P.A., Yu B. (2025). PubChem 2025 update. Nucleic Acids Res..

[86] Auerbach S.S., Ballin J.D., Blake J.C., Browning D.B., Collins B.J., Cora M.C., Fernando R.A., Fostel J.M., Liu Y.F., Luh J. (2023). Report on the In Vivo Repeat Dose Biological Potency Study of Perfluorohexanesulfonamide (CASRN 41997-13-1) in Sprague Dawley (Hsd:Sprague Dawley® SD®) Rats (Gavage Studies). NIEHS Report 10.

[87] Berthiaume J., Wallace K.B. (2002). Perfluorooctanoate, perflourooctanesulfonate, and N-ethyl perfluorooctanesulfonamido ethanol; peroxisome proliferation and mitochondrial biogenesis. Toxicol. Lett..

[88] Derbel M., Hosokawa M., Satoh T. (1996). Differences in the induction of carboxylesterase RL4 in rat liver microsomes by various perfluorinated fatty acids, metabolically inert derivatives of fatty acids. Biol. Pharm. Bull..

[89] Crizer D.M., Rice J.R., Smeltz M.G., Lavrich K.S., Ravindra K., Wambaugh J.F., DeVito M., Wetmore B.A. (2024). In Vitro Hepatic Clearance Evaluations of Per- and Polyfluoroalkyl Substances (PFAS) across Multiple Structural Categories. Toxics.

[90] Ryu S., Yamaguchi E., Sadegh Modaresi S.M., Agudelo J., Costales C., West M.A., Fischer F., Slitt A.L. (2024). Evaluation of 14 PFAS for permeability and organic anion transporter interactions: Implications for renal clearance in humans. Chemosphere.

[91] Louisse J., Dellafiora L., van den Heuvel J.J.M.W., Rijkers D., Leenders L., Dorne J.C.M., Punt A., Russel F.G.M., Koenderink J.B. (2023). Perfluoroalkyl substances (PFASs) are substrates of the renal human organic anion transporter 4 (OAT4). Arch. Toxicol..

[92] Kudo N., Katakura M., Sato Y., Kawashima Y. (2002). Sex hormone-regulated renal transport of perfluorooctanoic acid. Chem.-Biol. Interact..

[93] Dzierlenga A.L., Robinson V.G., Waidyanatha S., DeVito M.J., Eifrid M.A., Gibbs S.T., Granville C.A., Blystone C.R. (2020). Toxicokinetics of perfluorohexanoic acid (PFHxA), perfluorooctanoic acid (PFOA) and perfluorodecanoic acid (PFDA) in male and female Hsd:Sprague dawley SD rats following intravenous or gavage administration. Xenobiotica.

[94] Huang M.C., Dzierlenga A.L., Robinson V.G., Waidyanatha S., DeVito M.J., Eifrid M.A., Granville C.A., Gibbs S.T., Blystone C.R. (2019). Toxicokinetics of perfluorobutane sulfonate (PFBS), perfluorohexane-1-sulphonic acid (PFHxS), and perfluorooctane sulfonic acid (PFOS) in male and female Hsd:Sprague Dawley SD rats after intravenous and gavage administration. Toxicol. Rep..

[95] Ohmori K., Kudo N., Katayama K., Kawashima Y. (2003). Comparison of the toxicokinetics between perfluorocarboxylic acids with different carbon chain length. Toxicology.

[96] Sundström M., Chang S.-C., Noker P.E., Gorman G.S., Hart J.A., Ehresman D.J., Bergman A., Butenhoff J.L. (2012). Comparative pharmacokinetics of perfluorohexanesulfonate (PFHxS) in rats, mice, and monkeys. Reprod. Toxicol..

[97] Lehmler H.-J. (2005). Synthesis of environmentally relevant fluorinated surfactants—A review. Chemosphere.

[98] Lehmler H.-J., Rao V.V.V.N.S.R., Nauduri D., Vargo J.D., Parkin S. (2007). Synthesis and Structure of Environmentally Relevant Perfluorinated Sulfonamides. J. Fluor. Chem..

[99] Liwara D.J., Pavlov A., Johansen J.E., Leonards P.E.G., Brandsma S., de Boer J., Liu H. (2025). Synthesis of reference standards for emerging sulfonamide PFAS precursors suspected in aqueous film-forming foams via a benzyl intermediate. Tetrahedron.

[100] Qiu Z.-M. (2005). Water-and Oil-Repellency Imparting Ester Oligomers Comprising Perfluoroalkyl Moieties.

[101] Brown-Leung J.M., Cannon J.R. (2022). Neurotransmission Targets of Per- and Polyfluoroalkyl Substance Neurotoxicity: Mechanisms and Potential Implications for Adverse Neurological Outcomes. Chem. Res. Toxicol..

[102] Manojkumar Y., Pilli S., Rao P.V., Tyagi R.D. (2023). Sources, occurrence and toxic effects of emerging per- and polyfluoroalkyl substances (PFAS). Neurotoxicol. Teratol..

[103] Gaballah S., Swank A., Sobus J.R., Howey X.M., Schmid J., Catron T., McCord J., Hines E., Strynar M., Tal T. (2020). Evaluation of Developmental Toxicity, Developmental Neurotoxicity, and Tissue Dose in Zebrafish Exposed to GenX and Other PFAS. Environ. Health Perspect..

[104] Ríos-Bonilla K.M., Aga D.S., Lee J., König M., Qin W., Cristobal J.R., Atilla-Gokcumen G.E., Escher B.I. (2024). Neurotoxic Effects of Mixtures of Perfluoroalkyl Substances (PFAS) at Environmental and Human Blood Concentrations. Environ. Sci. Technol..

[105] Lagostena L., Rotondo D., Gualandris D., Calisi A., Lorusso C., Magnelli V., Dondero F. (2024). Impact of Legacy Perfluorooctane Sulfonate (PFOS) and Perfluorooctanoate (PFOA) on GABA Receptor-Mediated Currents in Neuron-Like Neuroblastoma Cells: Insights into Neurotoxic Mechanisms and Health Implications. J. Xenobiot..

[106] Bak L.K., Schousboe A., Waagepetersen H.S. (2006). The glutamate/GABA-glutamine cycle: Aspects of transport, neurotransmitter homeostasis and ammonia transfer. J. Neurochem..

[107] Kanwal S., Incharoensakdi A. (2019). The role of GAD pathway for regulation of GABA accumulation and C/N balance in *Synechocystis* sp. PCC6803. J. Appl. Phycol..

