# Supplementary material for: Non-Targeted Analysis (NTA) of Plasma and Liver from Sprague Dawley Rats Exposed to Perfluorohexanesulfonamide (PFHxSA), a Precursor to Perfluorohexane Sulfonic Acid (PFHxS)
Source: Toxics. 2025 Jun 21;13(7):523. doi: 10.3390/toxics13070523 (PMC12298034; doi:10.3390/toxics13070523)

Supporting Information

Non-targeted Analysis (NTA) of Plasma and Liver from Sprague Dawley Rats Exposed to Perfluorohexanesulfonamide (PFHxSA), a Precursor to Perfluorohexane Sulfonic Acid (PFHxS)

Denise K. MacMillan[1]*, Jackson G. Bounds[2], William. A. Willis[2], Mark J. Strynar[3], Barbara A. Wetmore[1], Richard J. Liberatore[3], James P. McCord[3], and Michael J. Devito [1]

[1]Center for Computational Toxicology and Exposure, Office of Research and Development, U.S. Environmental Protection Agency (EPA), Durham, NC 27709, USA
macmillan.denise@epa.gov (D.K.M.); wetmore.barbara@epa.gov (B.A.W.); devito.michael@epa.gov (M.J.D.)

[2] Oak Ridge Associated Universities (ORAU), Oak Ridge, TN 37830, USA; bounds.jackson@epa.gov (J.G.B)

[3] Center for Environmental Measurement and Modeling, Office of Research and Development, U. S. Environmental Protection Agency (EPA), Durham, NC, 27709, USA; strynar.mark@epa.gov (M.J.S.); liberatore.richard@epa.gov (R.J.L.); mccord.james@epa.gov (J.P.M.)

* Correspondence: macmillan.denise@epa.gov ; Tel.: +1-919-397-8038

Table of Contents

Supplementary Tables

Table S1 Liquid Chromatography Gradient

Table S2 MS Instrument Conditions

Table S3 NTA Sciex OS Data Processing Parameters

Table S4 MarkerView Data Processing Parameters

Table S5 INTERPRET NTA Data Processing Parameters

Table S6 PFHxSA Predicted Biotransformation Products

Table S7 Feature Identifications (See *Table S7.xlsx*)

Supplementary Figures

Figure S1 NTA Batch Quality Control

A: Plasma Internal Standard Peak Areas

B: Coefficient of Variation (CV) vs Abundance

C: Occurrence Heatmap, Sample Replicate Threshold 66%

D: Occurrence Heatmap, Sample Replicate Threshold 100%

Figure S2 Annotated Spectra of Select Features (See *Figure 2 Annotated Spectra.pdf*)

A: Perfluoroalkyl sulfinic acid homologues

B: Perfluoroalkyl ether sulfonamides

C: Perfluoroalkyl ether sulfonic acid homologues

D. [(Perfluoroalkyl-1-sulfonyl)amido]alkylcarboxylic acids

Figure S3 PFHxSA-N-glucuronide extracted ion chromatogram from a high dose plasma sample

Figure S4 Peak Areas for Detections in Plasma and Liver

A: Plasma (See *Fig S4A-Peak Areas for Detections in Plasma.pdf*)

B: Liver (See *Fig S4B-Peak Areas for Detections in Liver.pdf*)

Figure S5 Median percent of total peak areas for 6 co-occuring PFAS

Table S1. Liquid chromatography gradient used for NTA

Time (min)	%A	%B
0.01	90	10
1	90	10
6	45	55
16	0	100
18	0	100
18.01	90	10
20	90	10

A: (95:5 H_2O:Methanol + 10 mM Ammonium acetate)
B: (95:5 methanol:H_2O + 10mM Ammonium acetate)

Table S2. Sciex X500R instrument parameters for NTA

Sciex x500R Parameter	Information Dependent Analysis NTA	Information Independent Analysis NTA
Source	ESI	ESI
Polarity	Negative	Negative
Scan Type	IDA	SWATH
Ion Source Gas 1 (psi)	60	60
Ion Source Gas 2 (psi)	60	60
Curtain Gas (psi)	35	35
CAD Gas	9	9
Source Temperature (°C)	400	400
Spray Voltage (V)	-4500	-4500
TOF MS Scan Range (Da)	100-2000	100-1000
TOF MS DP (V)	-50	-80
TOF MS DP Spread (V)	10	0
TOF MS CE (V)	-10	-10
TOF MS CE Spread (V)	0	0
TOF MS Accumulation Time (sec)	0.25	0.20
TOF MSMS Scan Range (Da)	50-1000	50-1000
TOF MSMS Accumulation Time (sec)	0.1	0.03
TOF MSMS DP (V)	-50	-80
TOF MSMS DP Spread (V)	10	0
TOF MSMS CE (V)	-30	-35
TOF MSMS CE Spread (V)	10	15
Maximum Candidate Ions	15	N/A
Minimum Intensity Threshold (CPS)	250	N/A

Table S3. Sciex OS data processing parameters

Parameter	Value
Workflow	Non-Targeted Screening
Integration Algorithm	MQ4
Library Searching Algorithm	Candidate Search
Library	In House PFAS; Sciex Fluorochemical
Precursor Mass Tolerance, Da	0.4
Fragment Mass Tolerance, Da	0.4
Formula Finder Mass Tolerance, ppm	5
Formula Finder Compound Type	Man-Made
Peak Detection Minimum Retention Time	1.0
Peak Detection Sensitivity	Exhaustive
Area Ratio Threshold (Unknown/Control)	0
Group Peaks by Adduct or Charge	Yes

Table S4. Sciex MarkerView data processing parameters – NTA

Process	Parameter	Value
Peak Peaking	Period	1
	Experiment	TOFMS
	Minimum Retention Time, min	1.00
	LC Peak Width	8 sec
	Minimum Intensity	10 counts
	Subtraction Offset, scans	10
	Subtraction Multiplication Factor	1.3
	Noise Threshold	2
	Minimum Spectral Peak Width, Da	0.01
	Minimum Retention Time Peak Width, scans	3
Alignment and Filtering	Perform Background Subtraction	Yes
	Chemical Noise Intensity Multiplier	2
	Maximum Number of Peaks	10000
	Intensity Threshold	2
	Isotope Filtering	No
	Retention Time Correction	No
	RT Tolerance, min	0.5
	Mass Tolerance	10 ppm
	Use Global Exclusion List	False
	Num Required Samples	2
	Use Raw Data Area	Yes

Table S5. INTERPRET NTA data processing parameters – NTA

Note: The table below indicates settings used, not all possible settings.

t	Value
**Negative mode adducts:**	$[M+HCO_2]$-
**Neutral losses & solvent modifiers (both modes):**	$[M-H_2O]$<br>$[M-CO_2]$
**Adduct / duplicate mass accuracy units:**	ppm
**Adduct / duplicate mass accuracy:**	5 ppm
**Tracer mass accuracy units:**	ppm
**Tracer mass accuracy:**	5 ppm
**Tracer plot y-axis scaling:**	log
**Tracer plot trendlines shown:**	yes
**Min replicate hits (%):**	66
**Min replicate hits in blanks (%):**	66
**Max replicate CV:**	0.8

t	Value
**MRL standard deviation multiplier:**	3
**Parent ion mass accuracy (ppm):**	10
**Discard features below this retention time (mins):**	2
**Search DSSTox for possible structures:**	yes
**Search Cheminformatics Hazard Module for toxicity data:**	yes
**Search DSSTox by:**	mass
**Run qNTA:**	no

Table S6 Chemical Transformation Simulator + Biotransformer Predicted Biotransformation Products

Predicted Biotransformation Product	Formula	Monoisotopic Mass, Da	$(M-H)^-$, m/z	CASRN	DTXSID	Biotransformation Source	Product Reaction
PFHxS[a]	$C_6HF_{13}O_3S$	399.94389	398.9417	355-46-4	7040150	PFAS Environmental; PFAS Metabolism	Hydrolysis: Sulfonamide to sulfonic acid
PFHxSi[b]	$C_6HF_{13}O_2S$	383.94897	382.9417	115416-67-6	601032911	PFAS Environmental	Reduction: Sulfonamide to sulfinic acid
PFHxSA-N-glucuronide[c]	$C_{12}H_{10}F_{13}NO_8S$	574.9920	573.9849	N/A	N/A	Mammalian Metabolism - Phase II	N-Glucuronidation of sulfonamide
N-hydroxy-PFHxSA[d]	$C_6H_2F_{13}NO_3S$	414.9547	413.9475	N/A	N/A	Mammalian Metabolism	N-hydroxylation

a: Perfluorohexanesulfonic acid

b: Perfluorohexanesulfinic acid

c: (N-(6-Carboxy-3,4,5-trihydroxyoxan-2-yl)1,1,2,2,3,3,4,4,5,5,6,6,6-tridecafluorohexanesulfonamido)

d: (1,1,2,2,3,3,4,4,5,5,6,6,6-Tridecafluoro-N-hydroxyhexane-1-sulfonamide)

Figure S1-A: NTA Batch Quality Control: Plasma Internal Standard Peak Areas

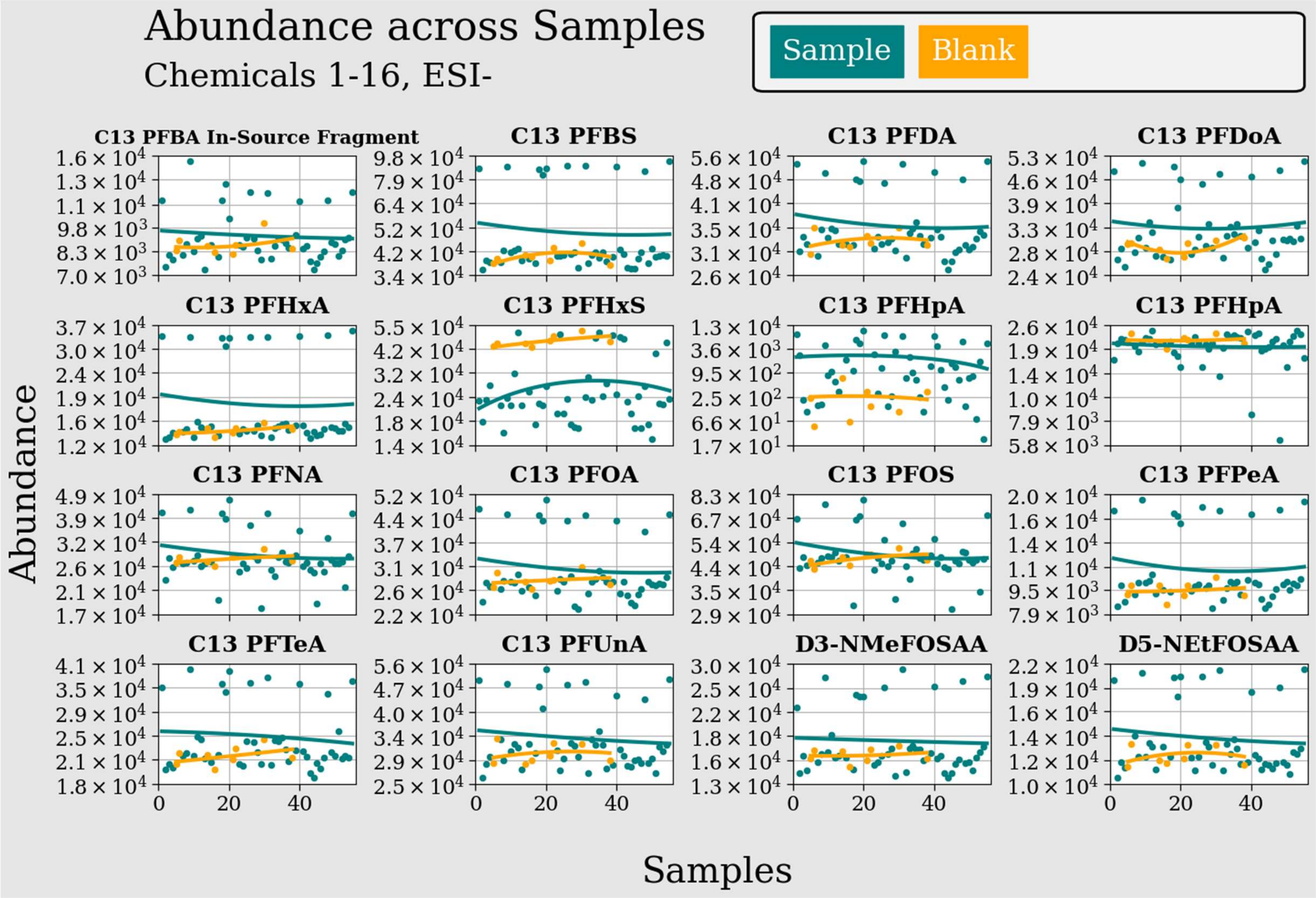

Figure S1-B: NTA Batch Quality Control: Coefficient of Variation (CV) vs Abundance of Blanks and Non-Blanks

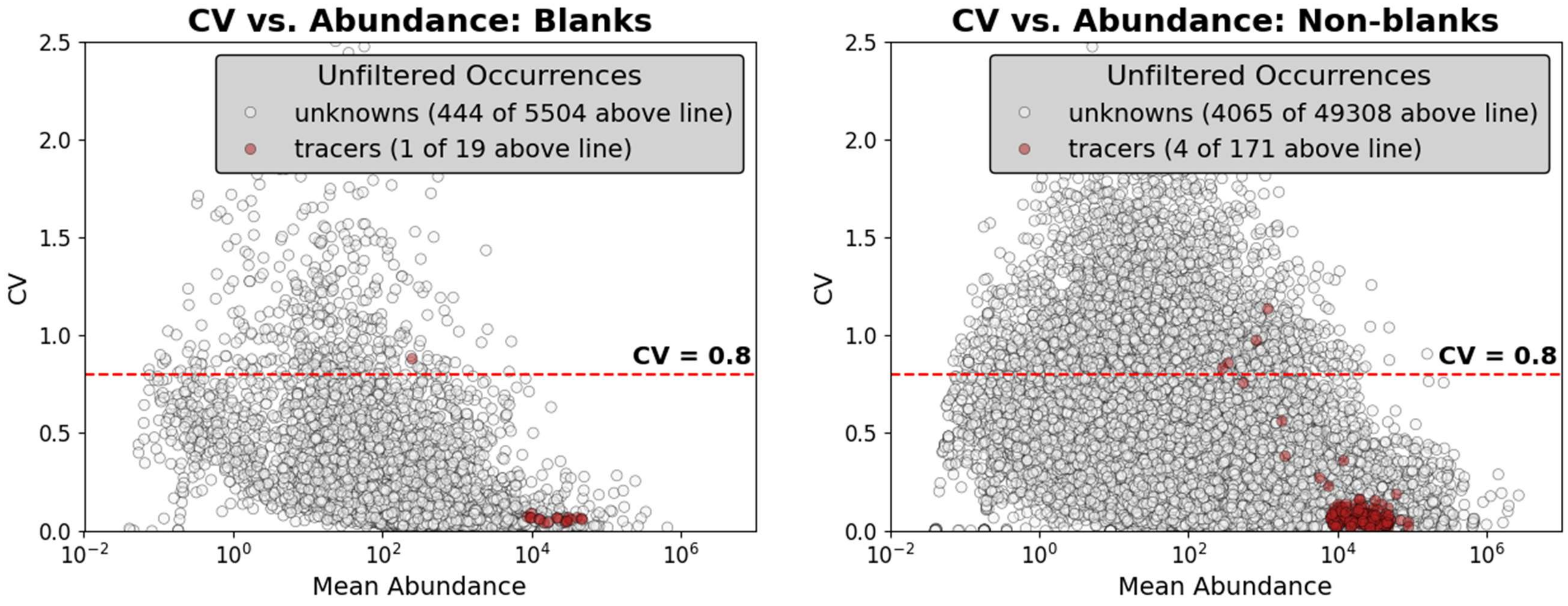

Figure S1-C: NTA Batch Quality Control: Occurrence Heatmap, Sample Replicate (Rep) Threshold 66%

MRL: Minimum Reporting Limit
F100: Female Rat Plasma, 100 mg/kg/day Dose Group
F1: Female Rat Plasma, 1 mg/kg/day Dose Group
M100: Male rat Plasma, 100 mg/kg/day Dose Group
M1: Male rat Plasma, 1 mg/kg/day Dose Group
MV: Male Rat Plasma, Vehicle Control
Blank: Mobile Phase Blank
F10: Female Rat Plasma, 10 mg/kg/day Dose Group
F30: Female Rat Plasma: 30 mg/kg/day Dose Group
M10: Male Rat Plasma, 10 mg/kg/day Dose Group
M30: Male Rat Plasma, 30 mg/kg/day Dose Group

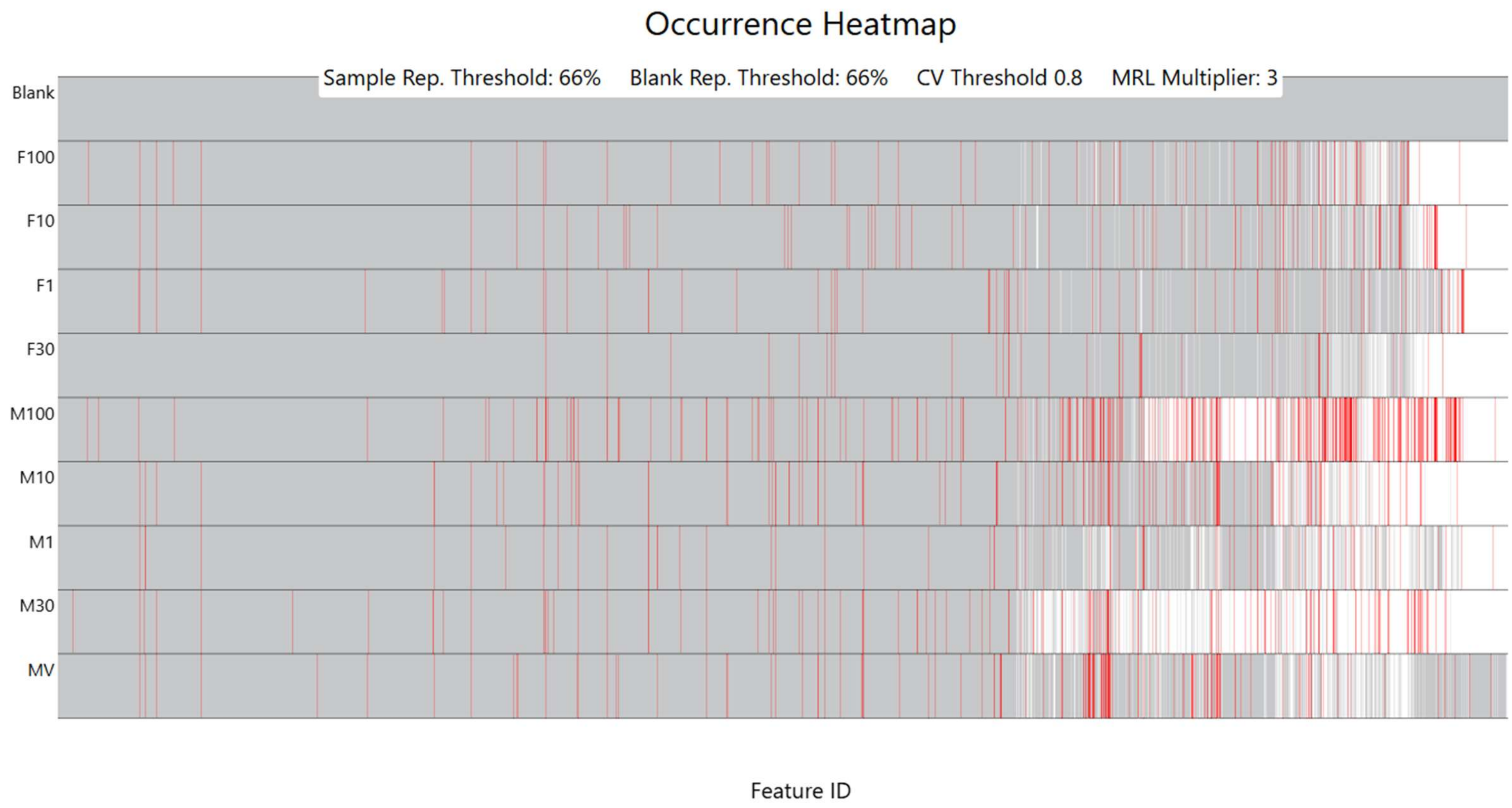

Figure S1-C: NTA Batch Quality Control: Occurrence Heatmap, Sample Replicate (Rep) Threshold 100%

MRL: Minimum Reporting Limit
F100: Female Rat Plasma, 100 mg/kg/day Dose Group
F1: Female Rat Plasma, 1 mg/kg/day Dose Group
M100: Male rat Plasma, 100 mg/kg/day Dose Group
M1: Male rat Plasma, 1 mg/kg/day Dose Group
MV: Male Rat Plasma, Vehicle Control
Blank: Mobile Phase Blank
F10: Female Rat Plasma, 10 mg/kg/day Dose Group
F30: Female Rat Plasma: 30 mg/kg/day Dose Group
M10: Male Rat Plasma, 10 mg/kg/day Dose Group
M30: Male Rat Plasma, 30 mg/kg/day Dose Group

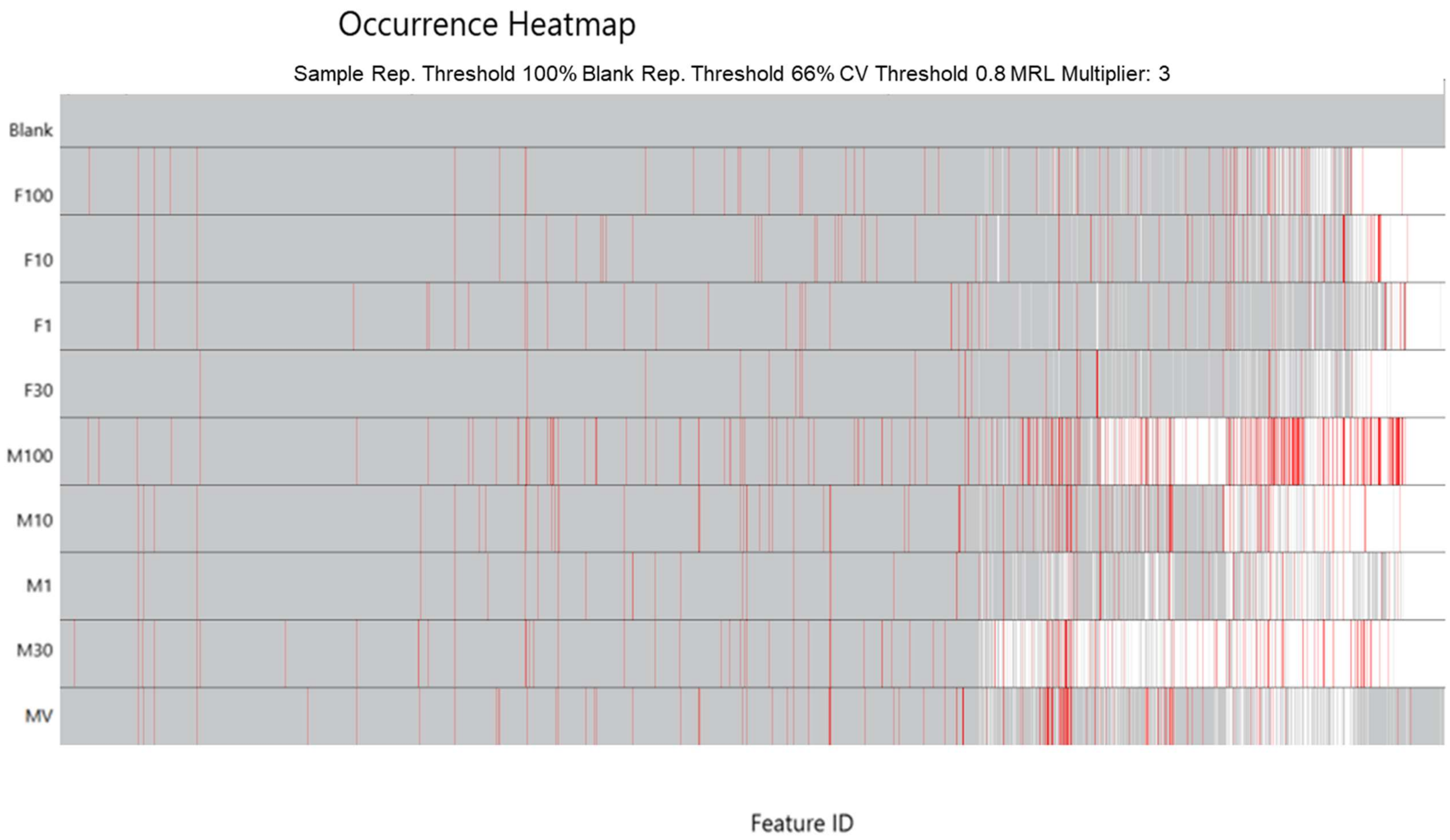

Figure S2 Annotated Spectra of Select Features (See *Figure 2 Annotated Spectra.pdf)*

Figure S3 PFHxSA-N-glucuronide extracted ion chromatogram from a high dose plasma sample

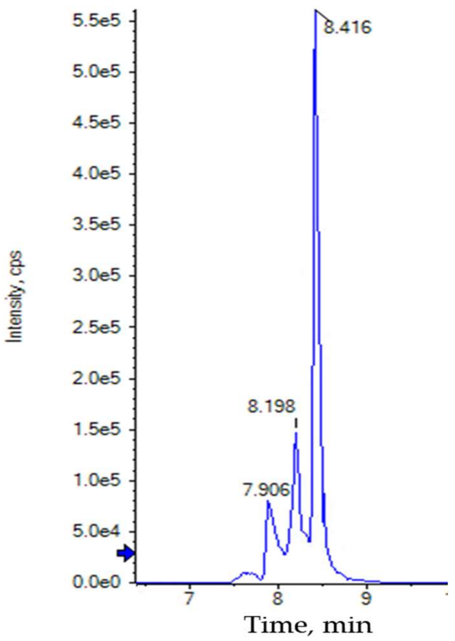

Figure S4 Peak Areas for Detections

A: Plasma (See *Figure S4-A-Peak Areas for Detections in Plasma.pdf*)

B: Liver (See *Figure S4-B-Peak Areas for Detections in Liver.pdf*)

Figure S5 Median percent of total peak areas for 6 co-occuring PFAS in Plasma

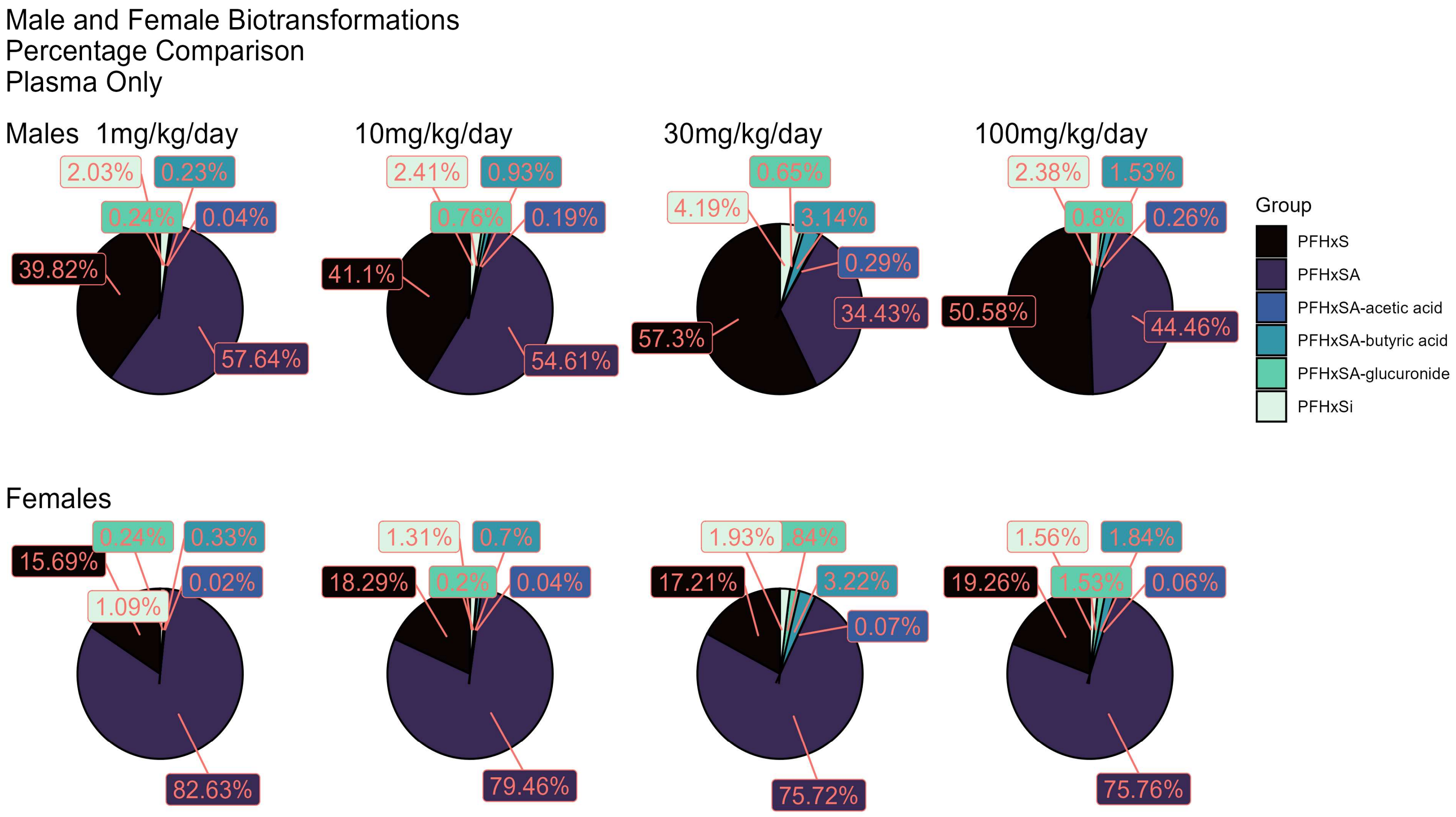

Supplement: Supplementary file 1 [file toxics-13-00523-s001.zip › toxics-3644925- SI Text and Tables-Final-proofed.pdf]
