# Supplementary figures and images for: Non-Targeted Analysis (NTA) of Plasma and Liver from Sprague Dawley Rats Exposed to Perfluorohexanesulfonamide (PFHxSA), a Precursor to Perfluorohexane Sulfonic Acid (PFHxS)
Source: Toxics. 2025 Jun 21;13(7):523. doi: 10.3390/toxics13070523 (PMC12298034; doi:10.3390/toxics13070523)

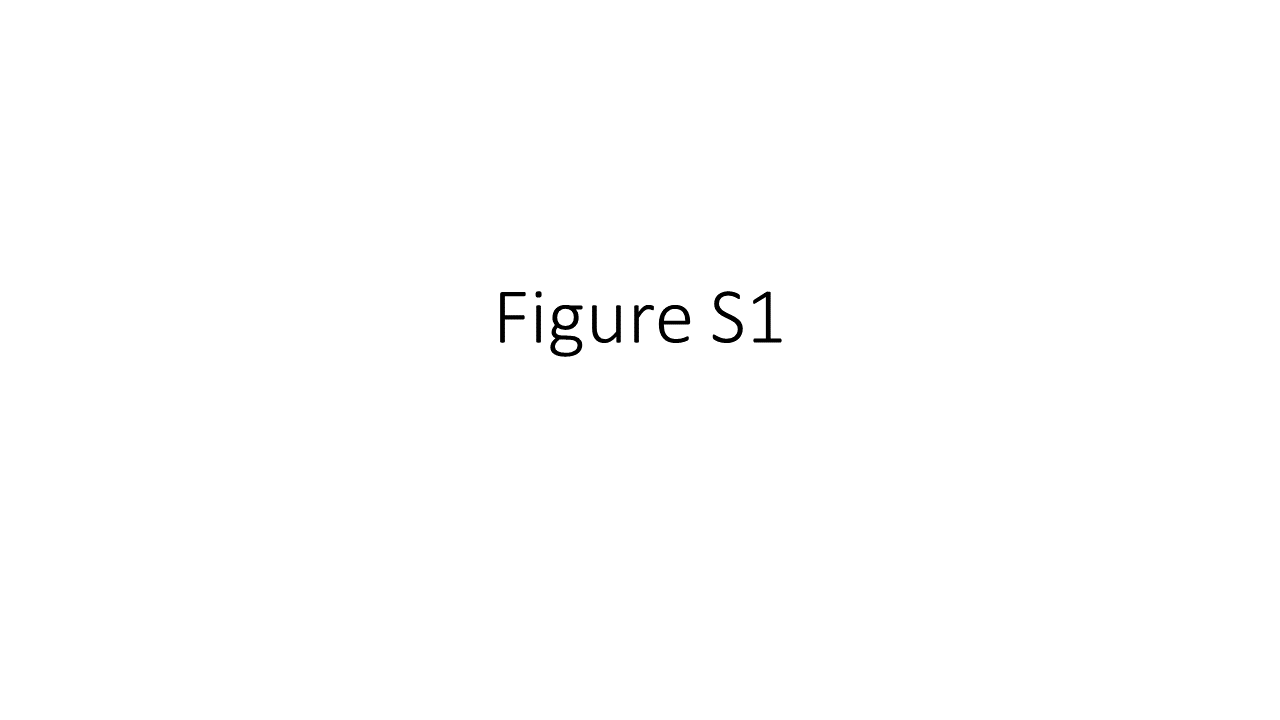

Supplement: Supplementary file 1 [file toxics-13-00523-s001.zip › Fig S1 -NTA Batch Quality Control/Slide1.PNG]

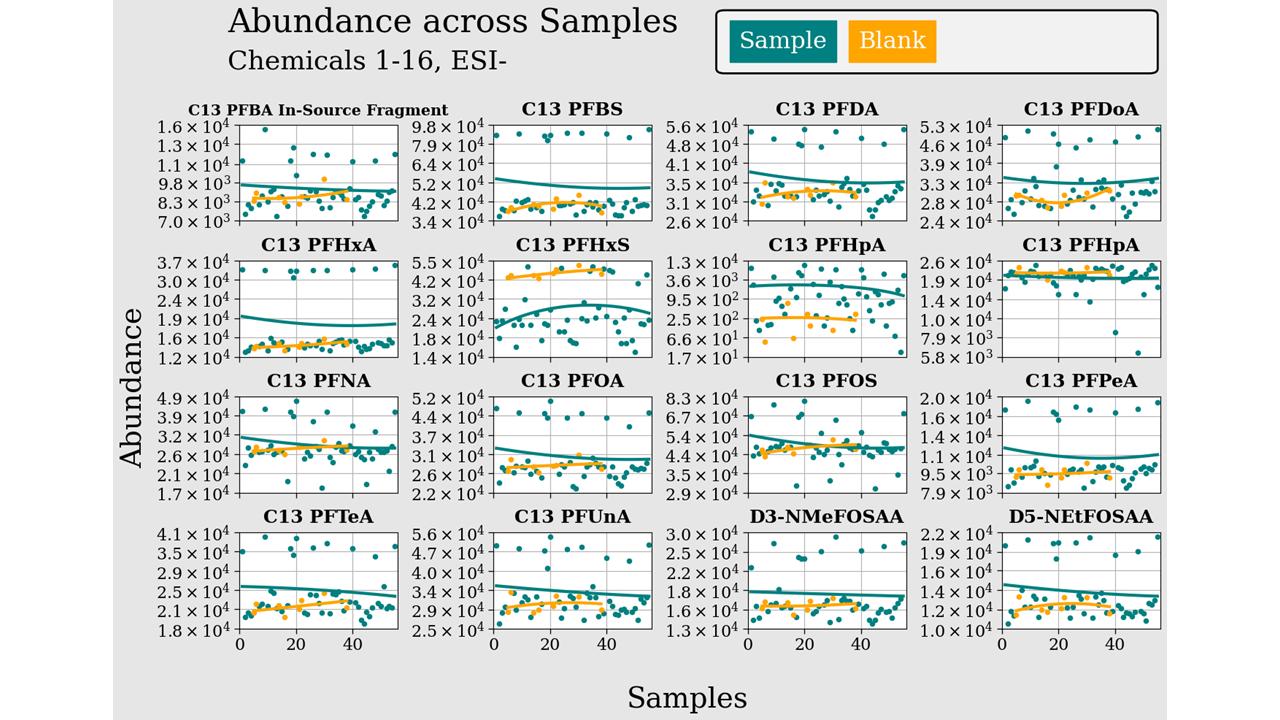

Supplement: Supplementary file 1 [file toxics-13-00523-s001.zip › Fig S1 -NTA Batch Quality Control/Slide2.PNG]

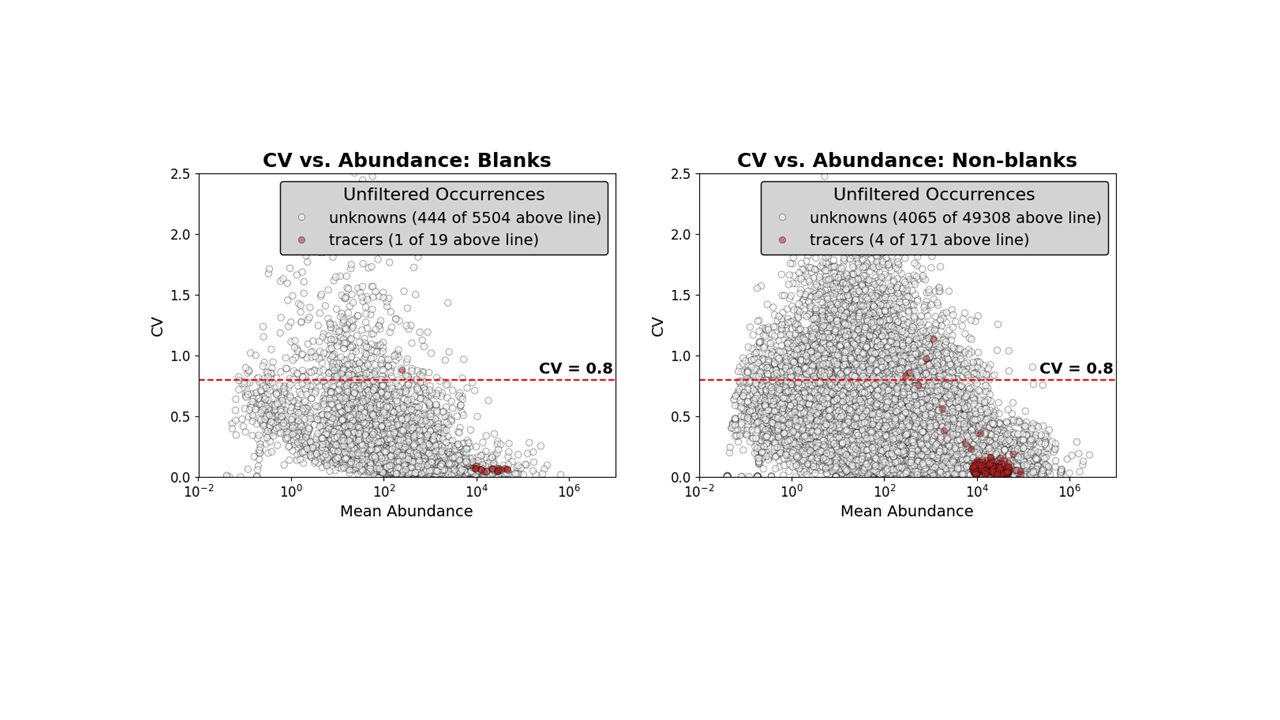

Supplement: Supplementary file 1 [file toxics-13-00523-s001.zip › Fig S1 -NTA Batch Quality Control/Slide3.PNG]

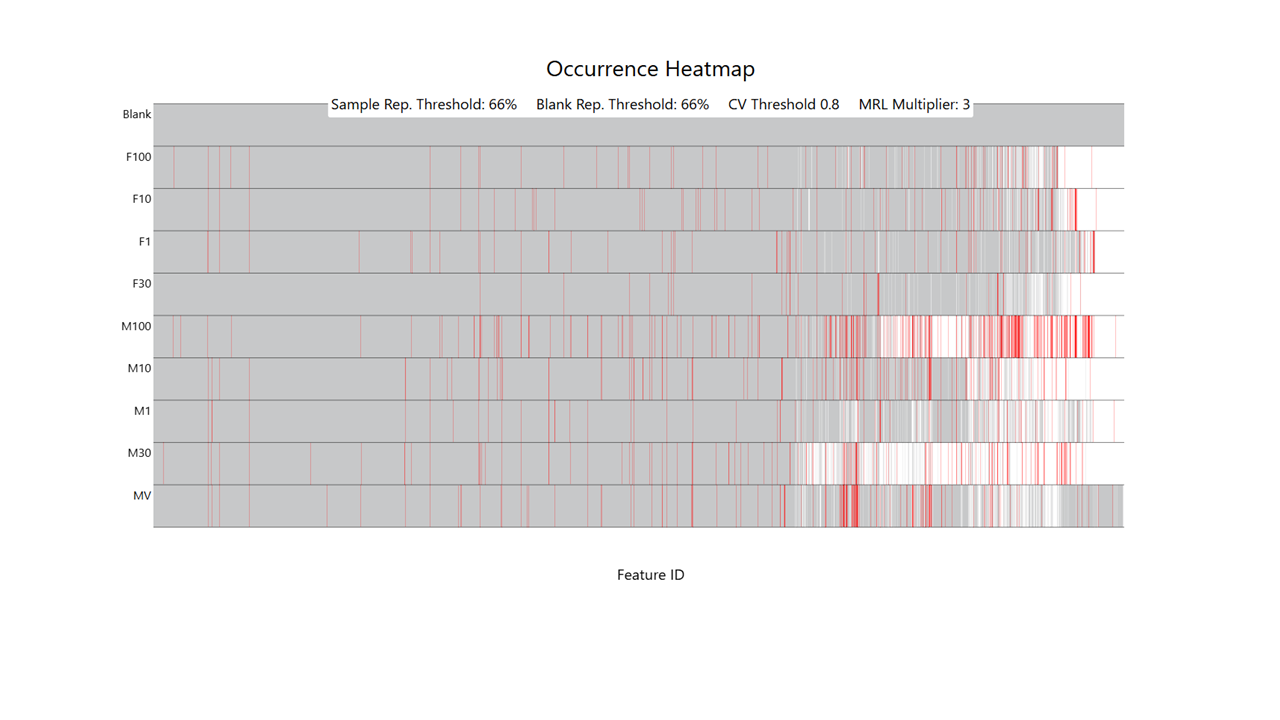

Supplement: Supplementary file 1 [file toxics-13-00523-s001.zip › Fig S1 -NTA Batch Quality Control/Slide4.PNG]

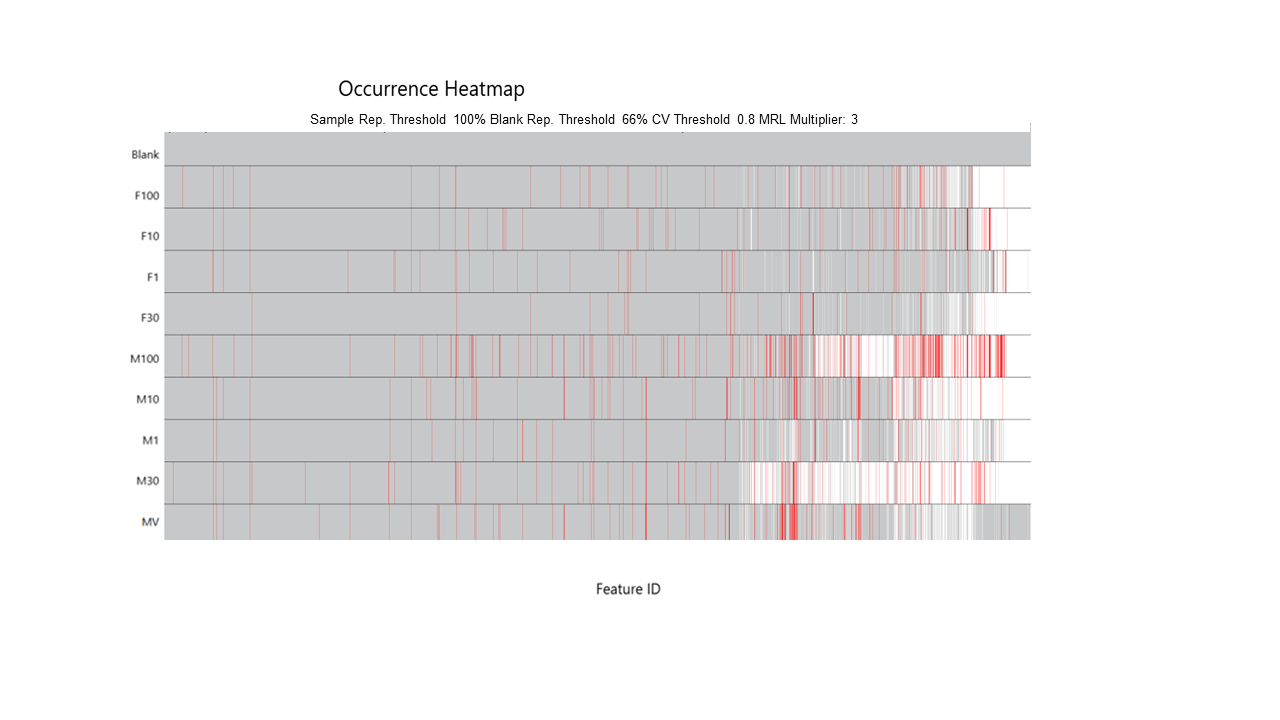

Supplement: Supplementary file 1 [file toxics-13-00523-s001.zip › Fig S1 -NTA Batch Quality Control/Slide5.PNG]

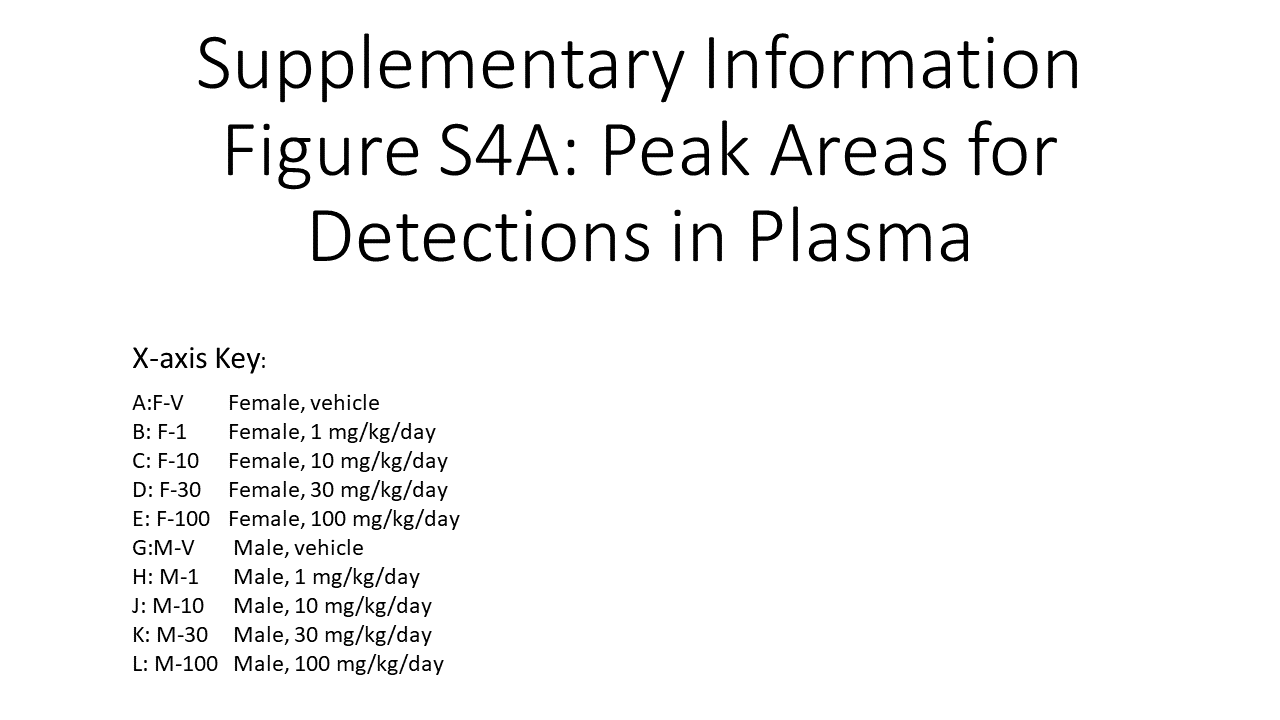

Supplement: Supplementary file 1 [file toxics-13-00523-s001.zip › Fig S4-A-Peak Areas for Detections in Plasma-rev1/Slide1.PNG]

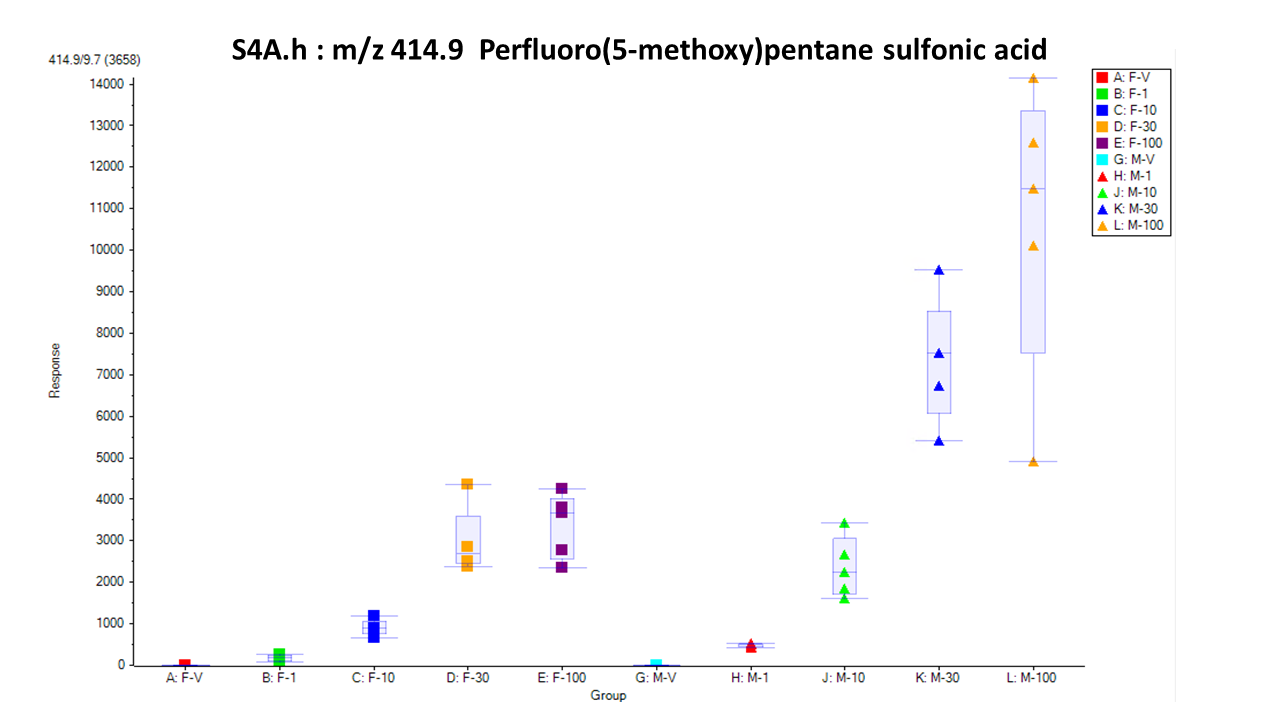

Supplement: Supplementary file 1 [file toxics-13-00523-s001.zip › Fig S4-A-Peak Areas for Detections in Plasma-rev1/Slide10.PNG]

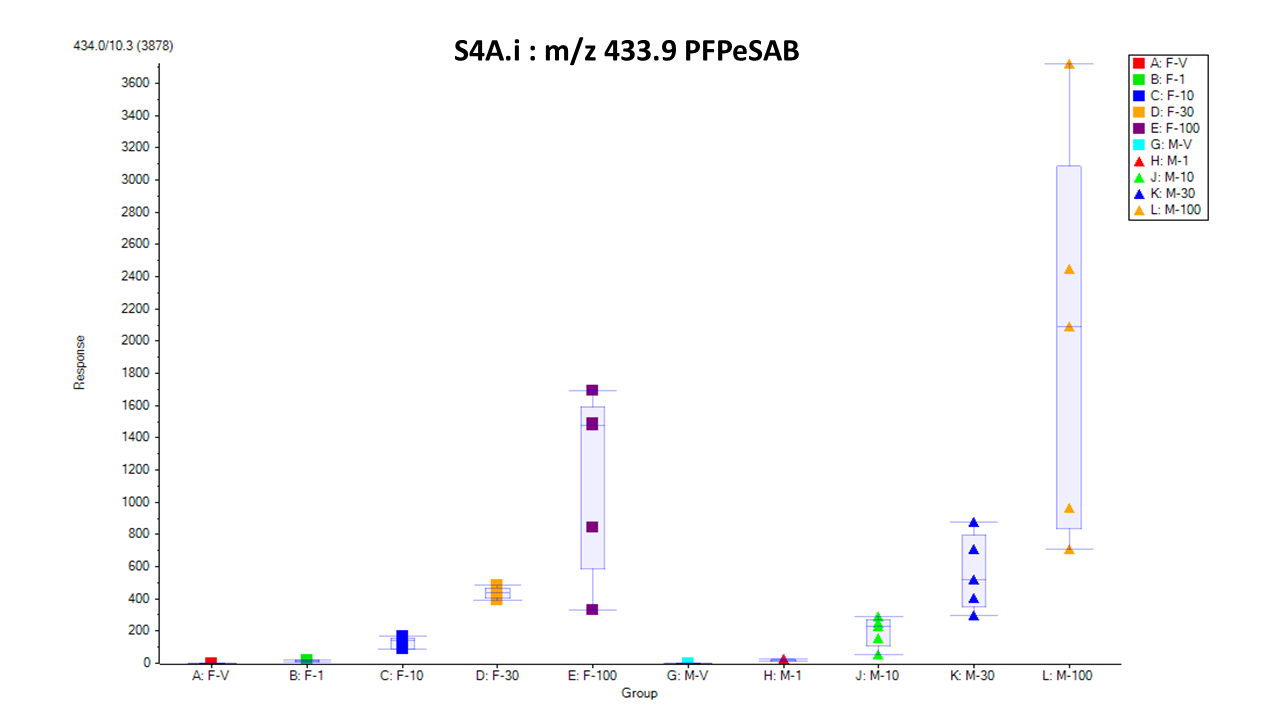

Supplement: Supplementary file 1 [file toxics-13-00523-s001.zip › Fig S4-A-Peak Areas for Detections in Plasma-rev1/Slide11.PNG]

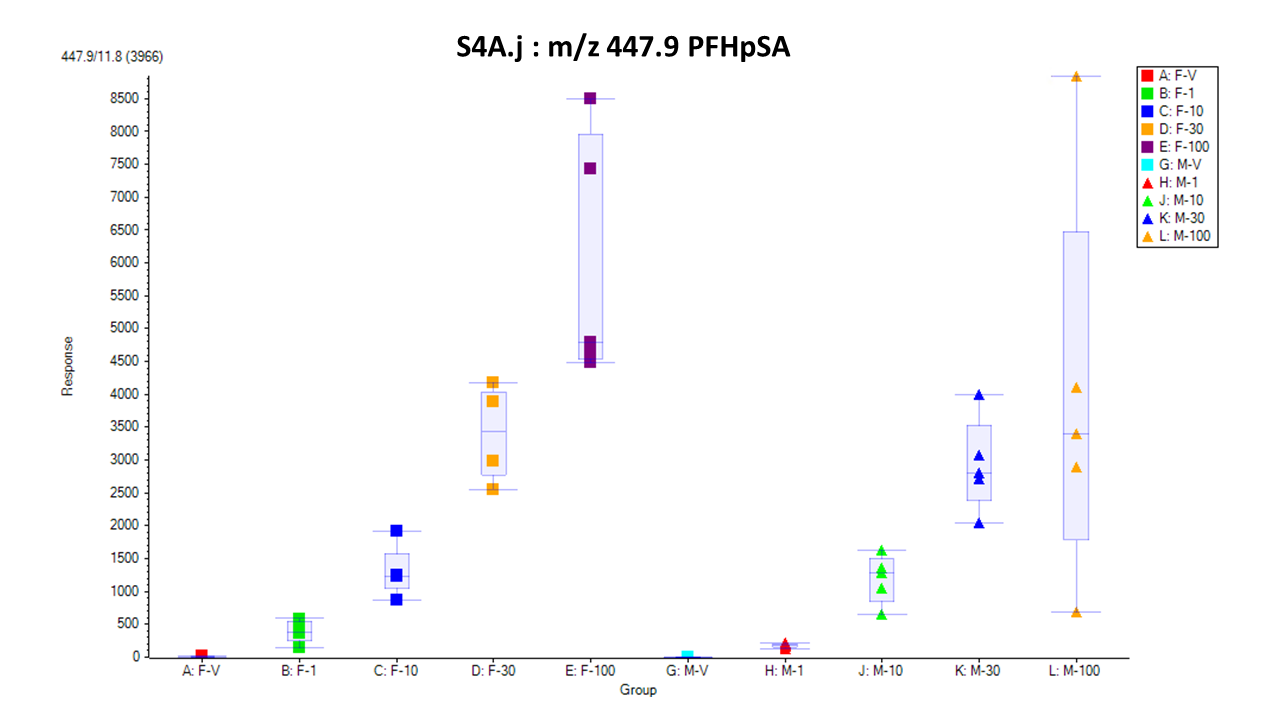

Supplement: Supplementary file 1 [file toxics-13-00523-s001.zip › Fig S4-A-Peak Areas for Detections in Plasma-rev1/Slide12.PNG]

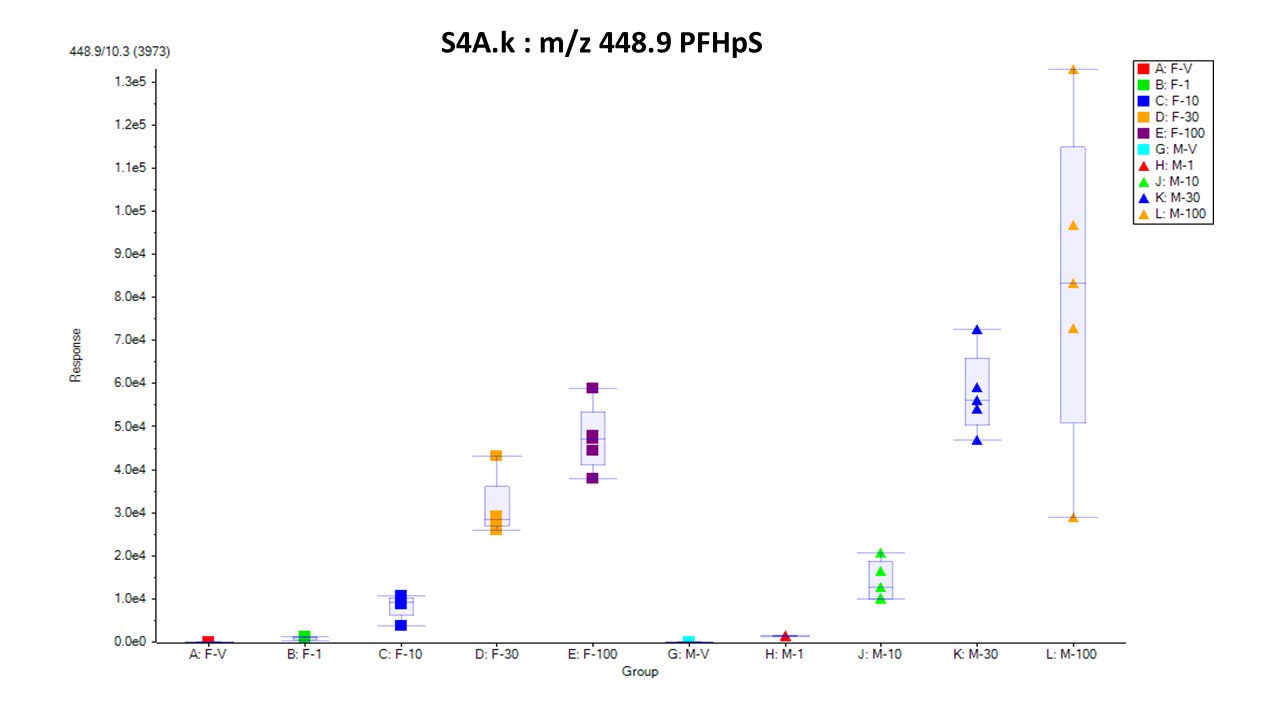

Supplement: Supplementary file 1 [file toxics-13-00523-s001.zip › Fig S4-A-Peak Areas for Detections in Plasma-rev1/Slide13.PNG]

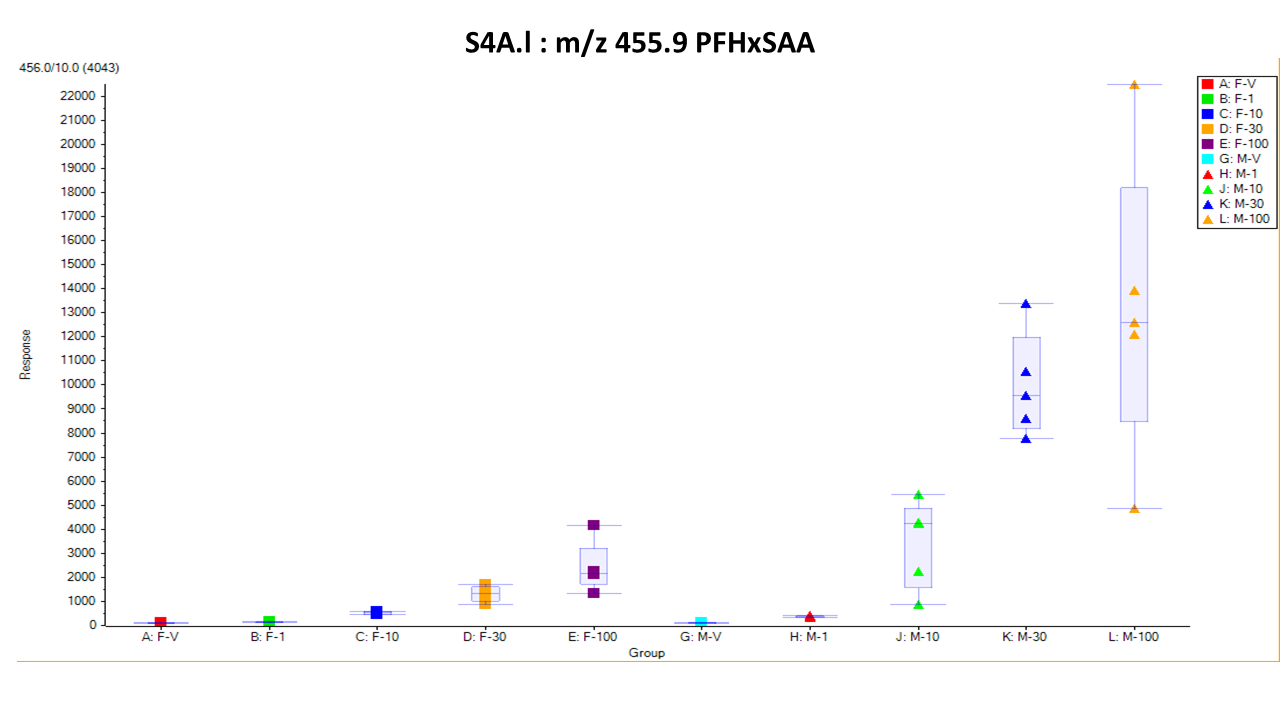

Supplement: Supplementary file 1 [file toxics-13-00523-s001.zip › Fig S4-A-Peak Areas for Detections in Plasma-rev1/Slide14.PNG]

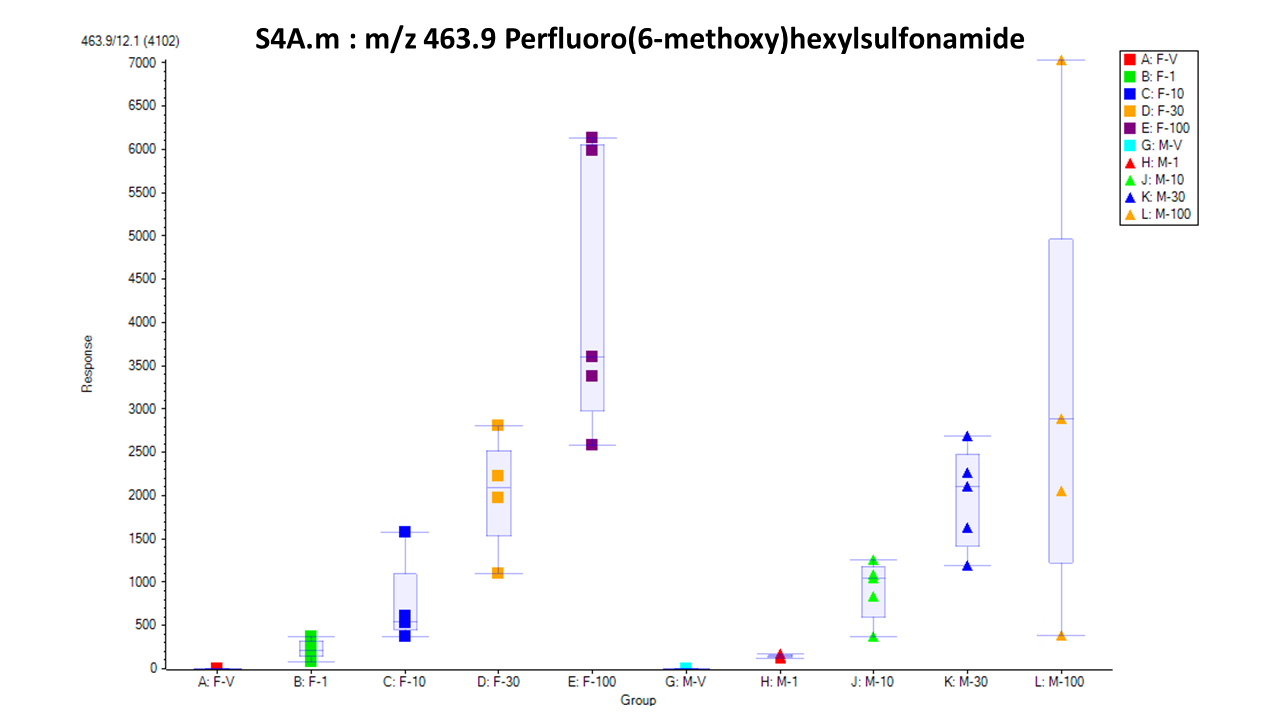

Supplement: Supplementary file 1 [file toxics-13-00523-s001.zip › Fig S4-A-Peak Areas for Detections in Plasma-rev1/Slide15.PNG]

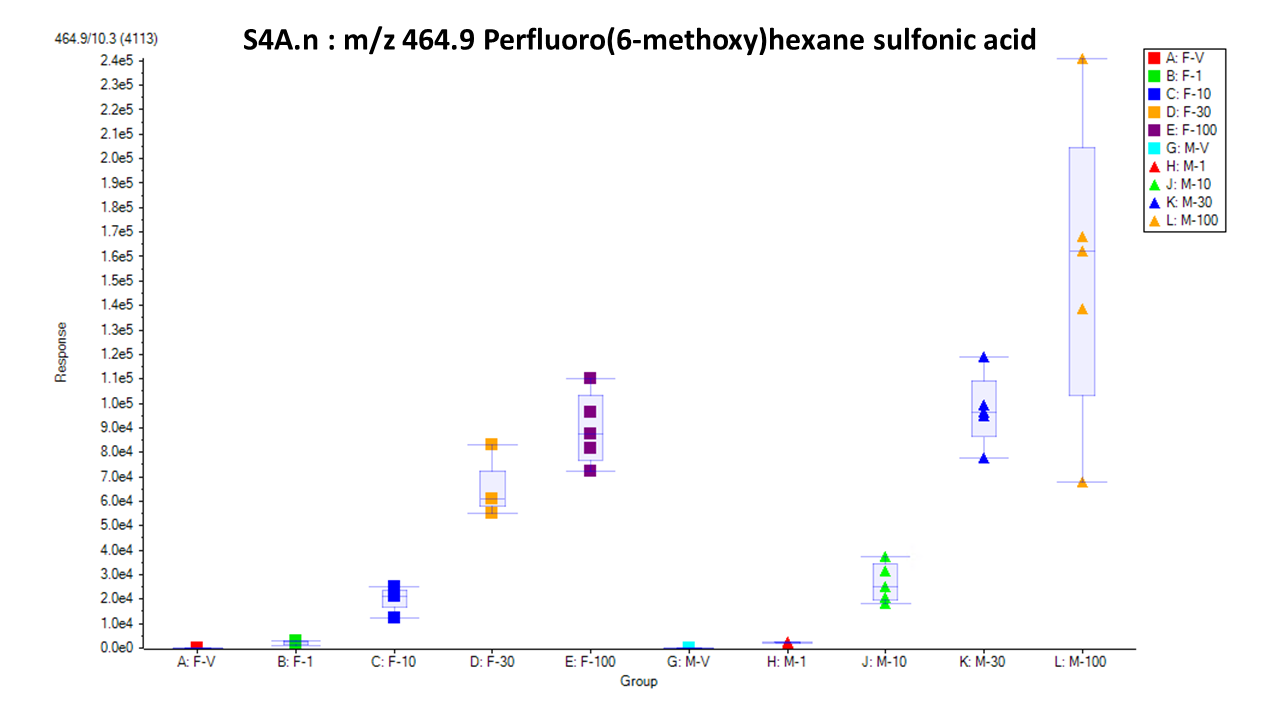

Supplement: Supplementary file 1 [file toxics-13-00523-s001.zip › Fig S4-A-Peak Areas for Detections in Plasma-rev1/Slide16.PNG]

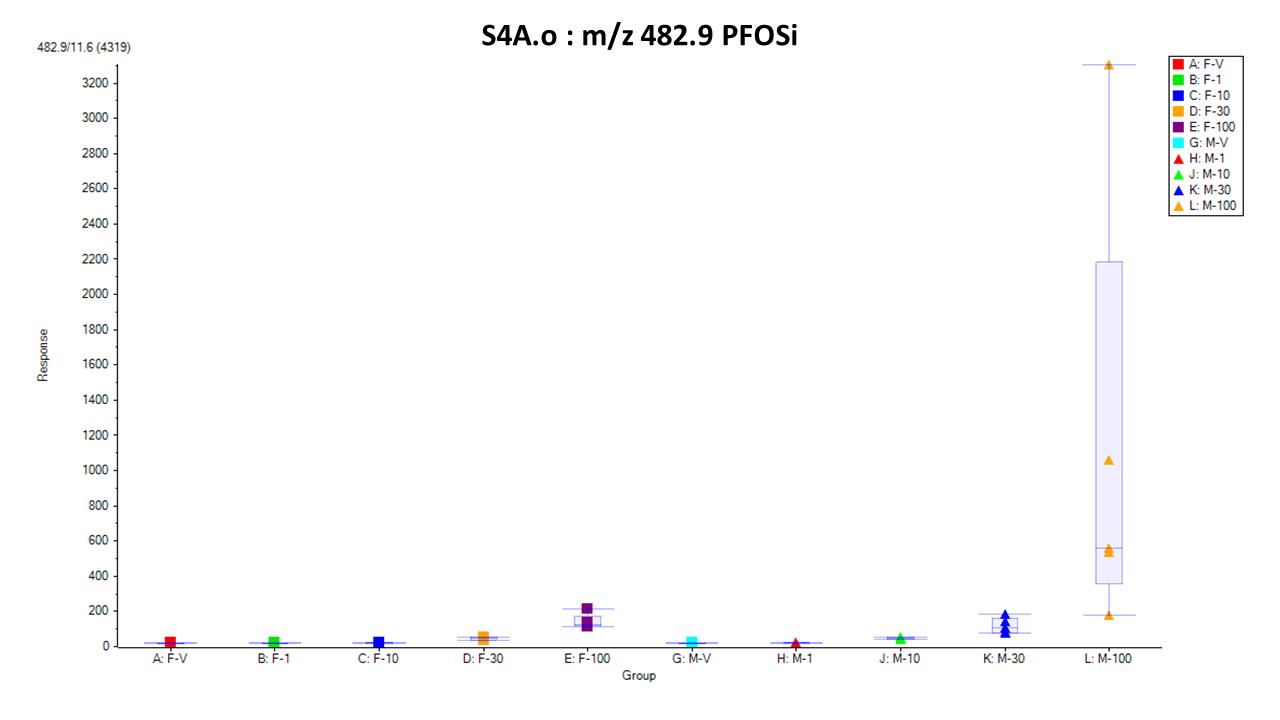

Supplement: Supplementary file 1 [file toxics-13-00523-s001.zip › Fig S4-A-Peak Areas for Detections in Plasma-rev1/Slide17.PNG]

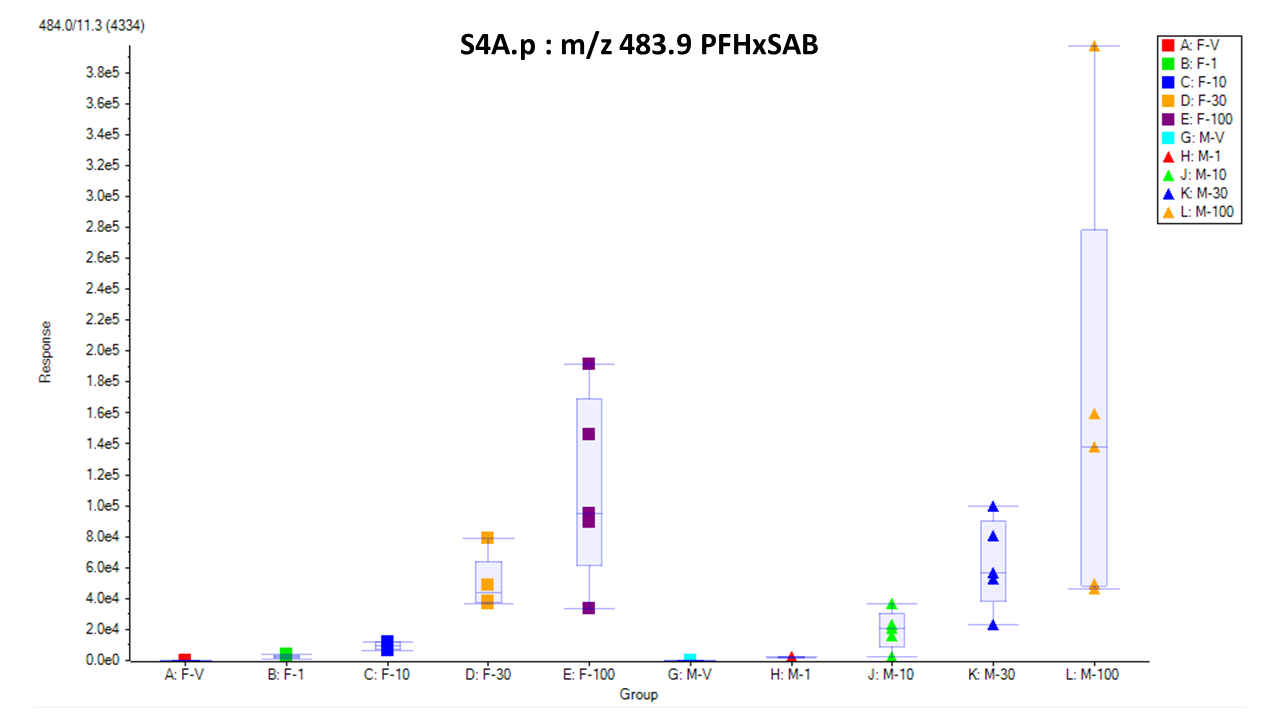

Supplement: Supplementary file 1 [file toxics-13-00523-s001.zip › Fig S4-A-Peak Areas for Detections in Plasma-rev1/Slide18.PNG]

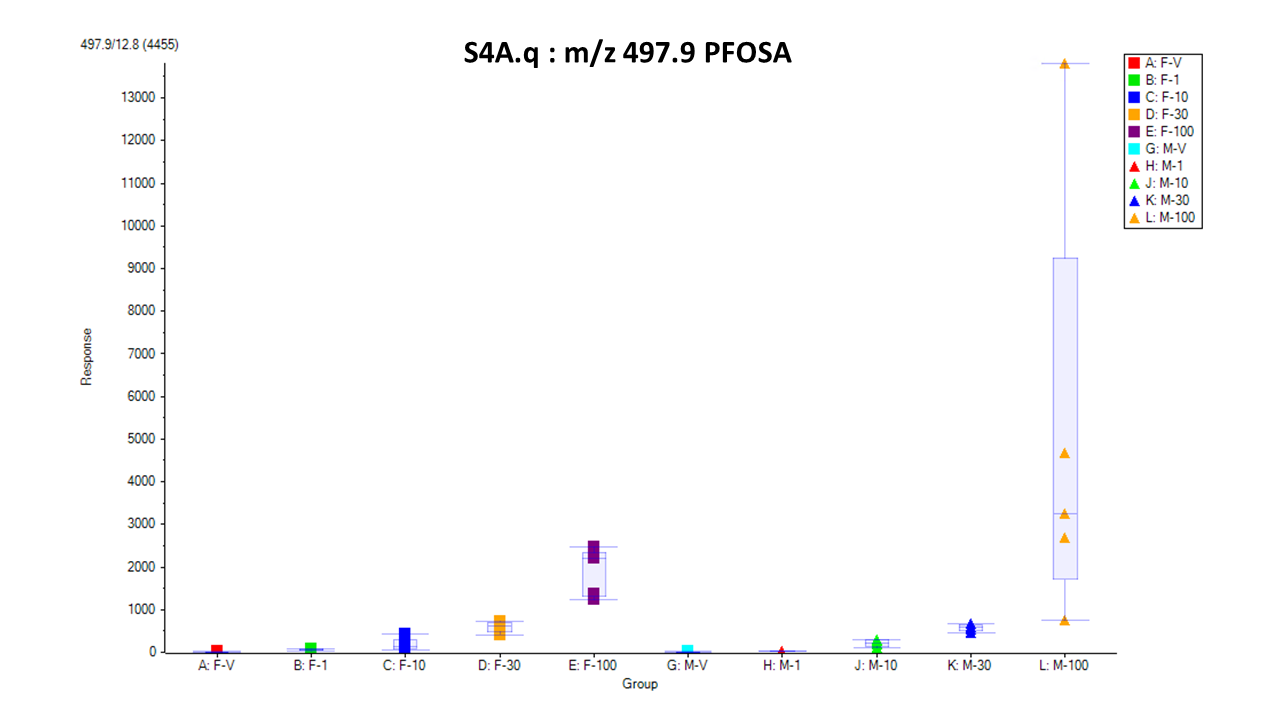

Supplement: Supplementary file 1 [file toxics-13-00523-s001.zip › Fig S4-A-Peak Areas for Detections in Plasma-rev1/Slide19.PNG]

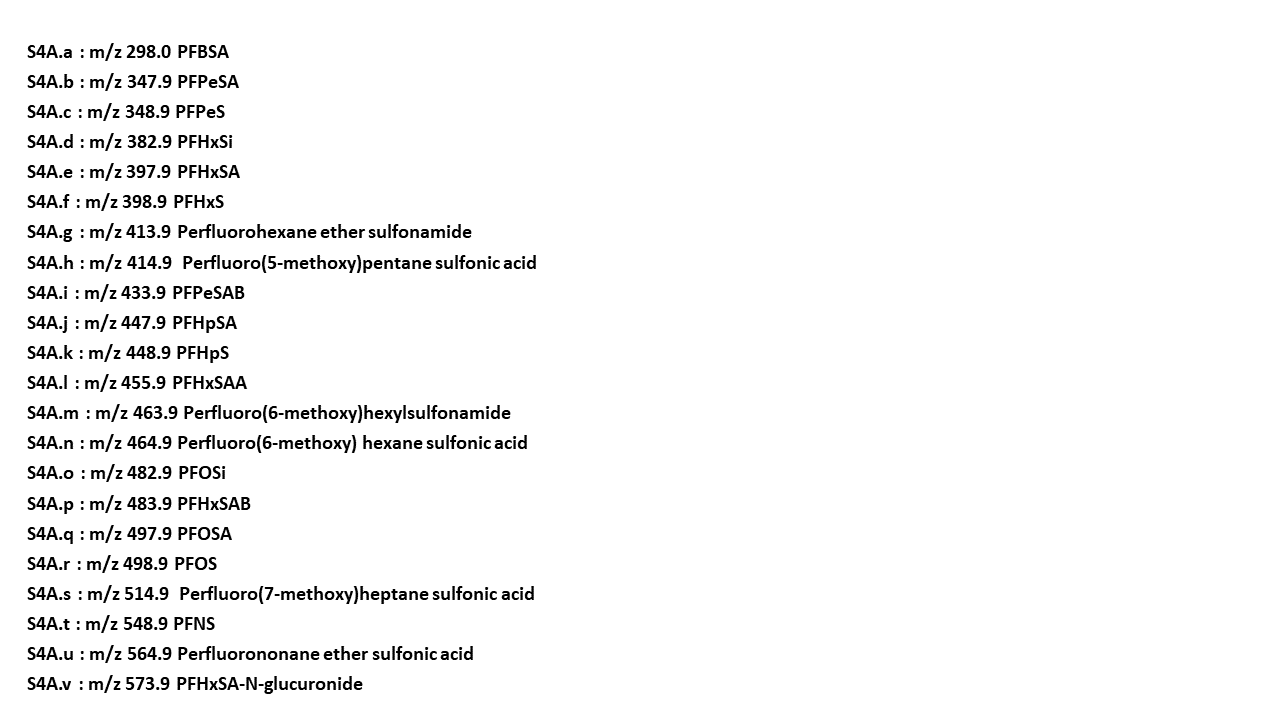

Supplement: Supplementary file 1 [file toxics-13-00523-s001.zip › Fig S4-A-Peak Areas for Detections in Plasma-rev1/Slide2.PNG]

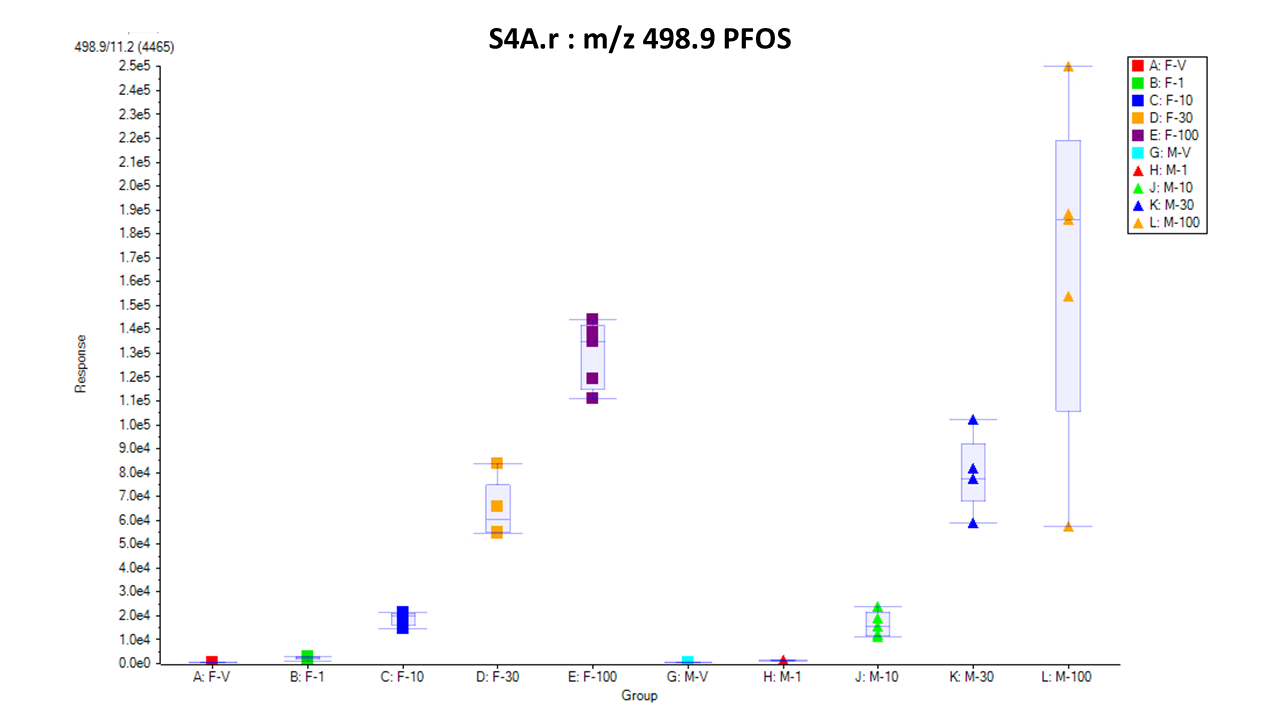

Supplement: Supplementary file 1 [file toxics-13-00523-s001.zip › Fig S4-A-Peak Areas for Detections in Plasma-rev1/Slide20.PNG]

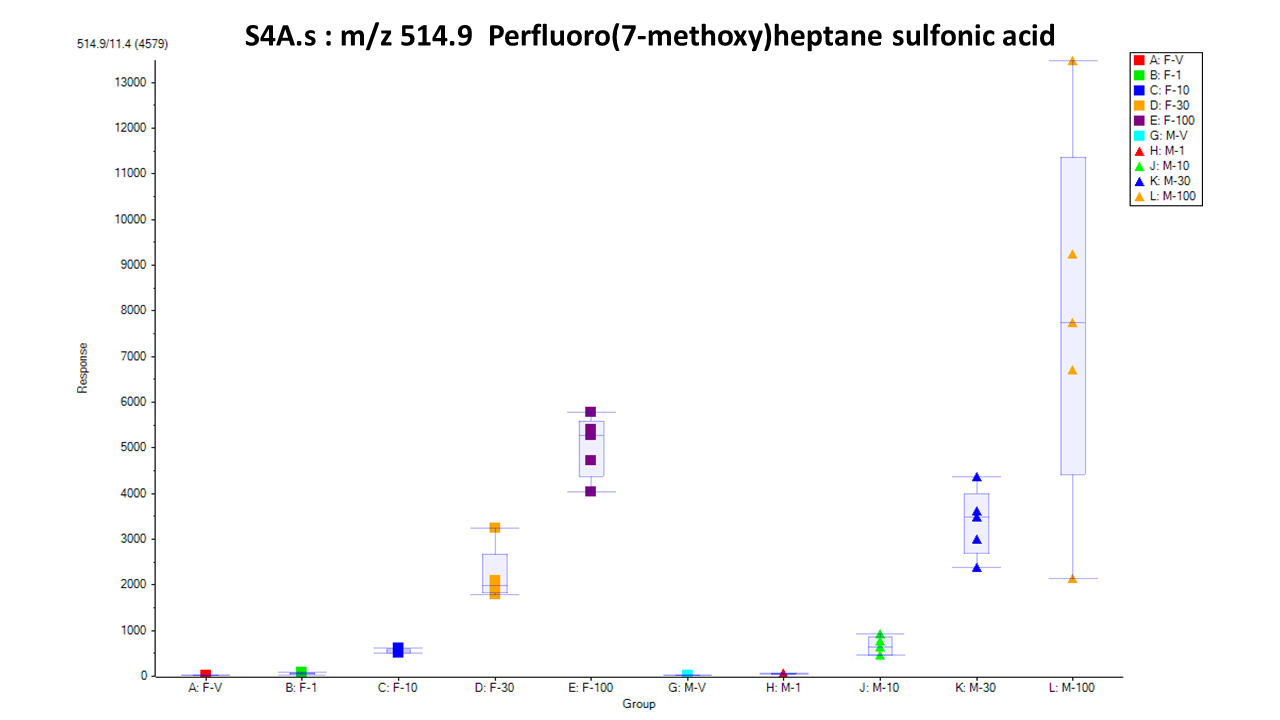

Supplement: Supplementary file 1 [file toxics-13-00523-s001.zip › Fig S4-A-Peak Areas for Detections in Plasma-rev1/Slide21.PNG]

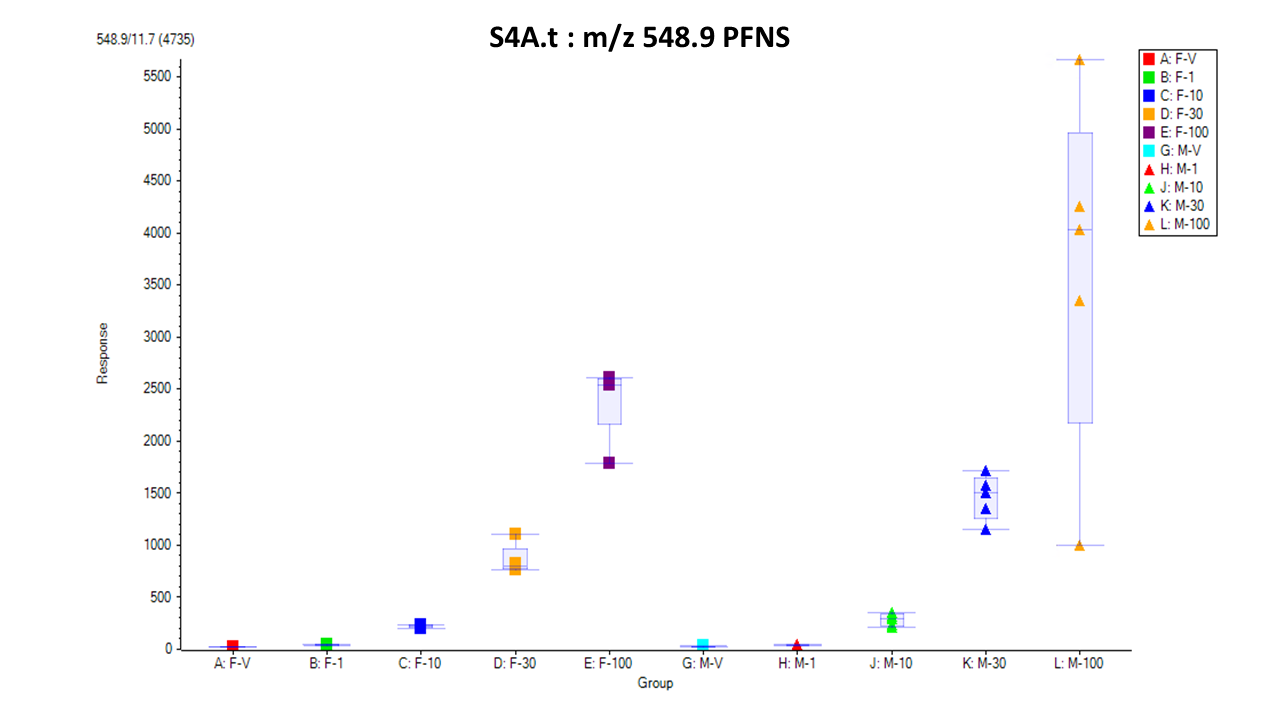

Supplement: Supplementary file 1 [file toxics-13-00523-s001.zip › Fig S4-A-Peak Areas for Detections in Plasma-rev1/Slide22.PNG]

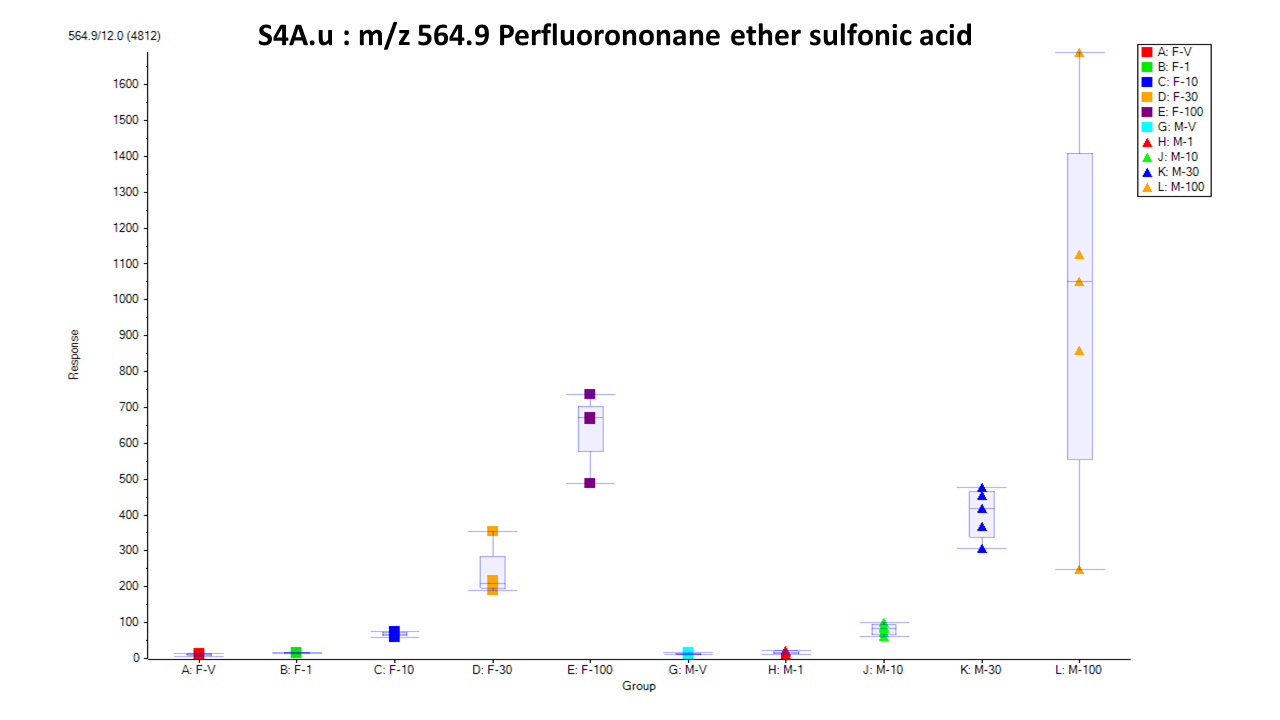

Supplement: Supplementary file 1 [file toxics-13-00523-s001.zip › Fig S4-A-Peak Areas for Detections in Plasma-rev1/Slide23.PNG]

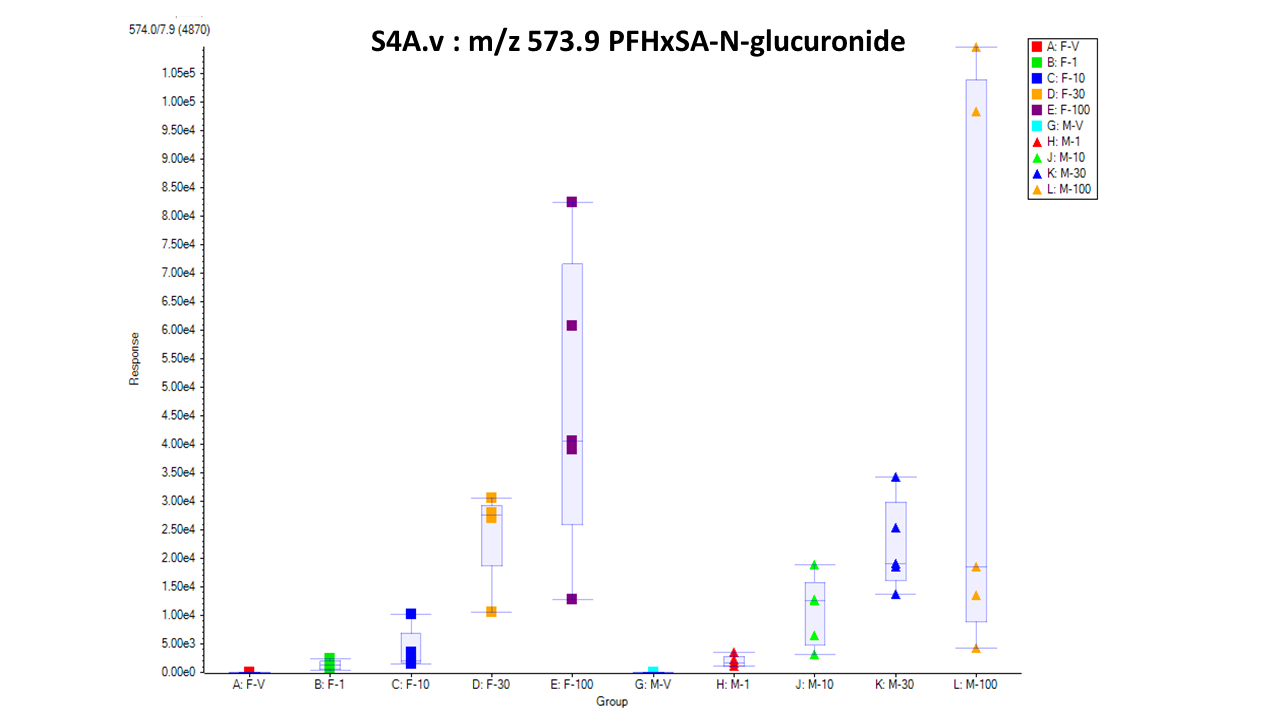

Supplement: Supplementary file 1 [file toxics-13-00523-s001.zip › Fig S4-A-Peak Areas for Detections in Plasma-rev1/Slide24.PNG]

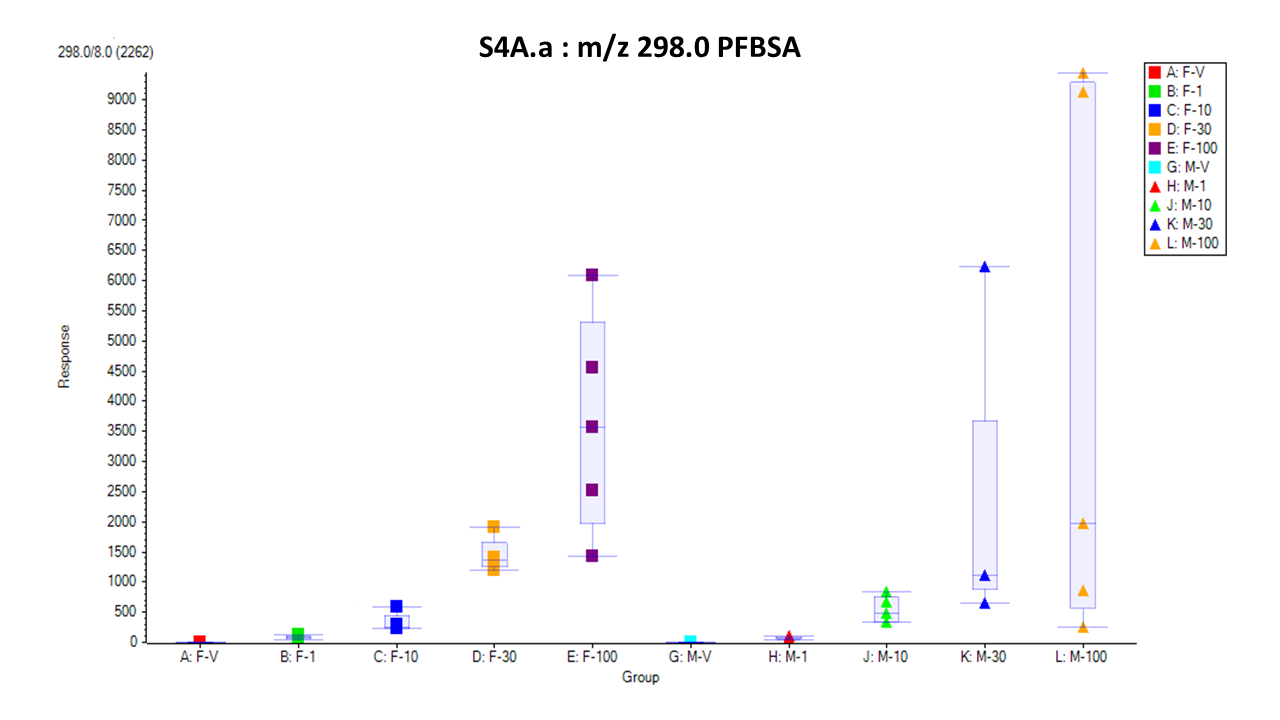

Supplement: Supplementary file 1 [file toxics-13-00523-s001.zip › Fig S4-A-Peak Areas for Detections in Plasma-rev1/Slide3.PNG]

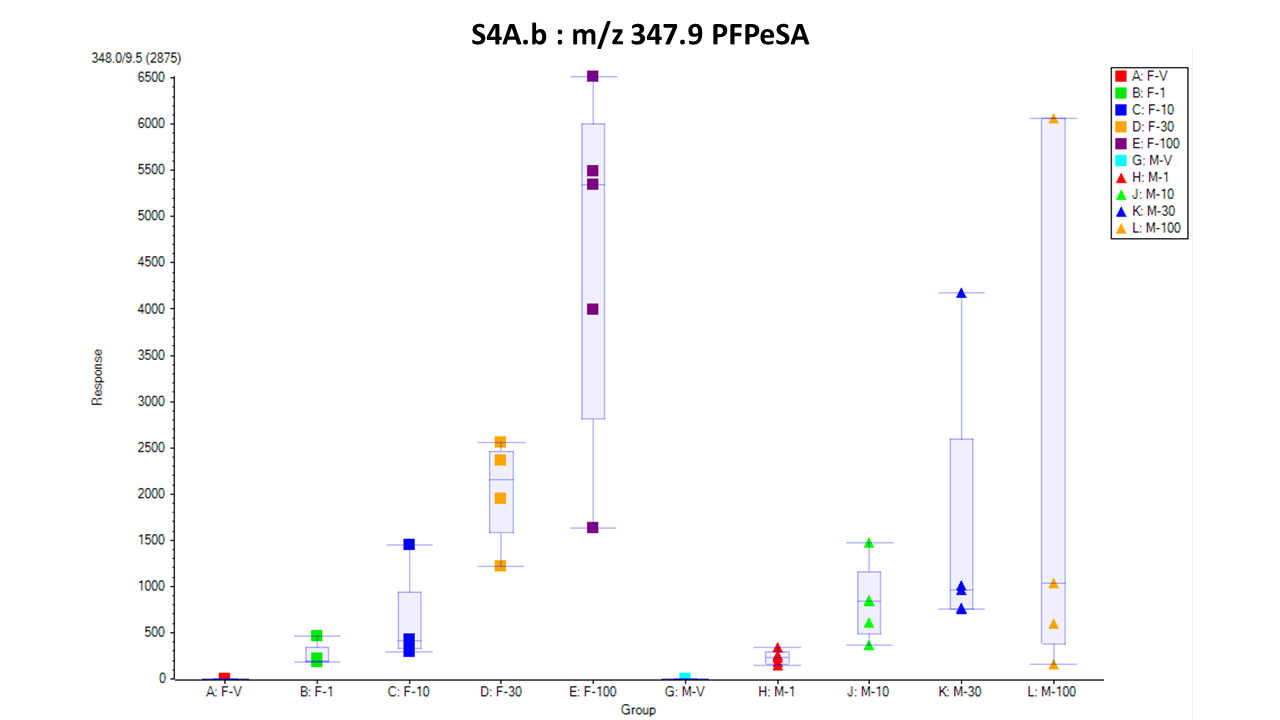

Supplement: Supplementary file 1 [file toxics-13-00523-s001.zip › Fig S4-A-Peak Areas for Detections in Plasma-rev1/Slide4.PNG]

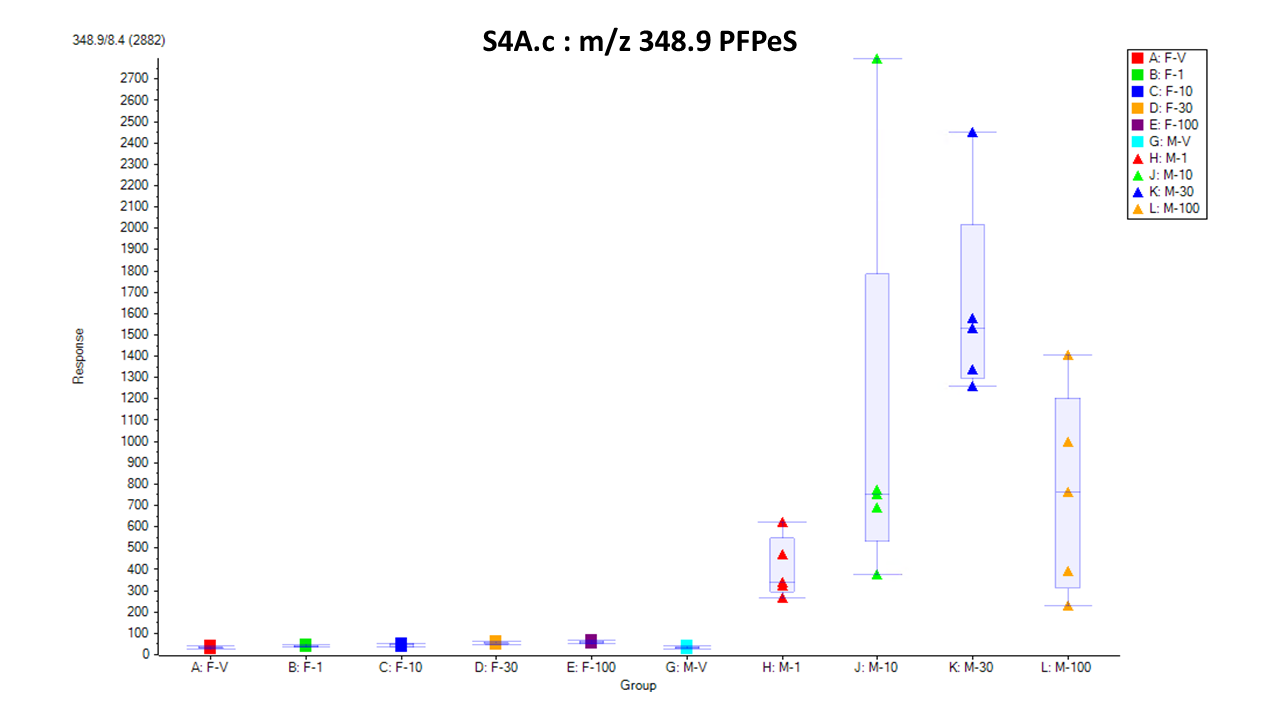

Supplement: Supplementary file 1 [file toxics-13-00523-s001.zip › Fig S4-A-Peak Areas for Detections in Plasma-rev1/Slide5.PNG]

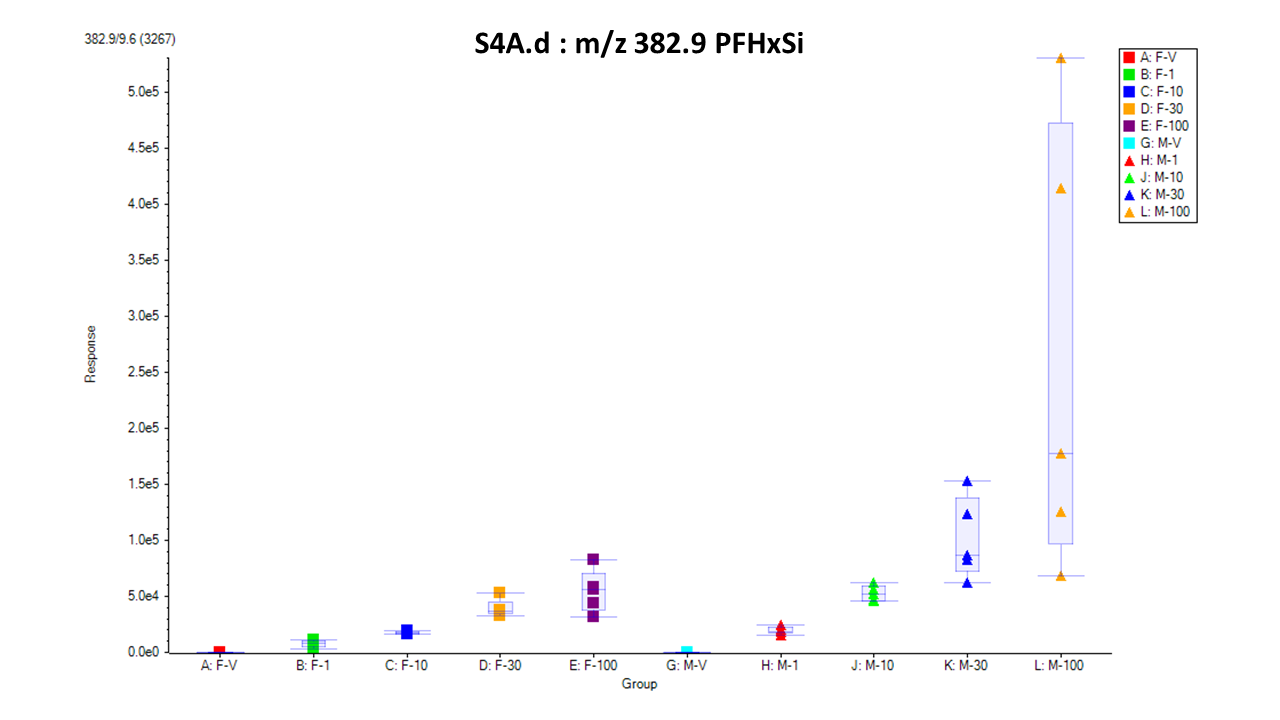

Supplement: Supplementary file 1 [file toxics-13-00523-s001.zip › Fig S4-A-Peak Areas for Detections in Plasma-rev1/Slide6.PNG]

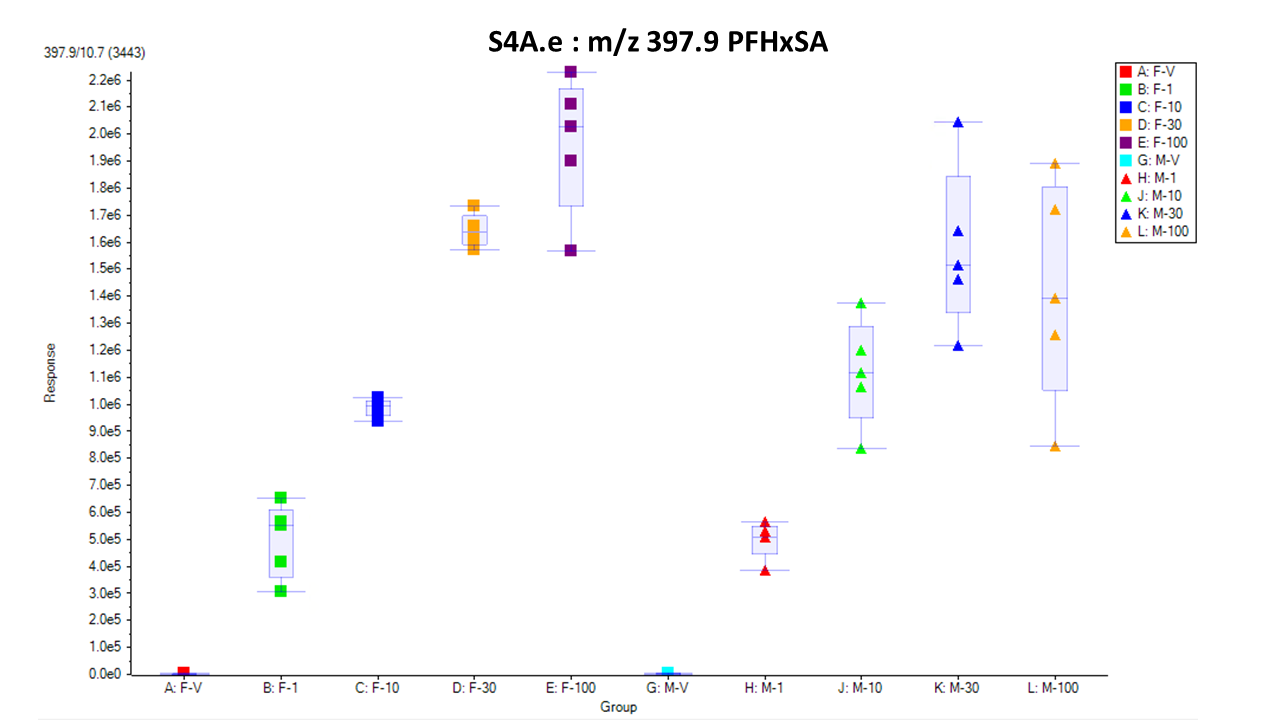

Supplement: Supplementary file 1 [file toxics-13-00523-s001.zip › Fig S4-A-Peak Areas for Detections in Plasma-rev1/Slide7.PNG]

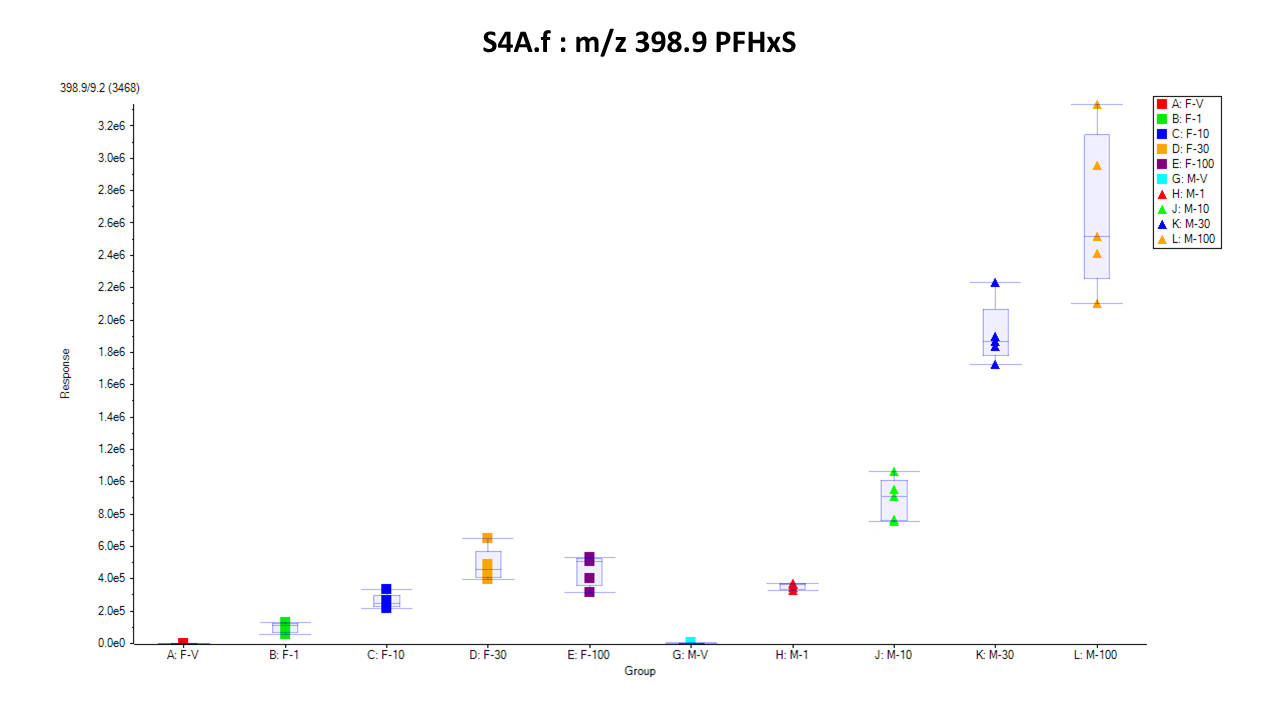

Supplement: Supplementary file 1 [file toxics-13-00523-s001.zip › Fig S4-A-Peak Areas for Detections in Plasma-rev1/Slide8.PNG]

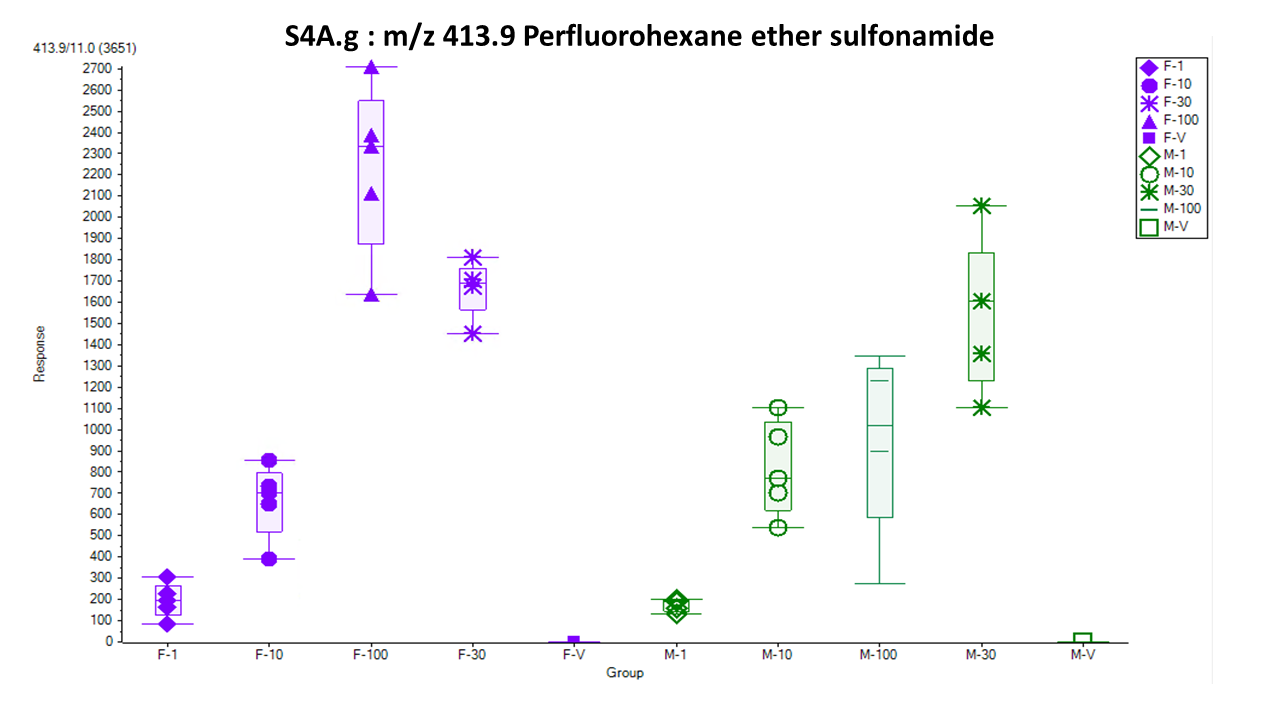

Supplement: Supplementary file 1 [file toxics-13-00523-s001.zip › Fig S4-A-Peak Areas for Detections in Plasma-rev1/Slide9.PNG]

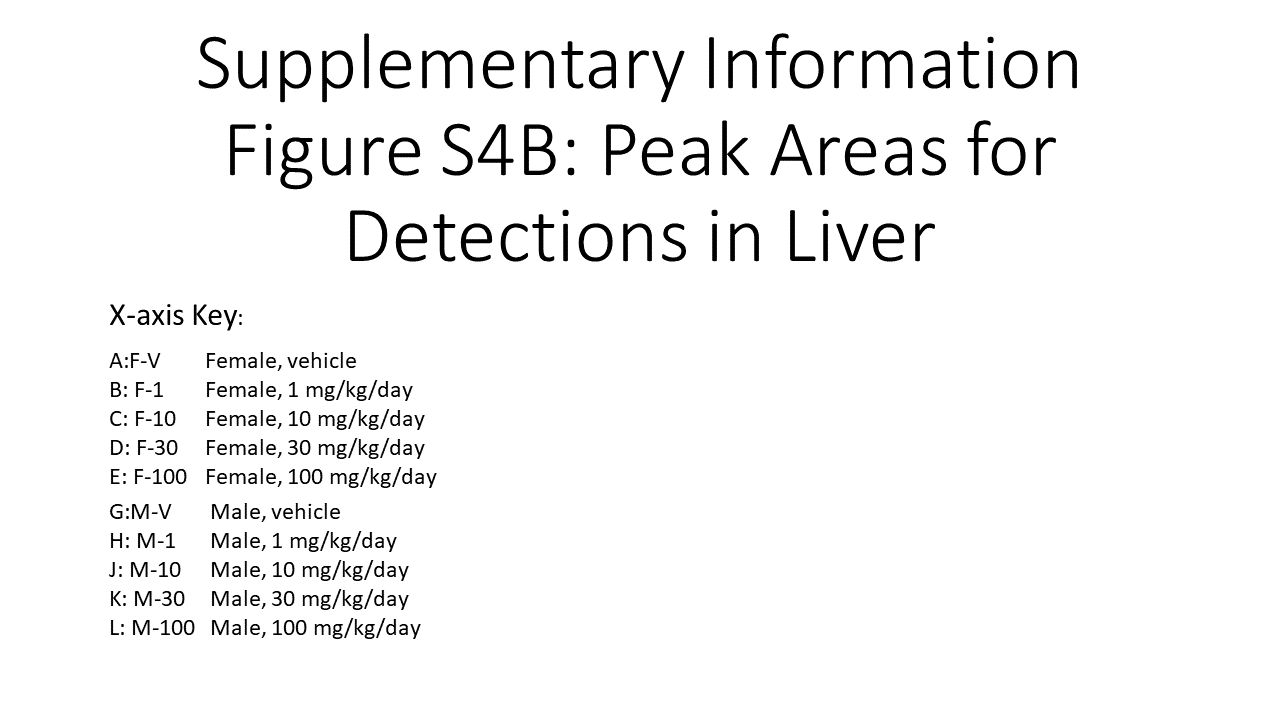

Supplement: Supplementary file 1 [file toxics-13-00523-s001.zip › Fig S4-B-Peak Areas for Detections in Liver-rev1/Slide1.PNG]

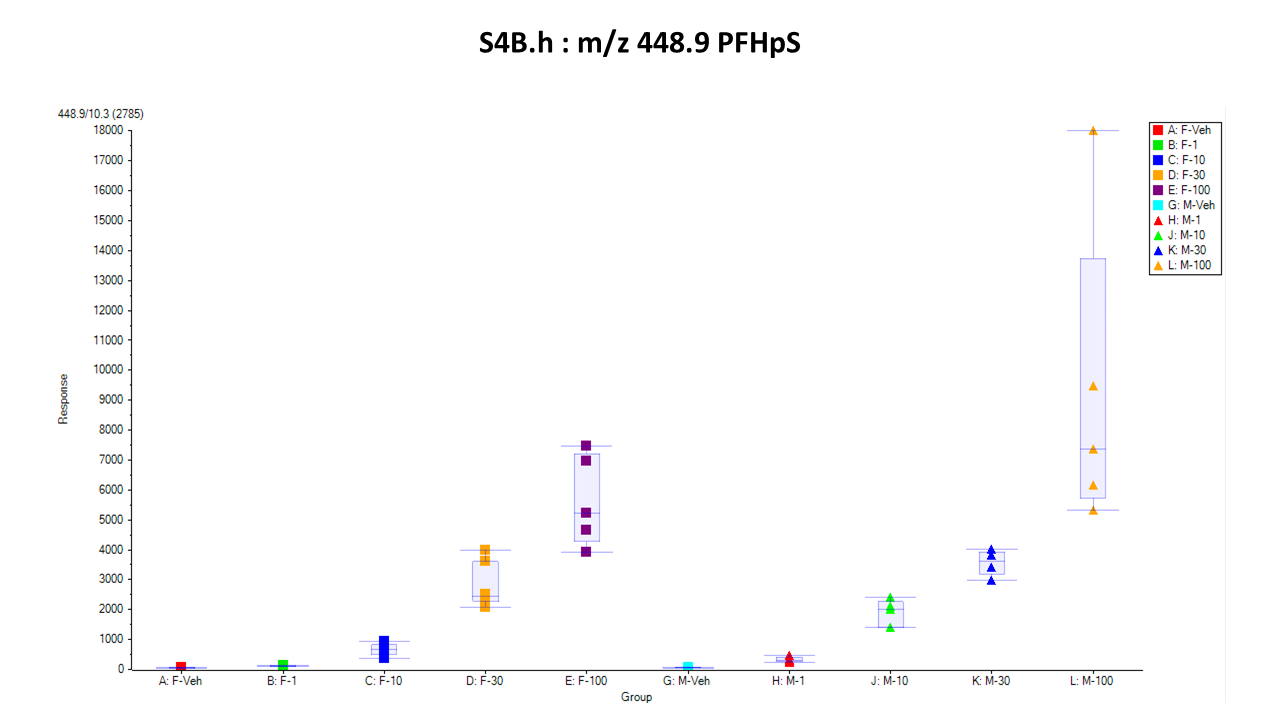

Supplement: Supplementary file 1 [file toxics-13-00523-s001.zip › Fig S4-B-Peak Areas for Detections in Liver-rev1/Slide10.PNG]

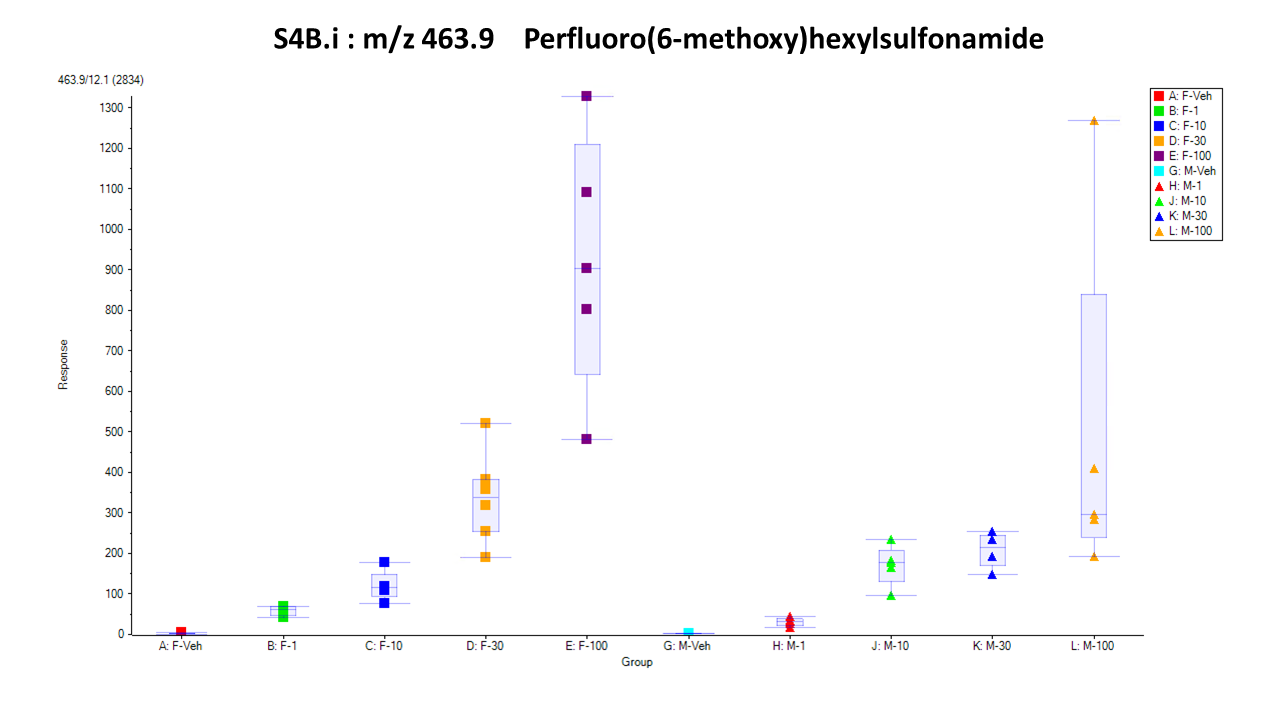

Supplement: Supplementary file 1 [file toxics-13-00523-s001.zip › Fig S4-B-Peak Areas for Detections in Liver-rev1/Slide11.PNG]

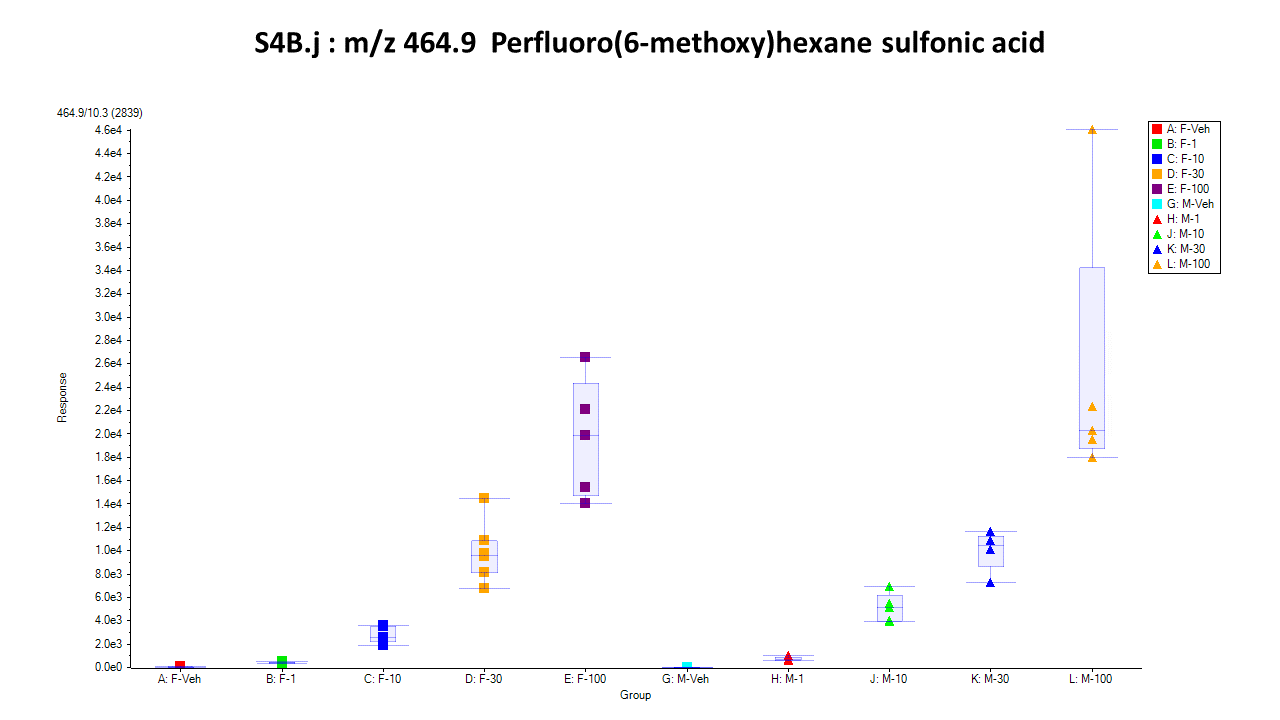

Supplement: Supplementary file 1 [file toxics-13-00523-s001.zip › Fig S4-B-Peak Areas for Detections in Liver-rev1/Slide12.PNG]

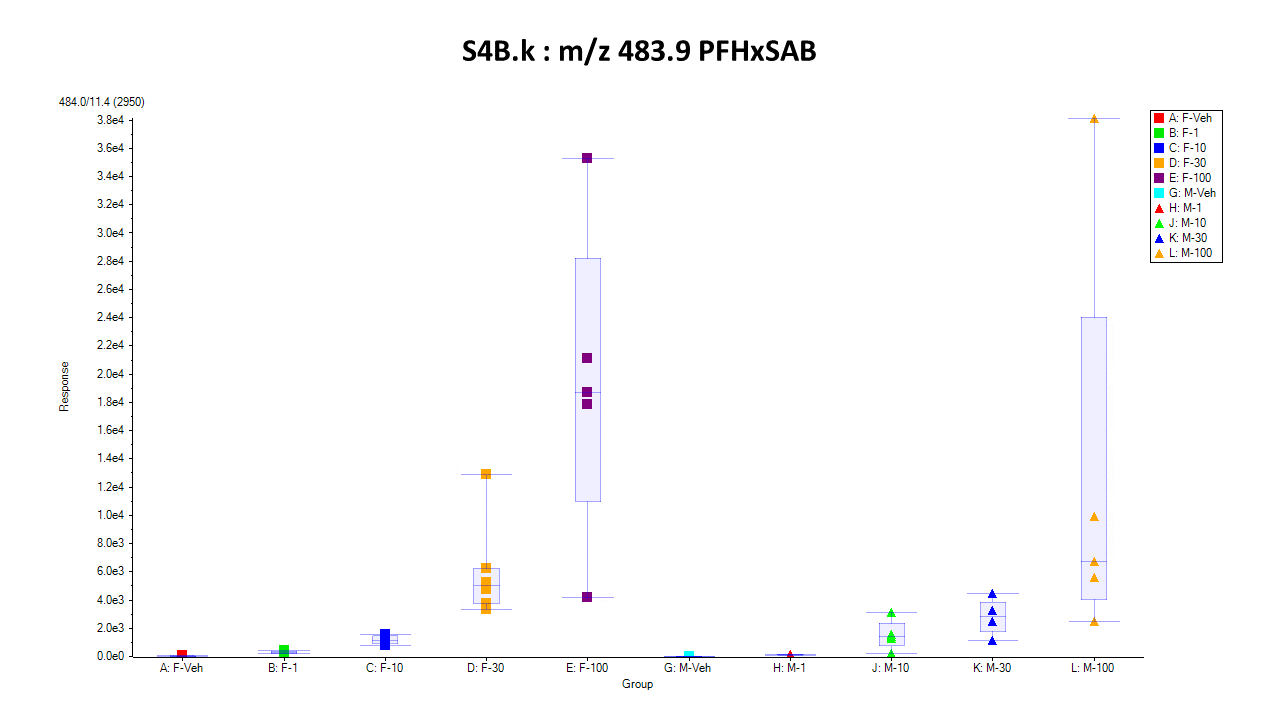

Supplement: Supplementary file 1 [file toxics-13-00523-s001.zip › Fig S4-B-Peak Areas for Detections in Liver-rev1/Slide13.PNG]

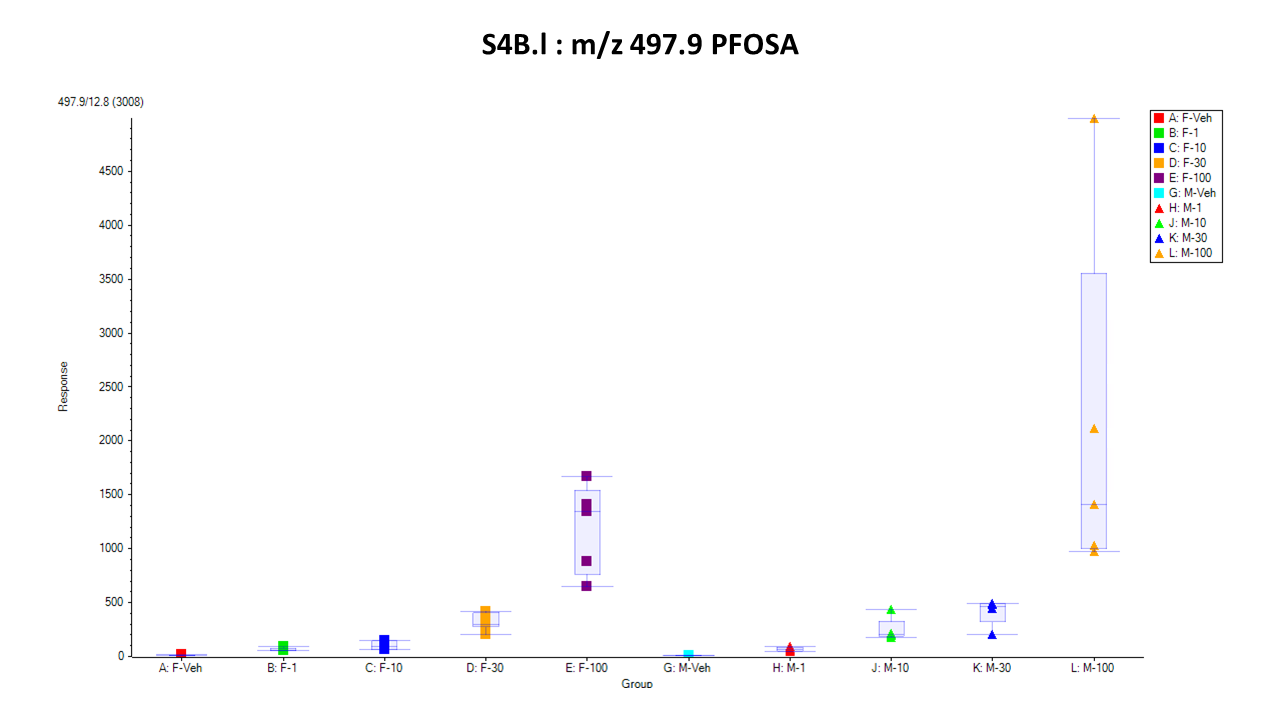

Supplement: Supplementary file 1 [file toxics-13-00523-s001.zip › Fig S4-B-Peak Areas for Detections in Liver-rev1/Slide14.PNG]

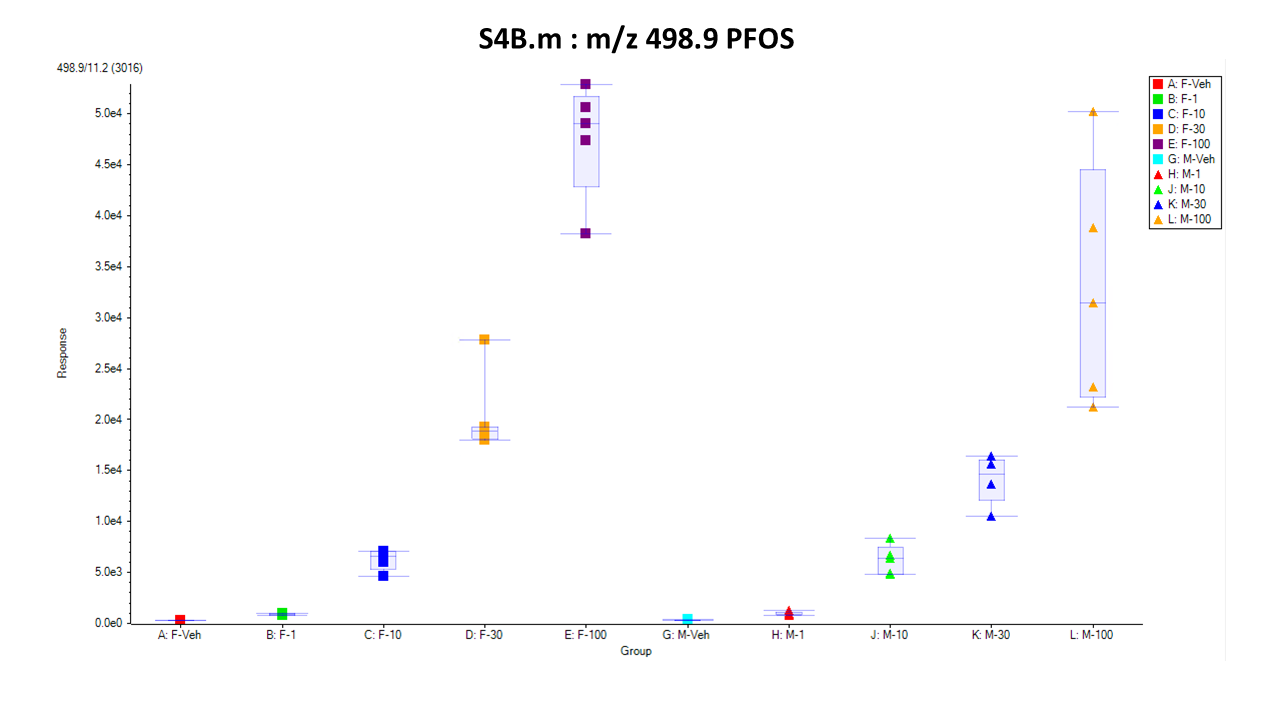

Supplement: Supplementary file 1 [file toxics-13-00523-s001.zip › Fig S4-B-Peak Areas for Detections in Liver-rev1/Slide15.PNG]

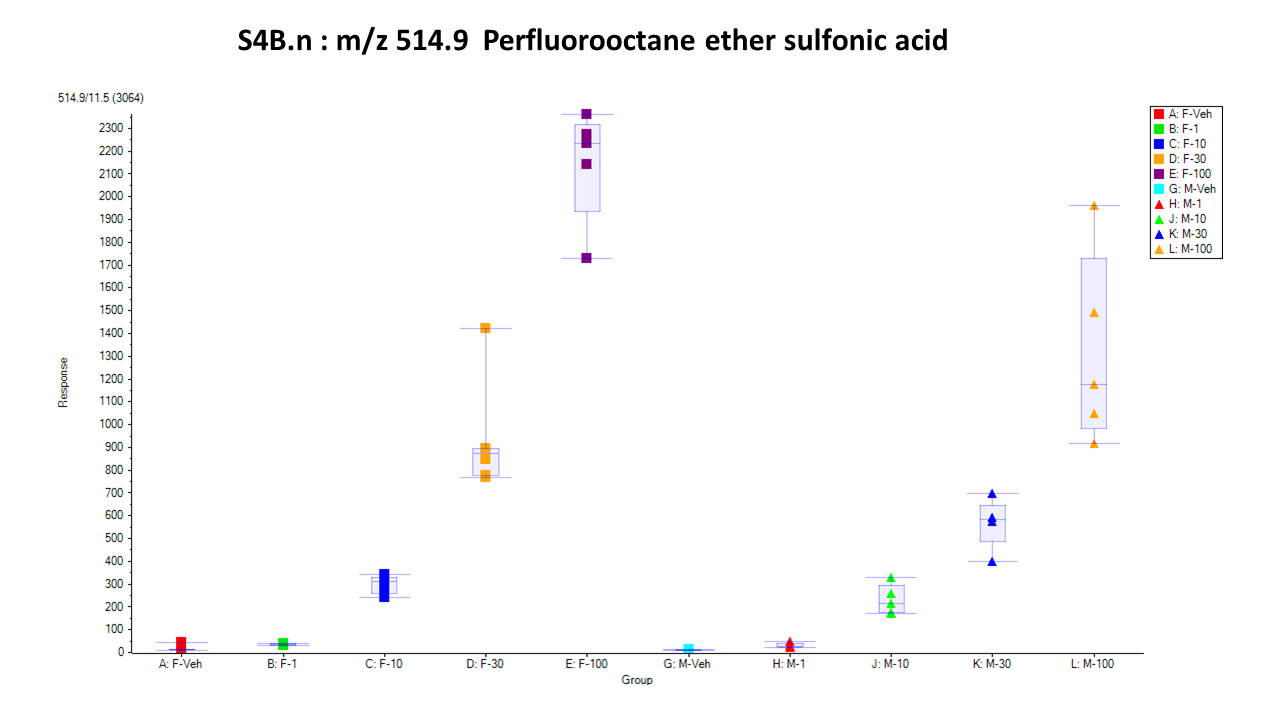

Supplement: Supplementary file 1 [file toxics-13-00523-s001.zip › Fig S4-B-Peak Areas for Detections in Liver-rev1/Slide16.PNG]

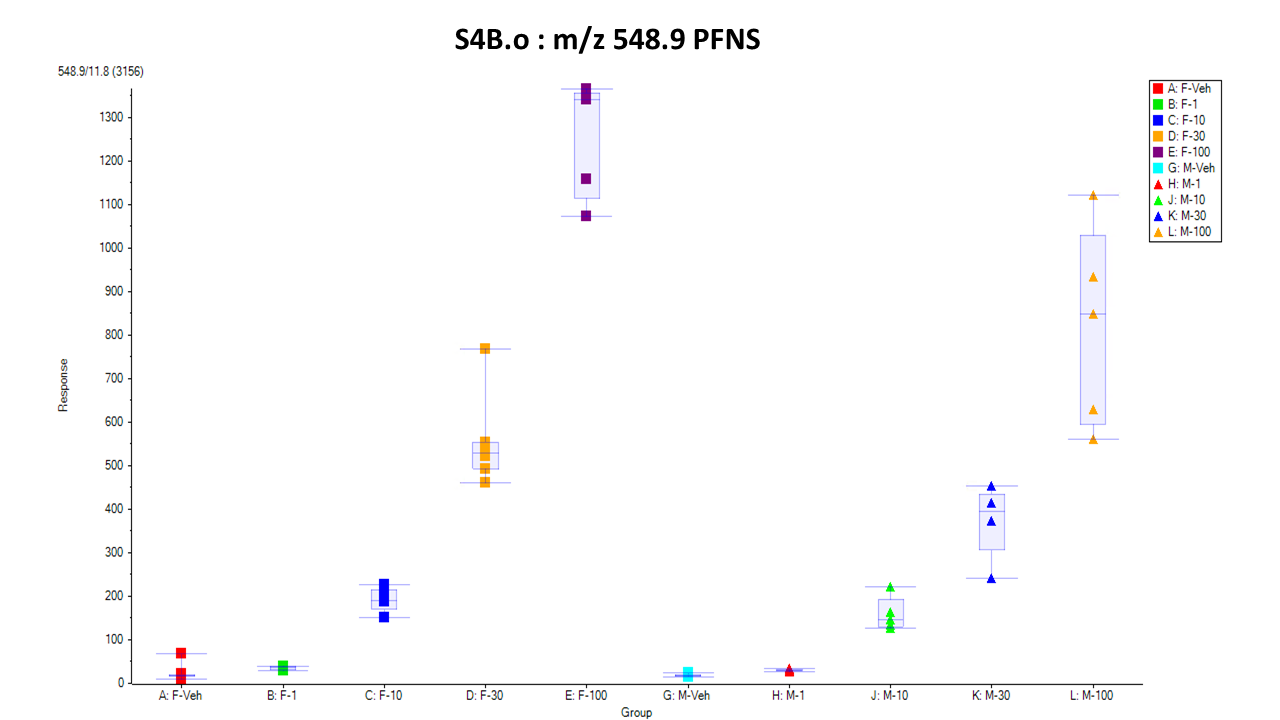

Supplement: Supplementary file 1 [file toxics-13-00523-s001.zip › Fig S4-B-Peak Areas for Detections in Liver-rev1/Slide17.PNG]

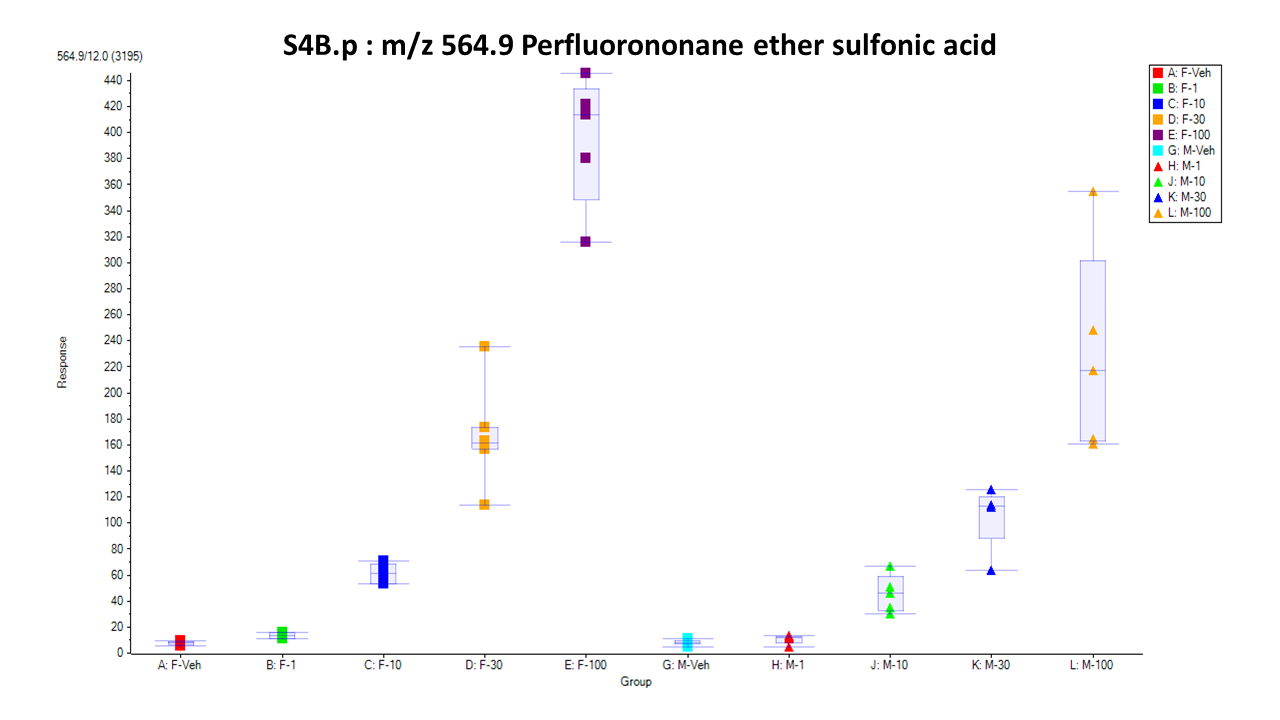

Supplement: Supplementary file 1 [file toxics-13-00523-s001.zip › Fig S4-B-Peak Areas for Detections in Liver-rev1/Slide18.PNG]

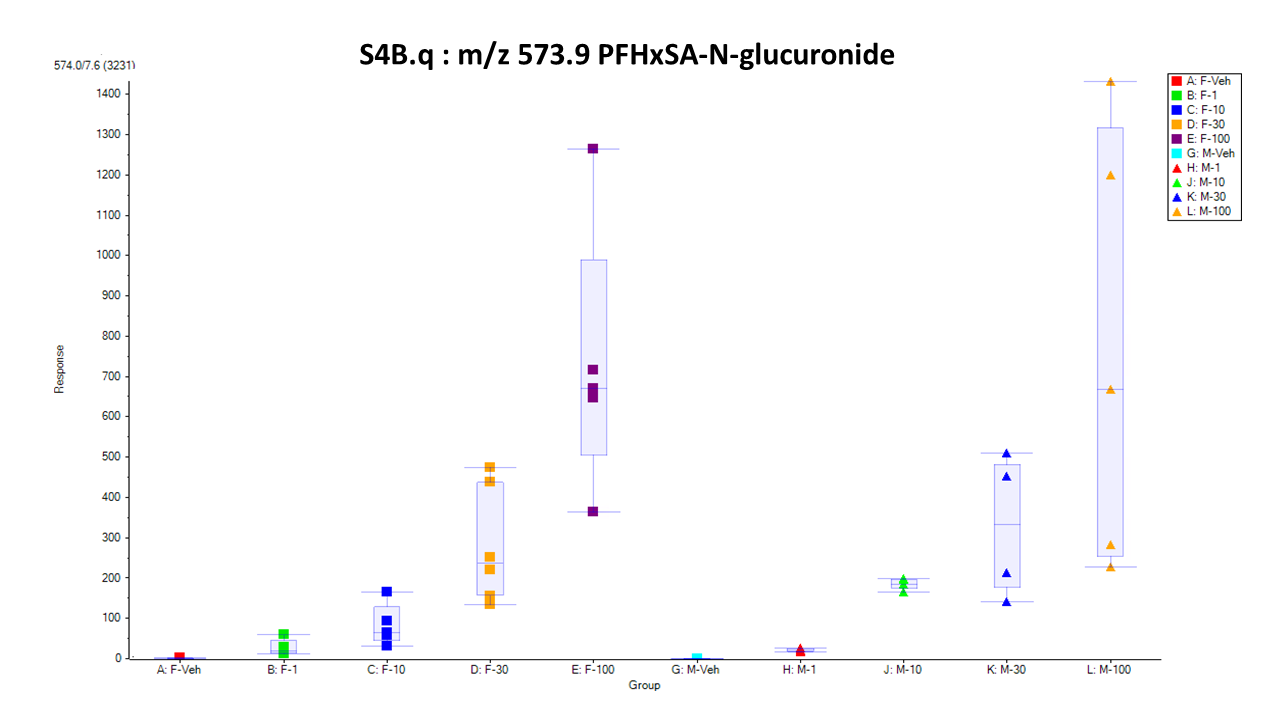

Supplement: Supplementary file 1 [file toxics-13-00523-s001.zip › Fig S4-B-Peak Areas for Detections in Liver-rev1/Slide19.PNG]

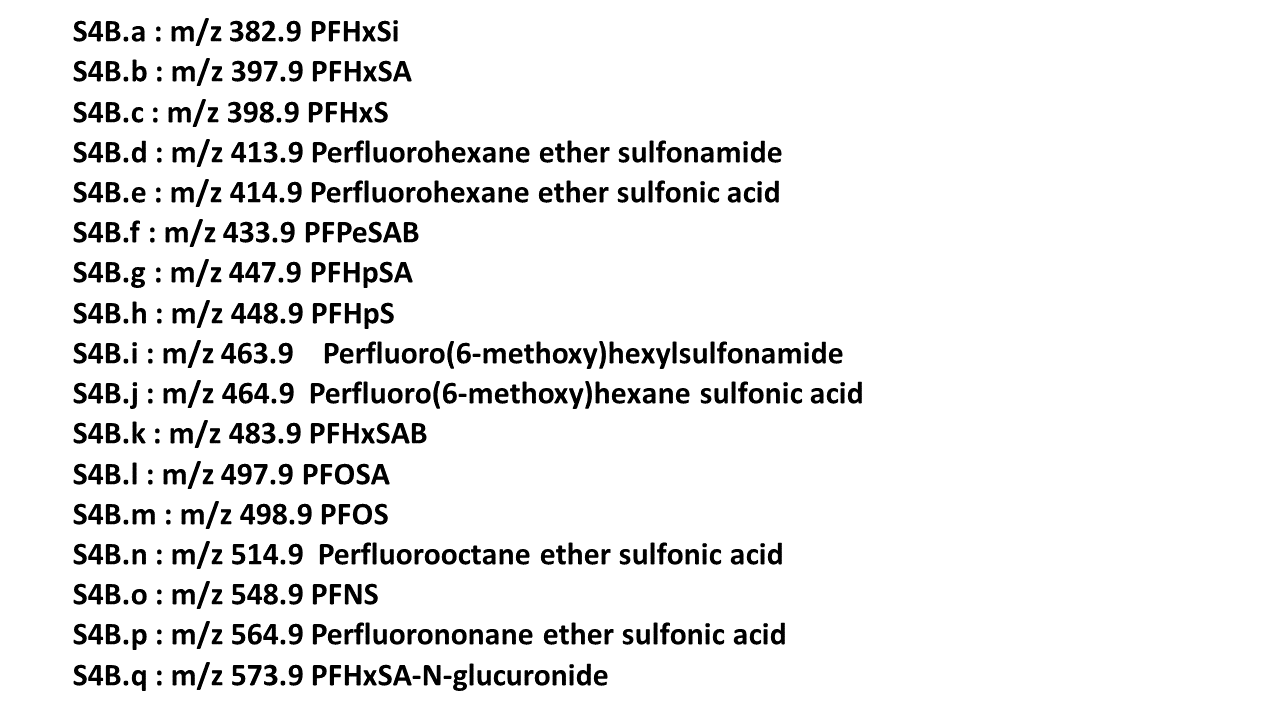

Supplement: Supplementary file 1 [file toxics-13-00523-s001.zip › Fig S4-B-Peak Areas for Detections in Liver-rev1/Slide2.PNG]

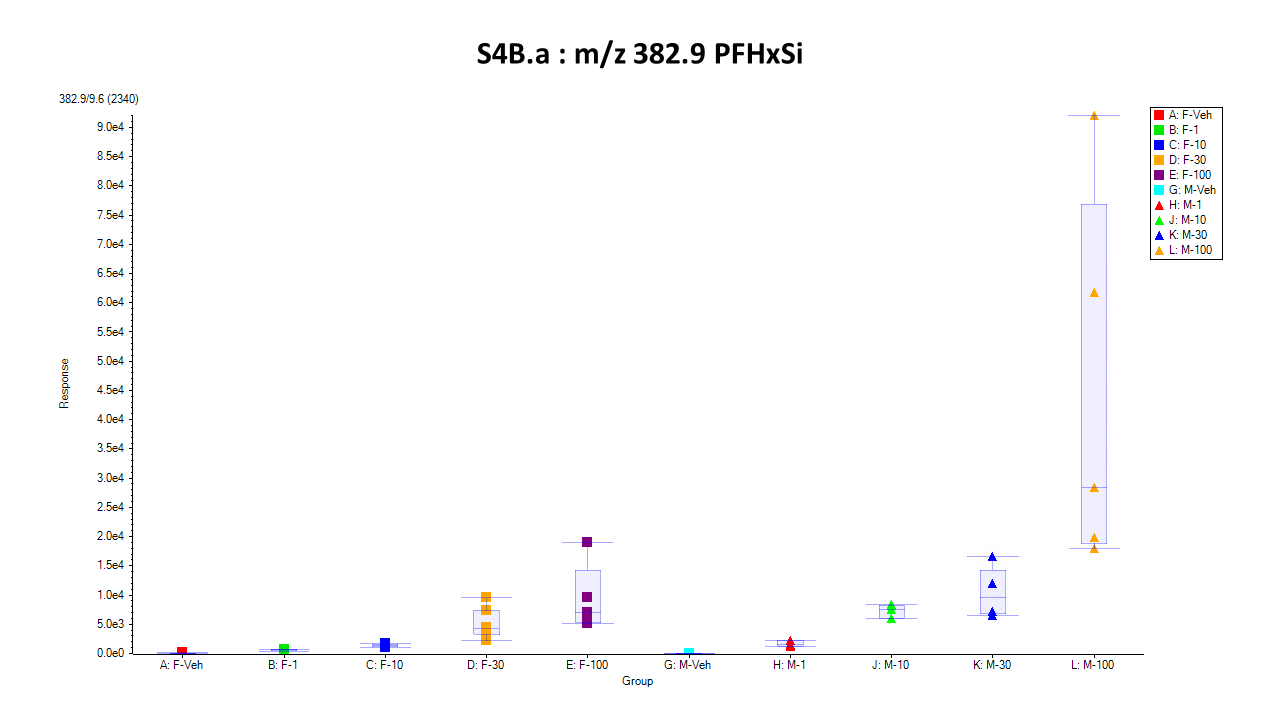

Supplement: Supplementary file 1 [file toxics-13-00523-s001.zip › Fig S4-B-Peak Areas for Detections in Liver-rev1/Slide3.PNG]

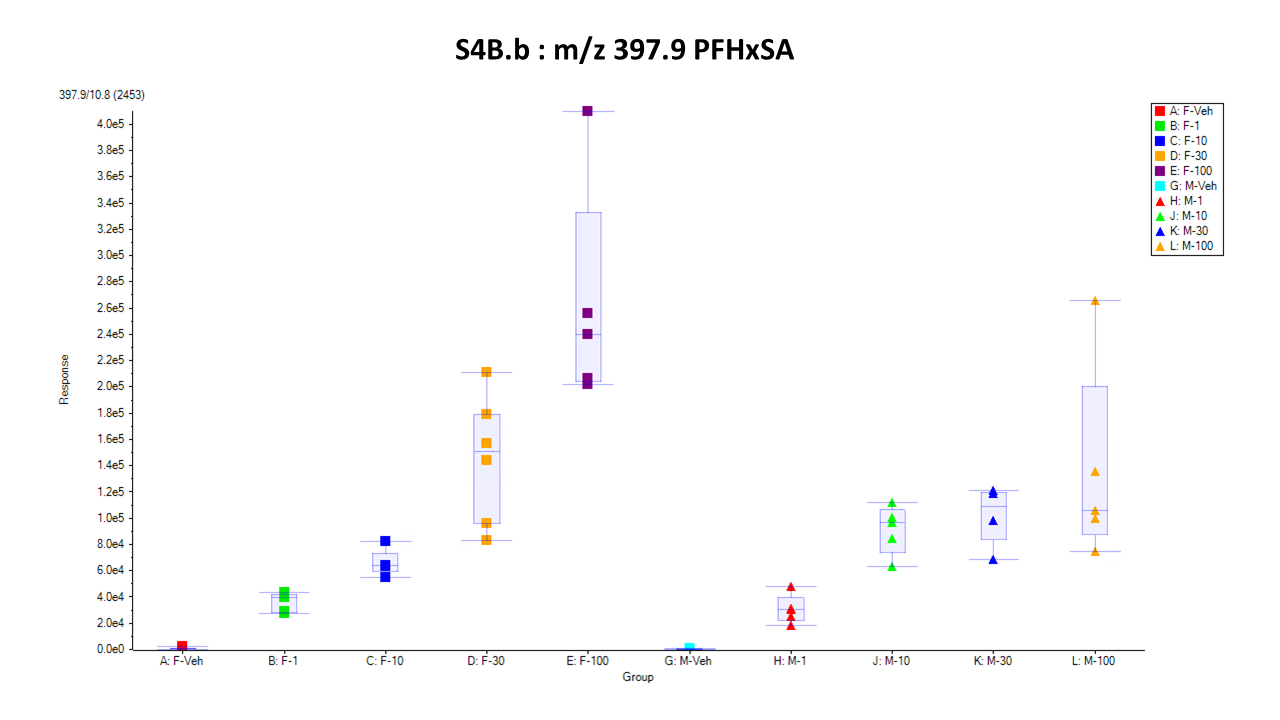

Supplement: Supplementary file 1 [file toxics-13-00523-s001.zip › Fig S4-B-Peak Areas for Detections in Liver-rev1/Slide4.PNG]

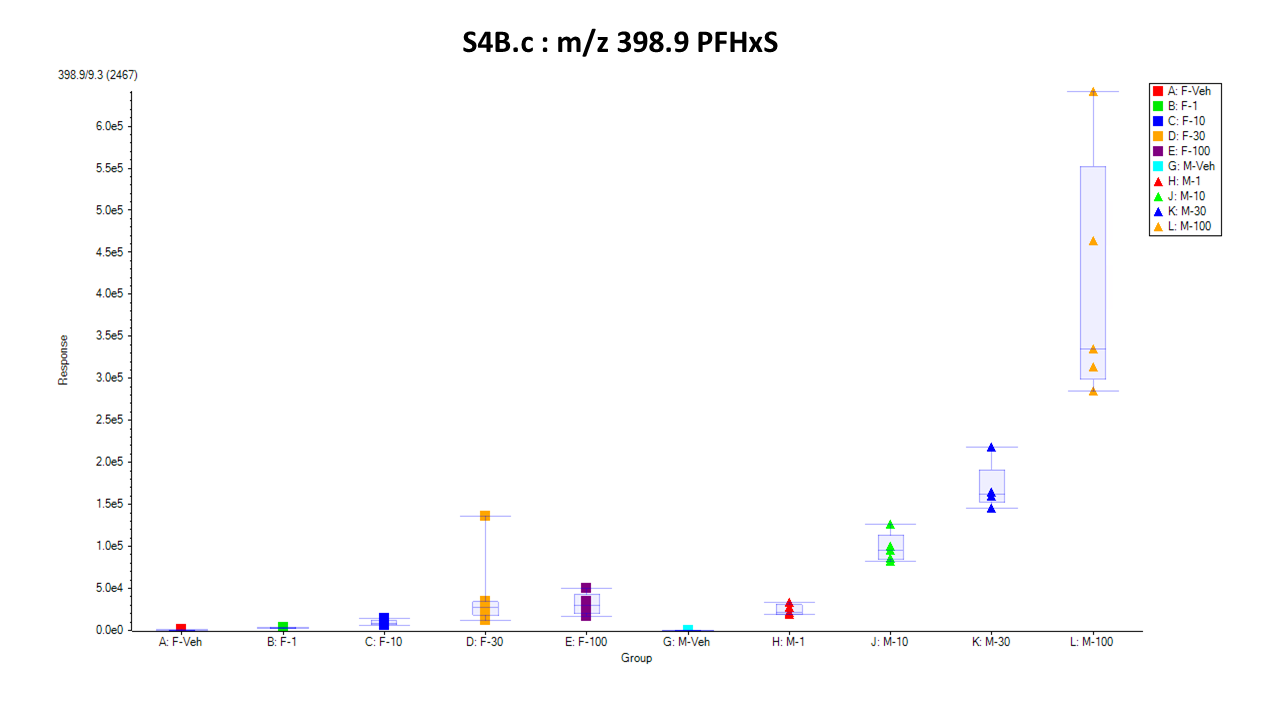

Supplement: Supplementary file 1 [file toxics-13-00523-s001.zip › Fig S4-B-Peak Areas for Detections in Liver-rev1/Slide5.PNG]

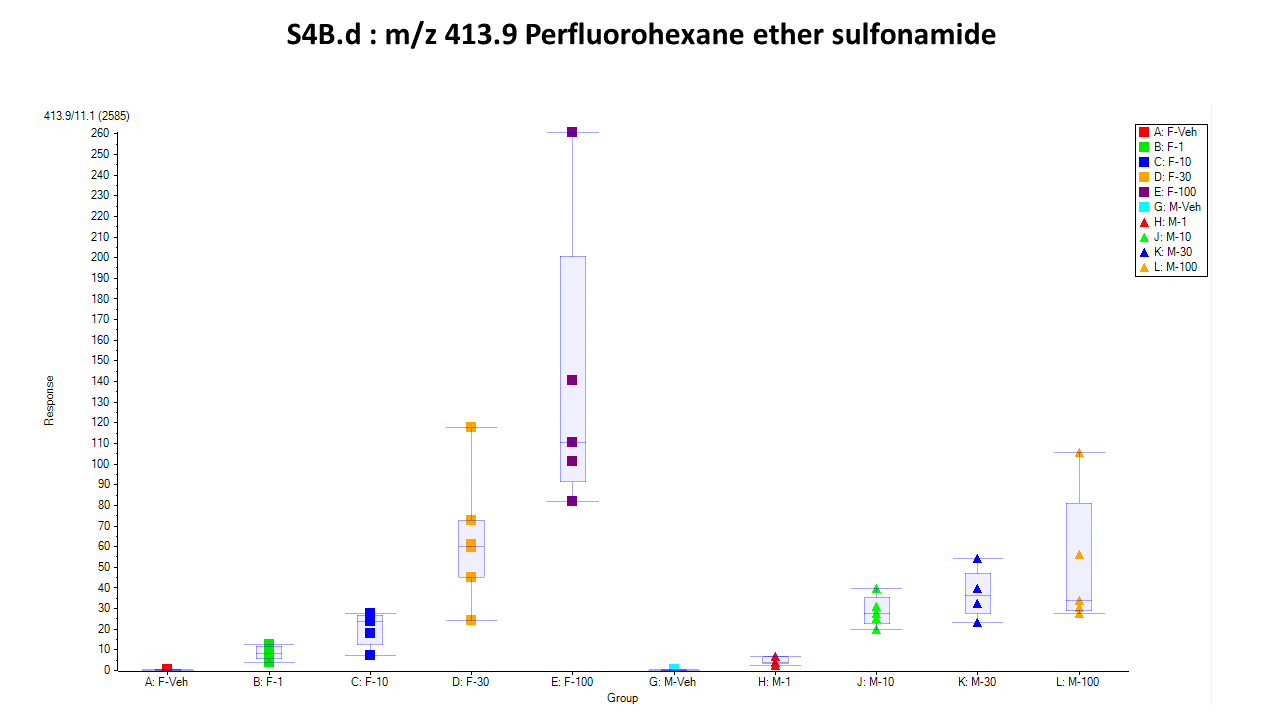

Supplement: Supplementary file 1 [file toxics-13-00523-s001.zip › Fig S4-B-Peak Areas for Detections in Liver-rev1/Slide6.PNG]

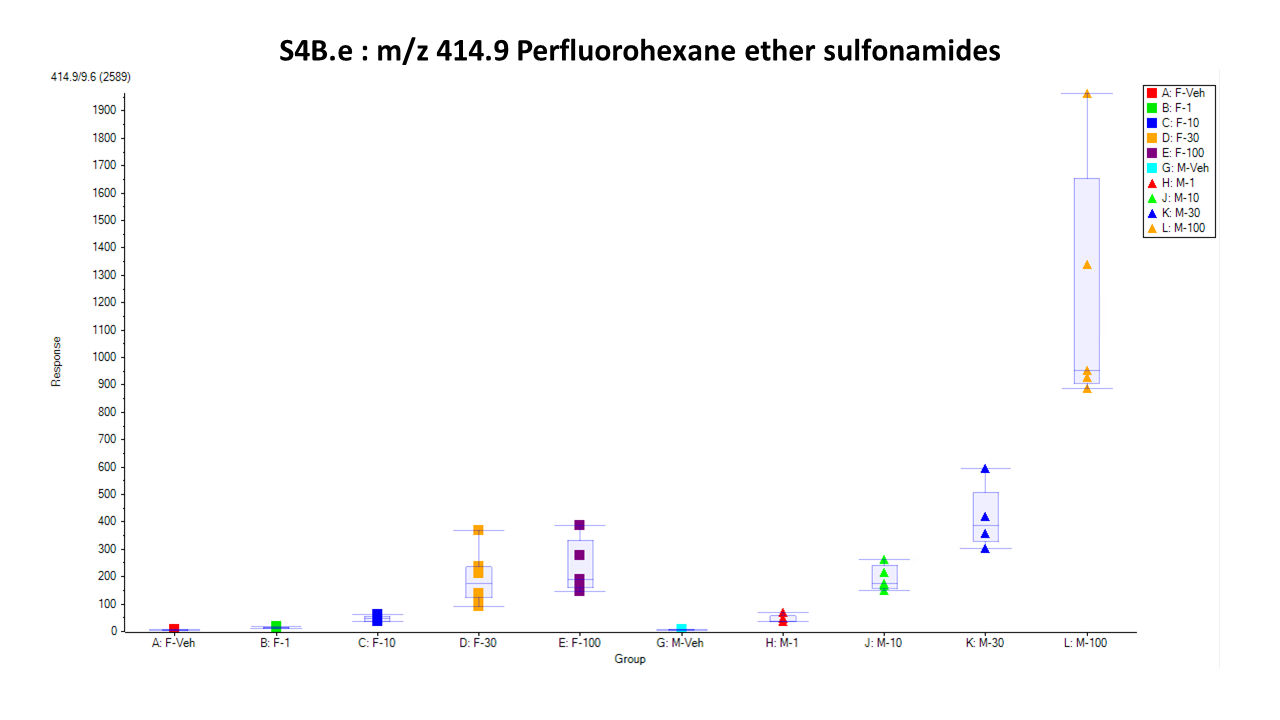

Supplement: Supplementary file 1 [file toxics-13-00523-s001.zip › Fig S4-B-Peak Areas for Detections in Liver-rev1/Slide7.PNG]

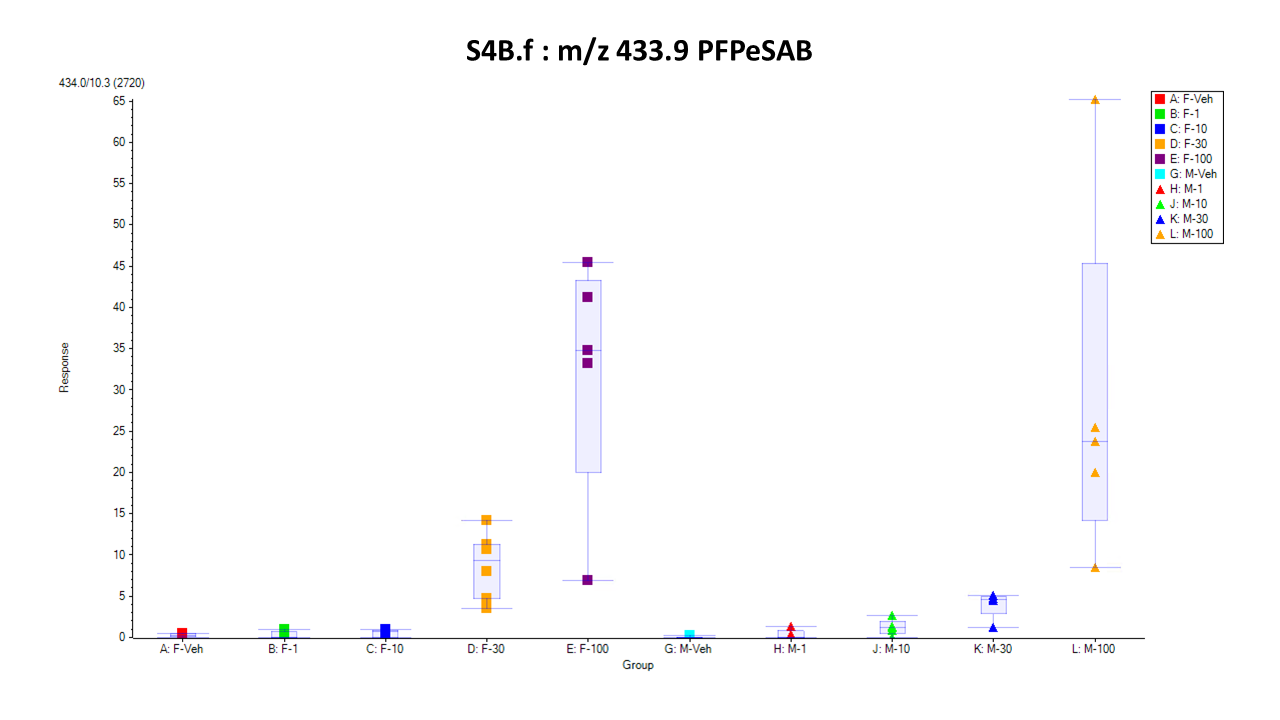

Supplement: Supplementary file 1 [file toxics-13-00523-s001.zip › Fig S4-B-Peak Areas for Detections in Liver-rev1/Slide8.PNG]

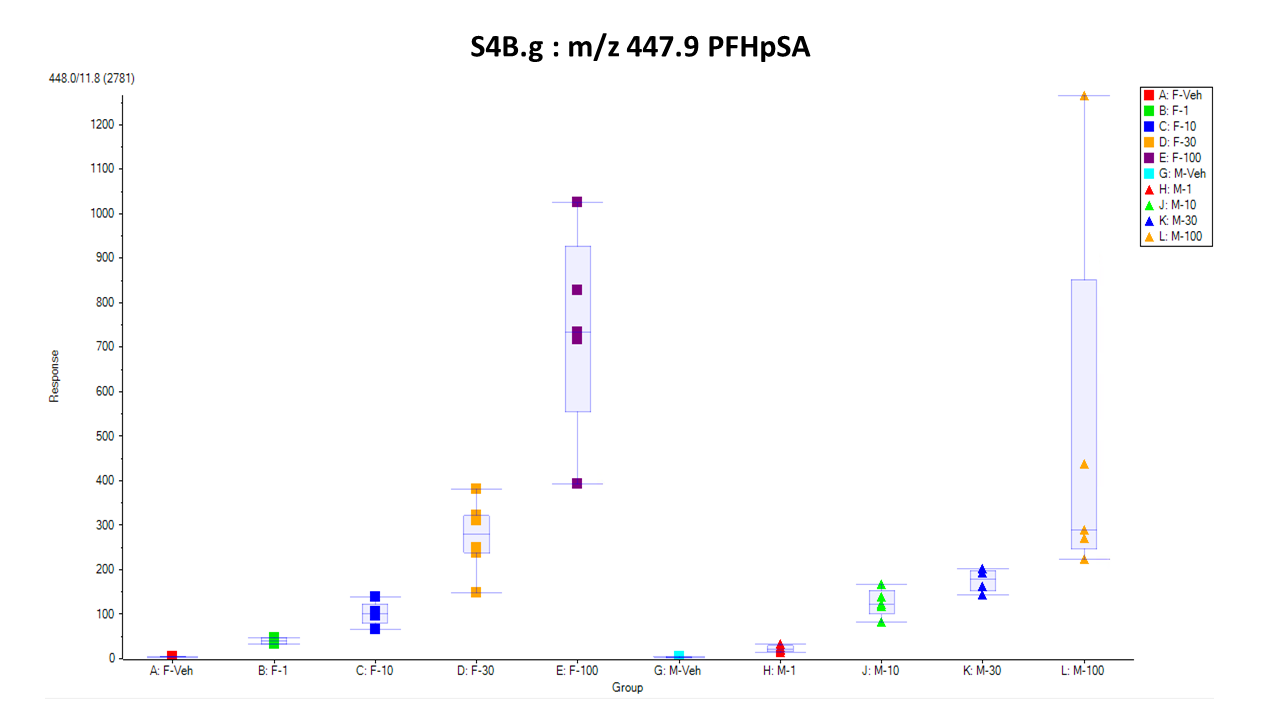

Supplement: Supplementary file 1 [file toxics-13-00523-s001.zip › Fig S4-B-Peak Areas for Detections in Liver-rev1/Slide9.PNG]

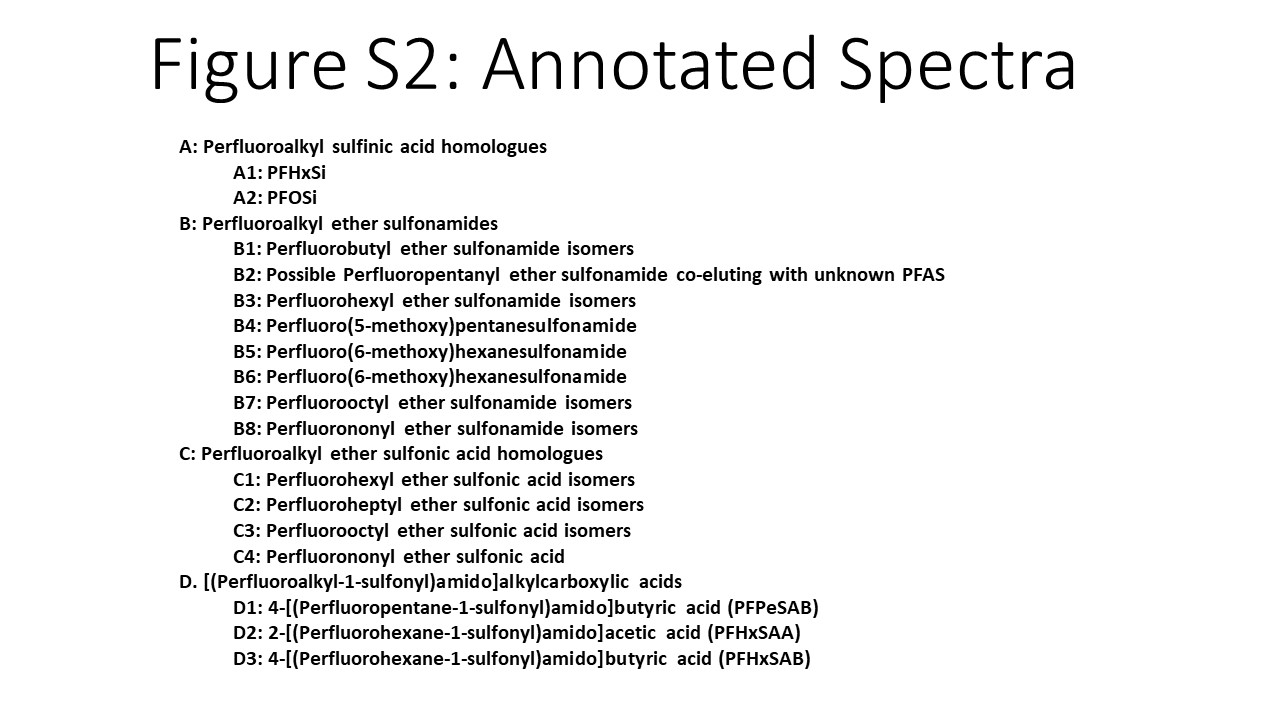

Supplement: Supplementary file 1 [file toxics-13-00523-s001.zip › SI Fig S2 Annotated Spectra/Slide1.JPG]

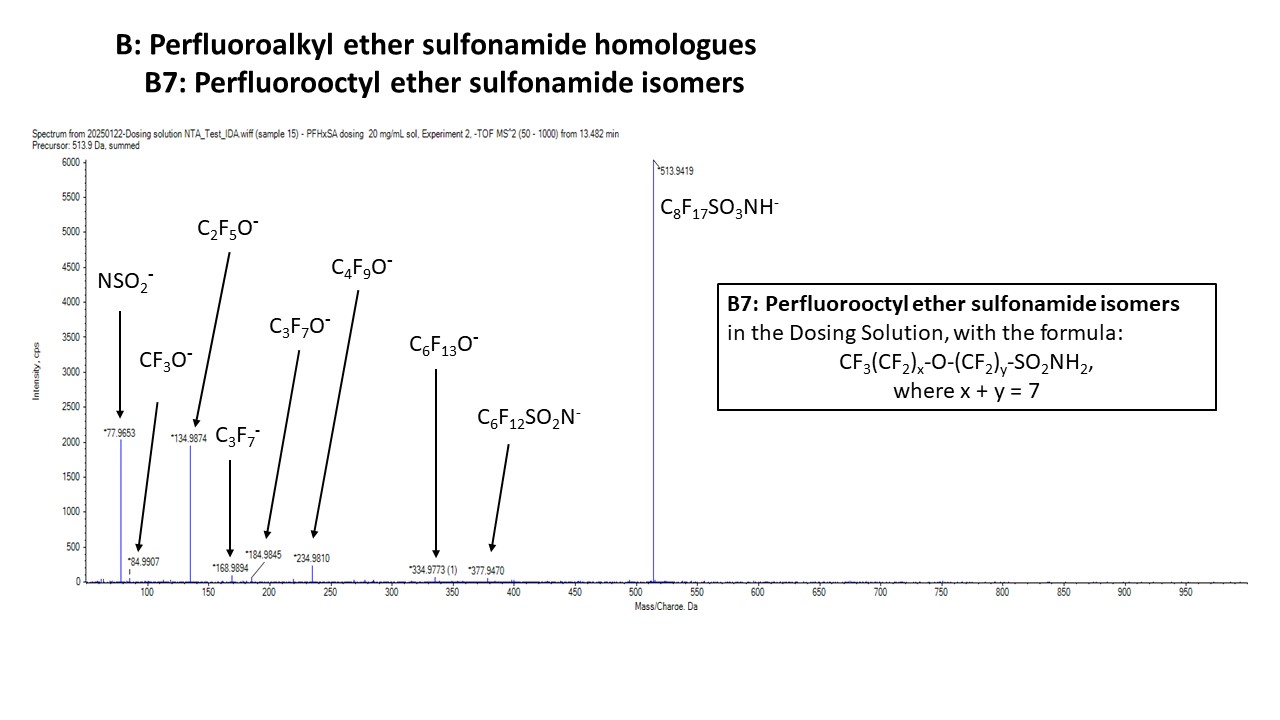

Supplement: Supplementary file 1 [file toxics-13-00523-s001.zip › SI Fig S2 Annotated Spectra/Slide10.JPG]

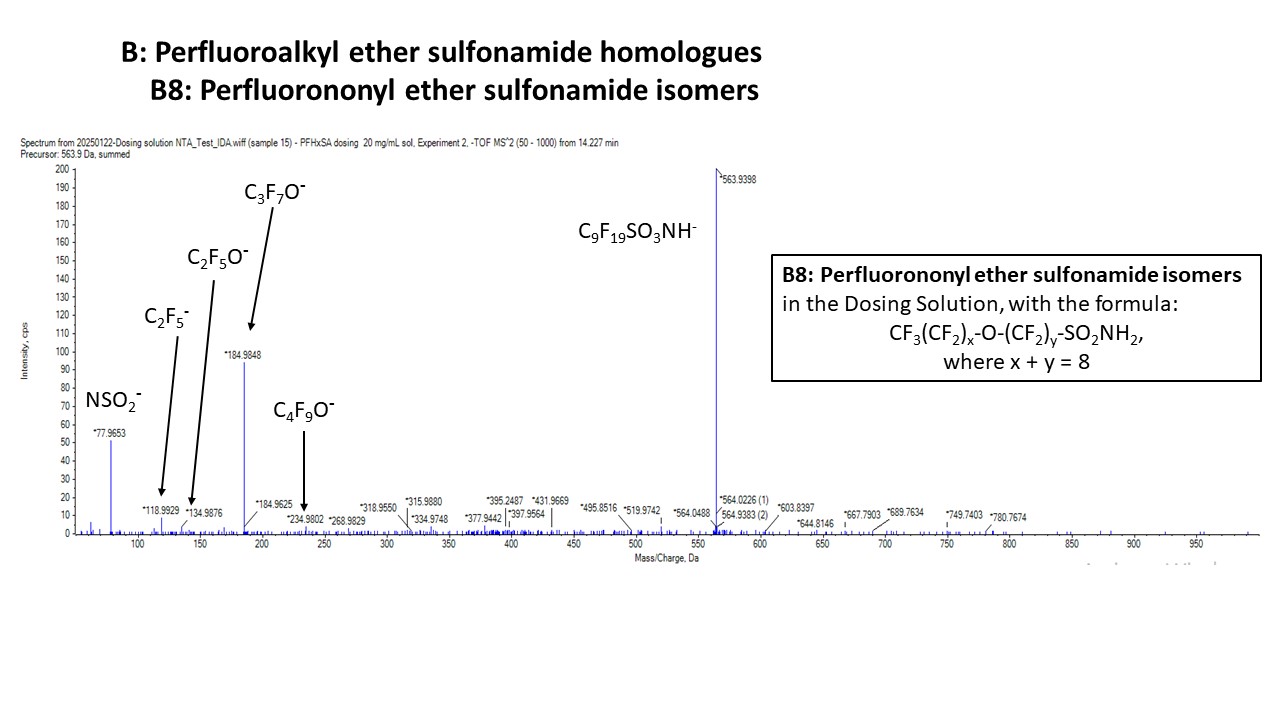

Supplement: Supplementary file 1 [file toxics-13-00523-s001.zip › SI Fig S2 Annotated Spectra/Slide11.JPG]

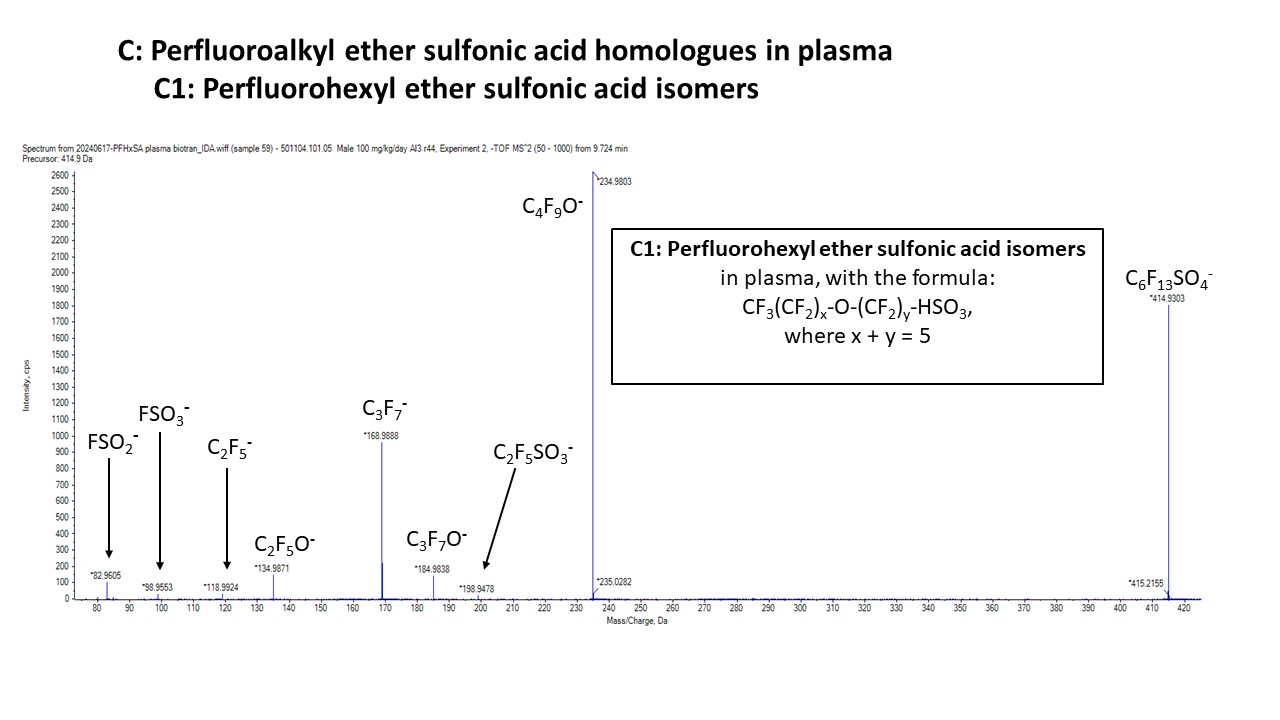

Supplement: Supplementary file 1 [file toxics-13-00523-s001.zip › SI Fig S2 Annotated Spectra/Slide12.JPG]

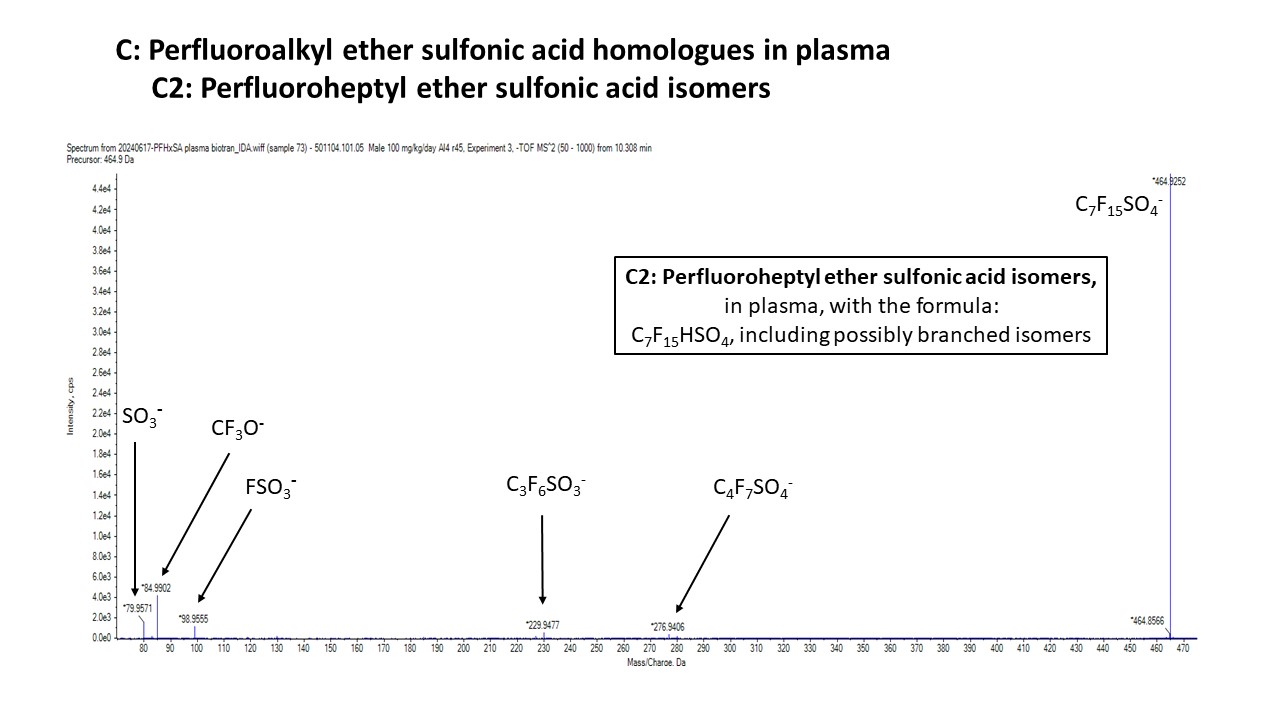

Supplement: Supplementary file 1 [file toxics-13-00523-s001.zip › SI Fig S2 Annotated Spectra/Slide13.JPG]

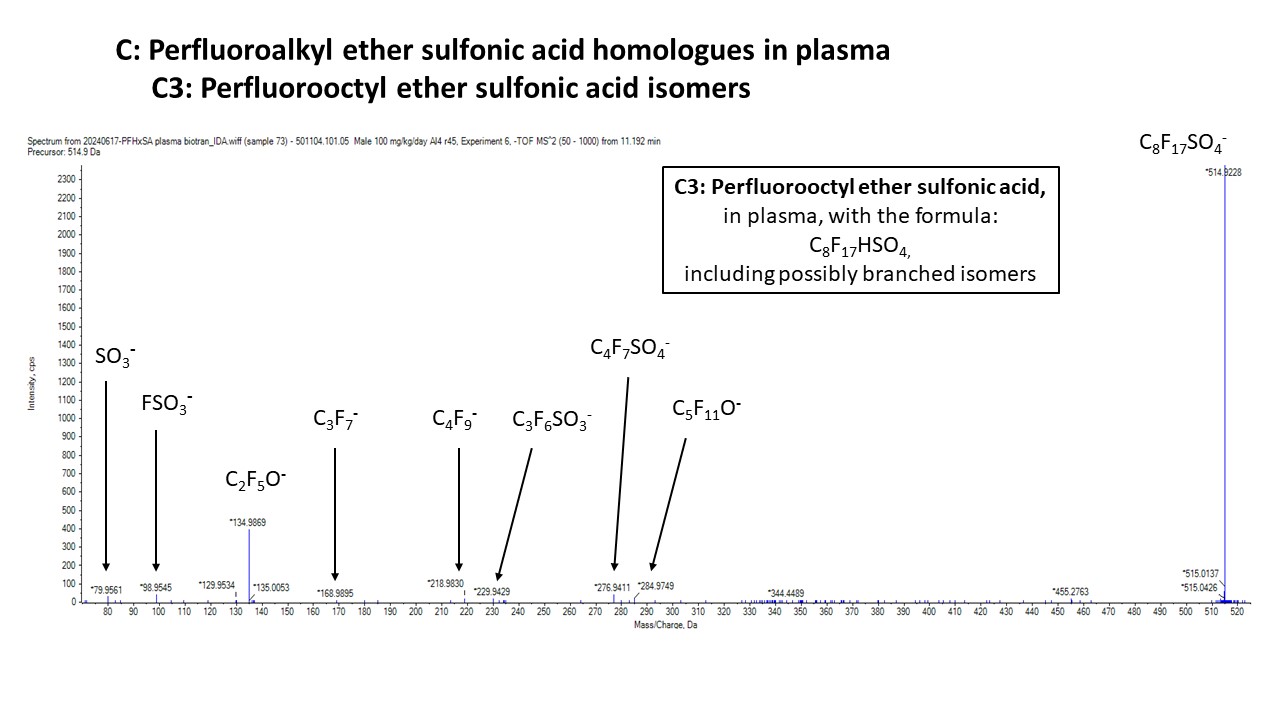

Supplement: Supplementary file 1 [file toxics-13-00523-s001.zip › SI Fig S2 Annotated Spectra/Slide14.JPG]

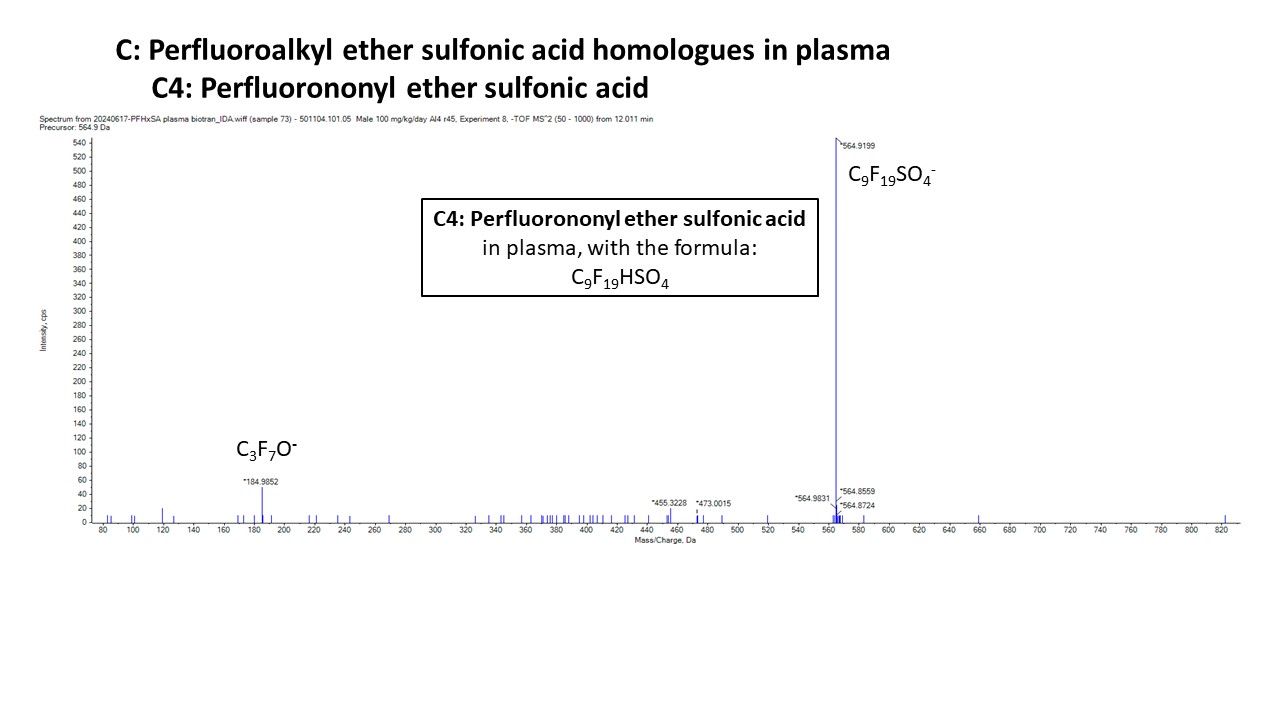

Supplement: Supplementary file 1 [file toxics-13-00523-s001.zip › SI Fig S2 Annotated Spectra/Slide15.JPG]

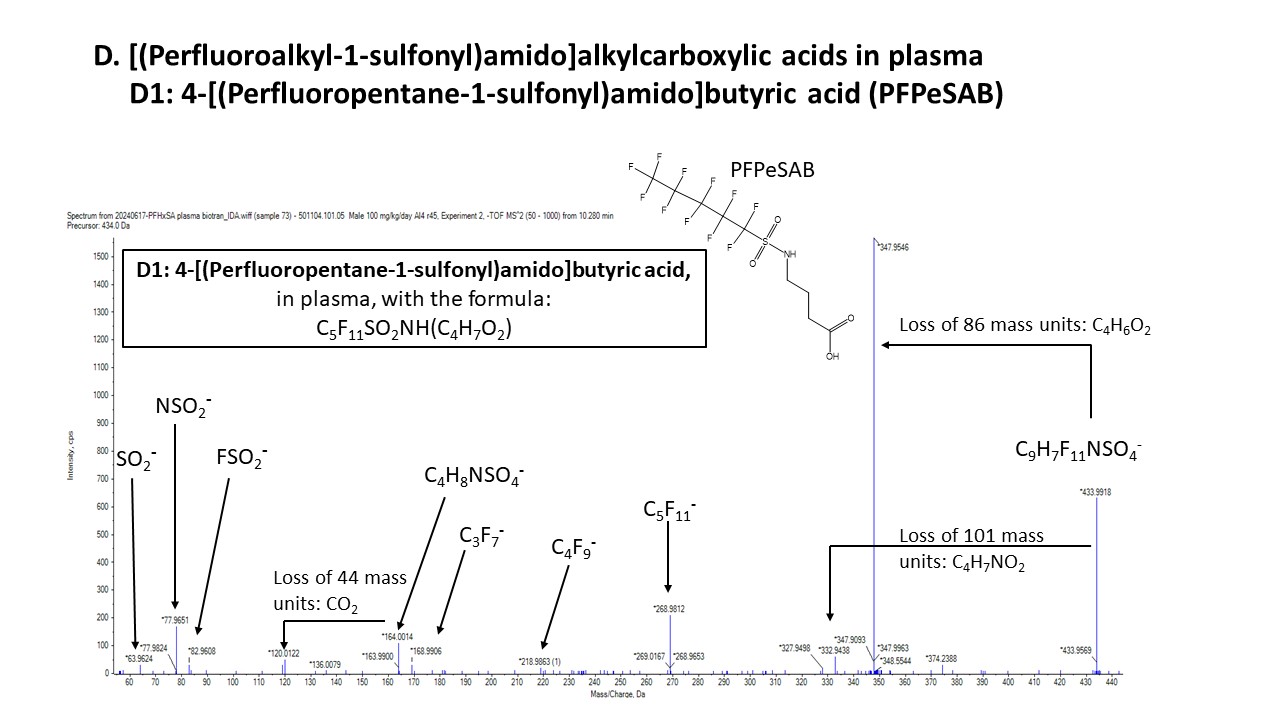

Supplement: Supplementary file 1 [file toxics-13-00523-s001.zip › SI Fig S2 Annotated Spectra/Slide16.JPG]

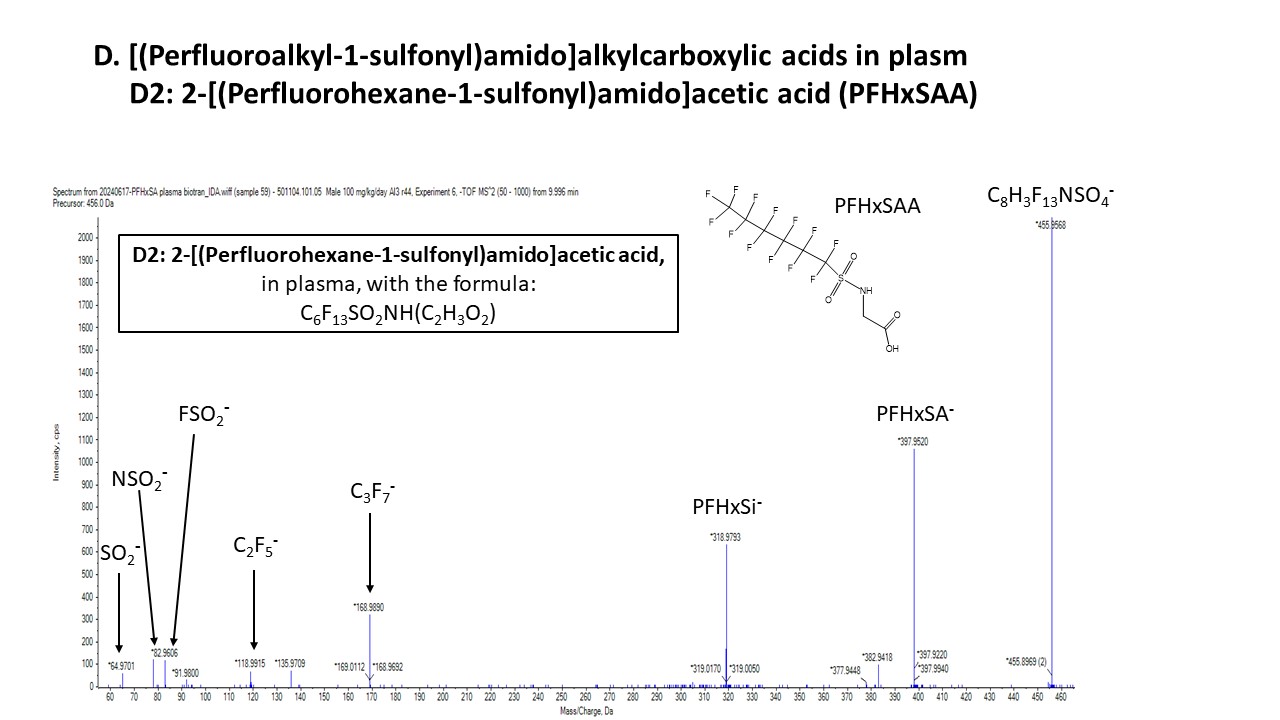

Supplement: Supplementary file 1 [file toxics-13-00523-s001.zip › SI Fig S2 Annotated Spectra/Slide17.JPG]

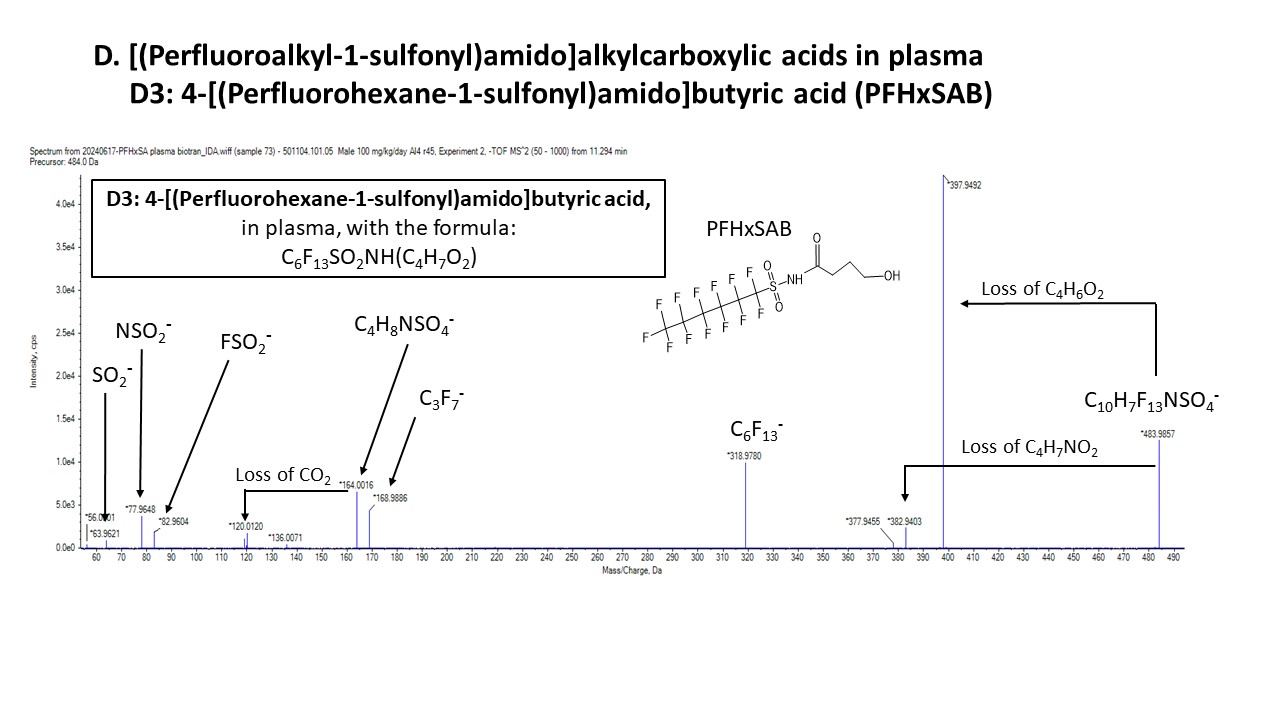

Supplement: Supplementary file 1 [file toxics-13-00523-s001.zip › SI Fig S2 Annotated Spectra/Slide18.JPG]

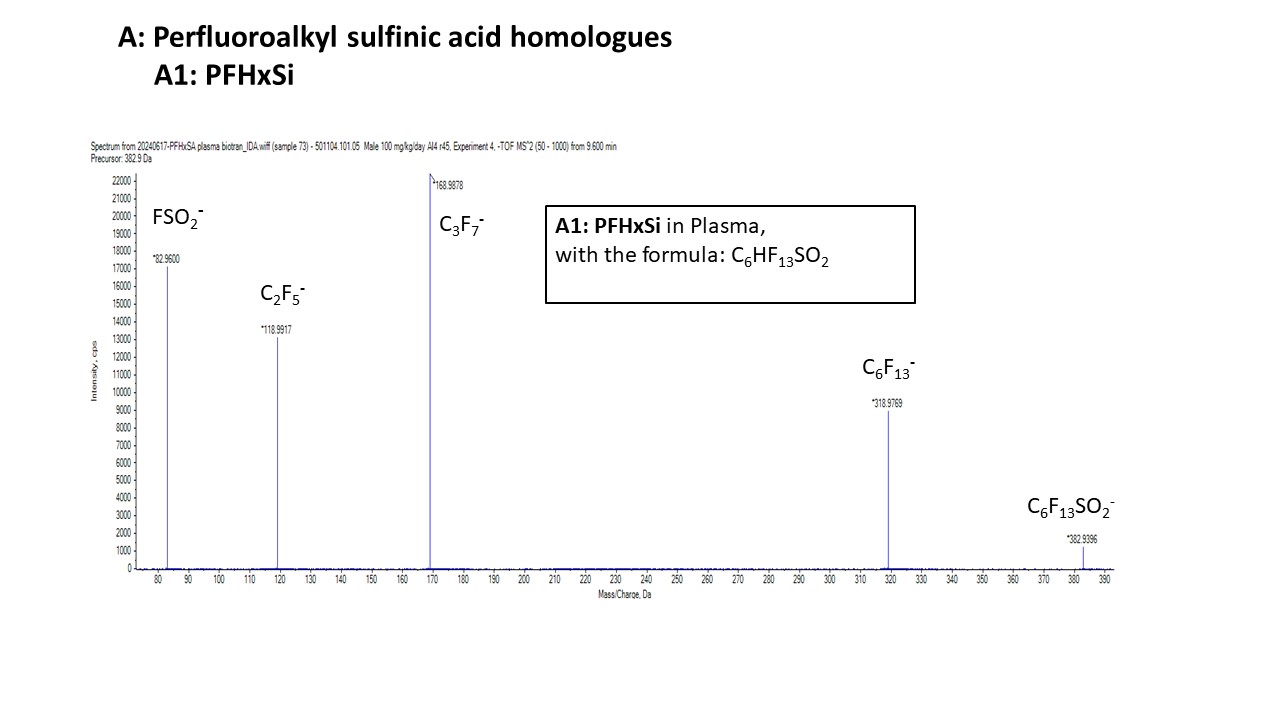

Supplement: Supplementary file 1 [file toxics-13-00523-s001.zip › SI Fig S2 Annotated Spectra/Slide2.JPG]

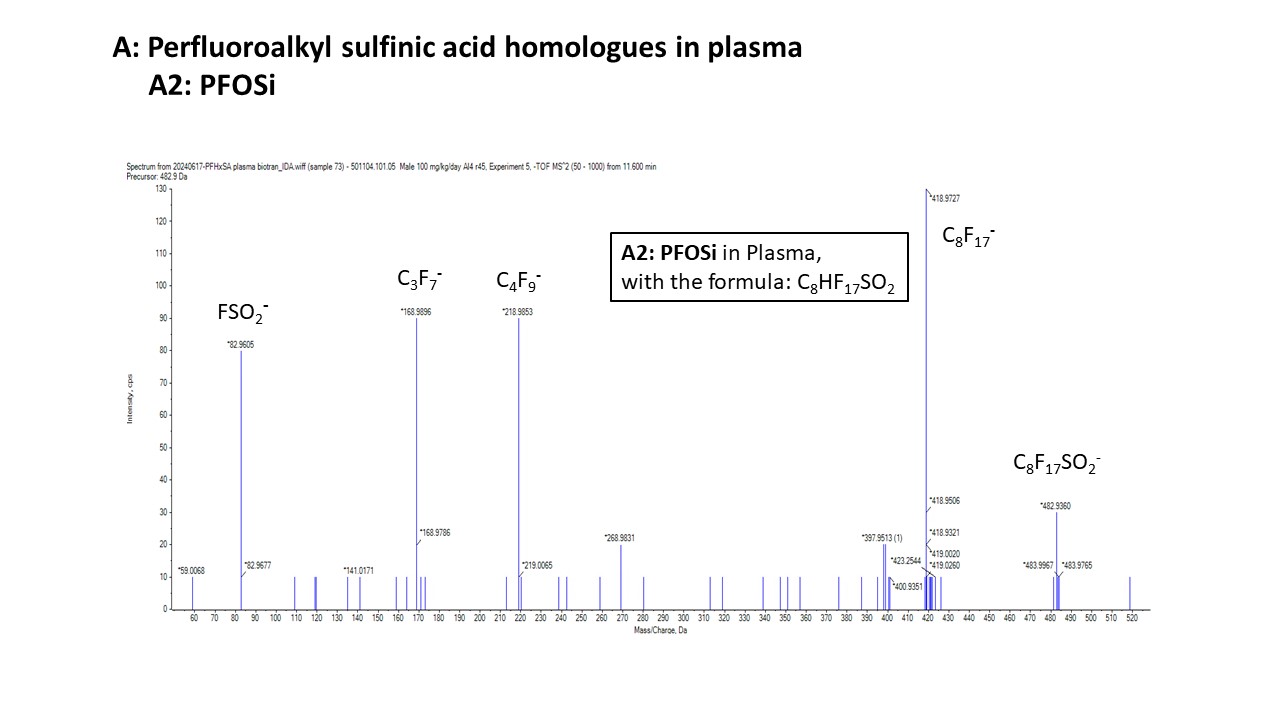

Supplement: Supplementary file 1 [file toxics-13-00523-s001.zip › SI Fig S2 Annotated Spectra/Slide3.JPG]

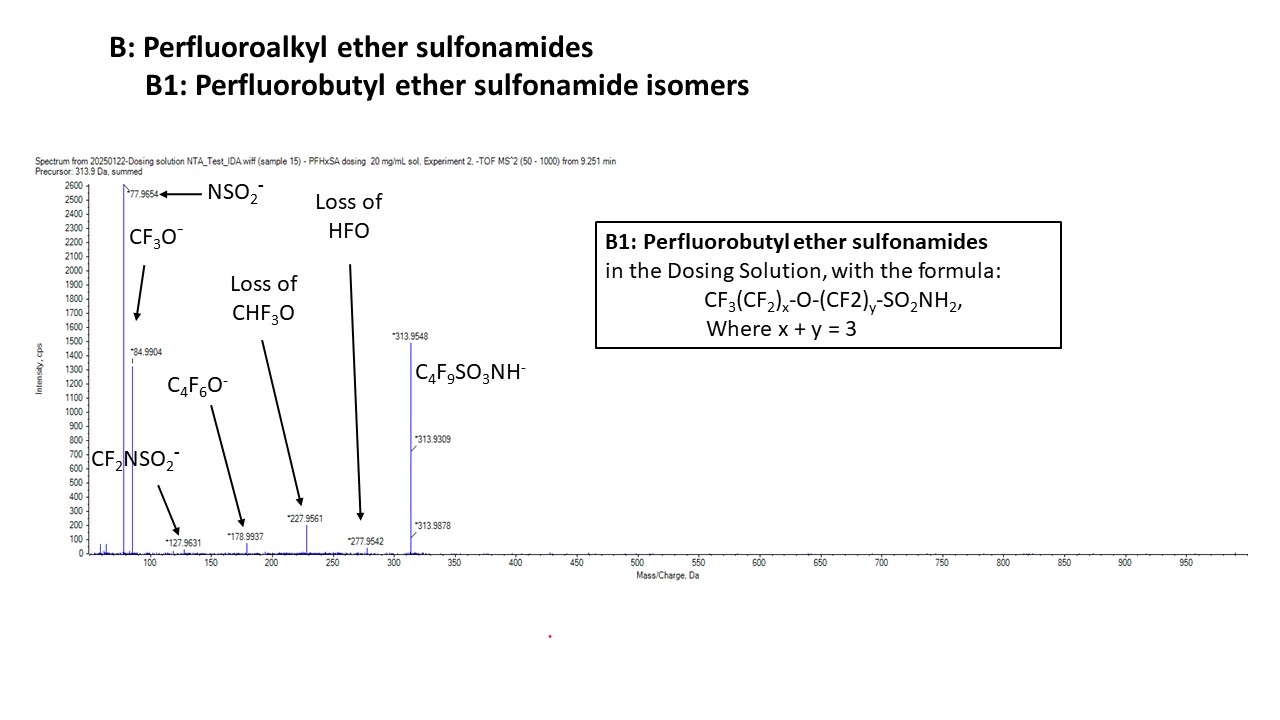

Supplement: Supplementary file 1 [file toxics-13-00523-s001.zip › SI Fig S2 Annotated Spectra/Slide4.JPG]

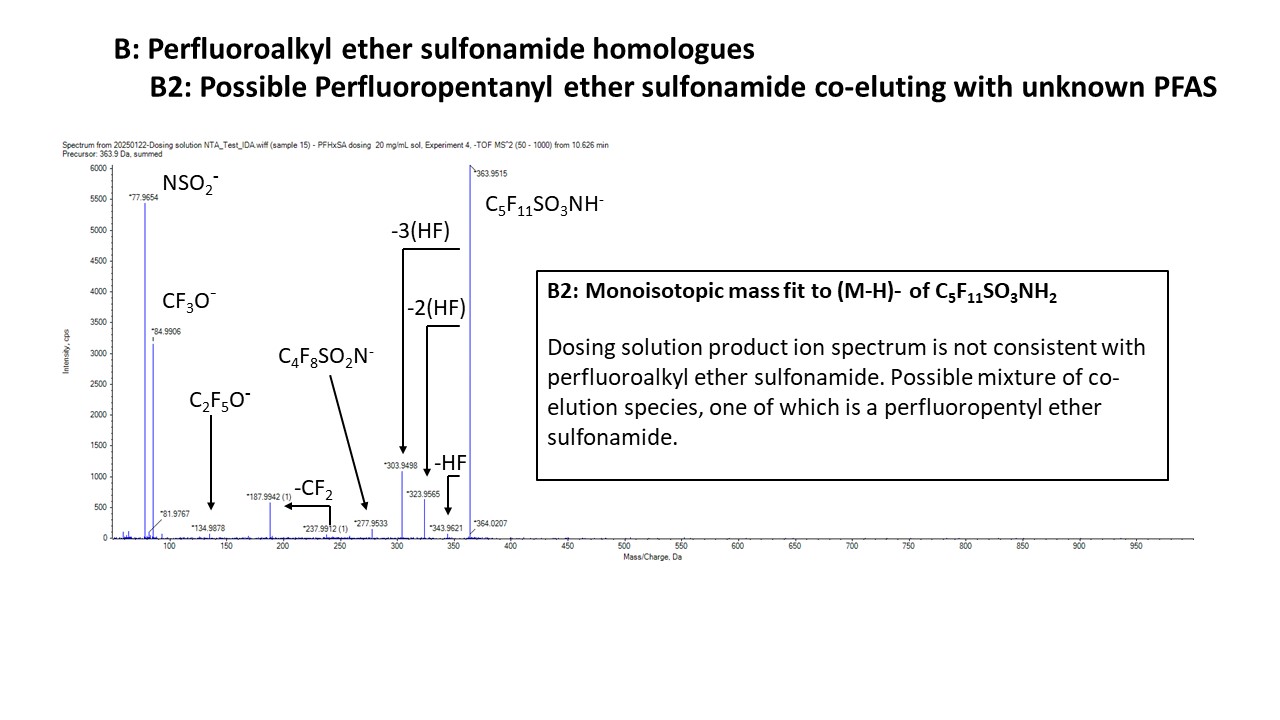

Supplement: Supplementary file 1 [file toxics-13-00523-s001.zip › SI Fig S2 Annotated Spectra/Slide5.JPG]

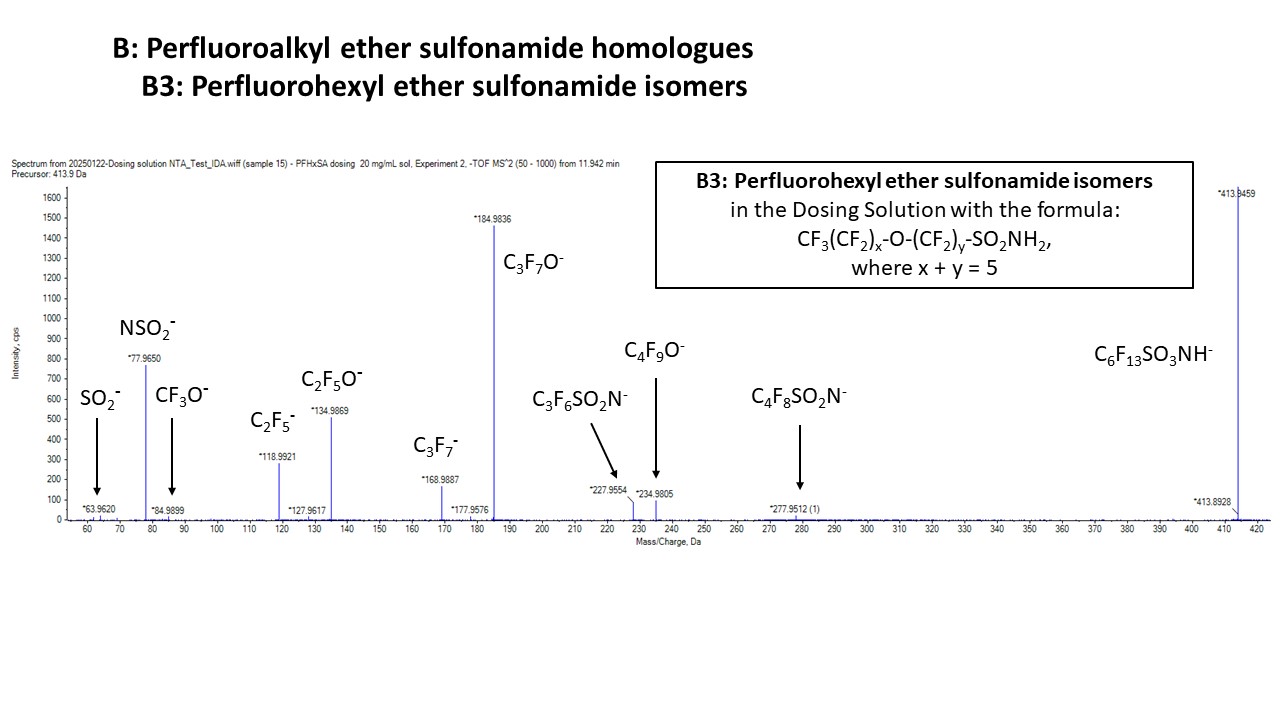

Supplement: Supplementary file 1 [file toxics-13-00523-s001.zip › SI Fig S2 Annotated Spectra/Slide6.JPG]

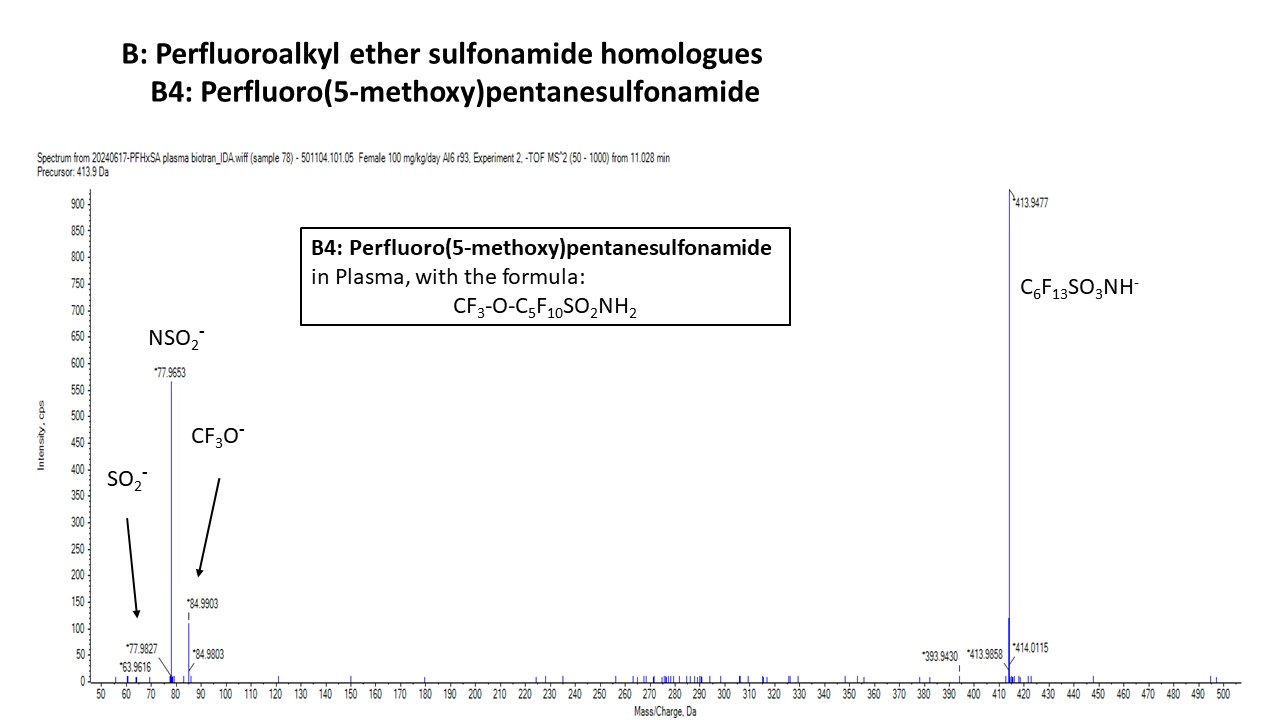

Supplement: Supplementary file 1 [file toxics-13-00523-s001.zip › SI Fig S2 Annotated Spectra/Slide7.JPG]

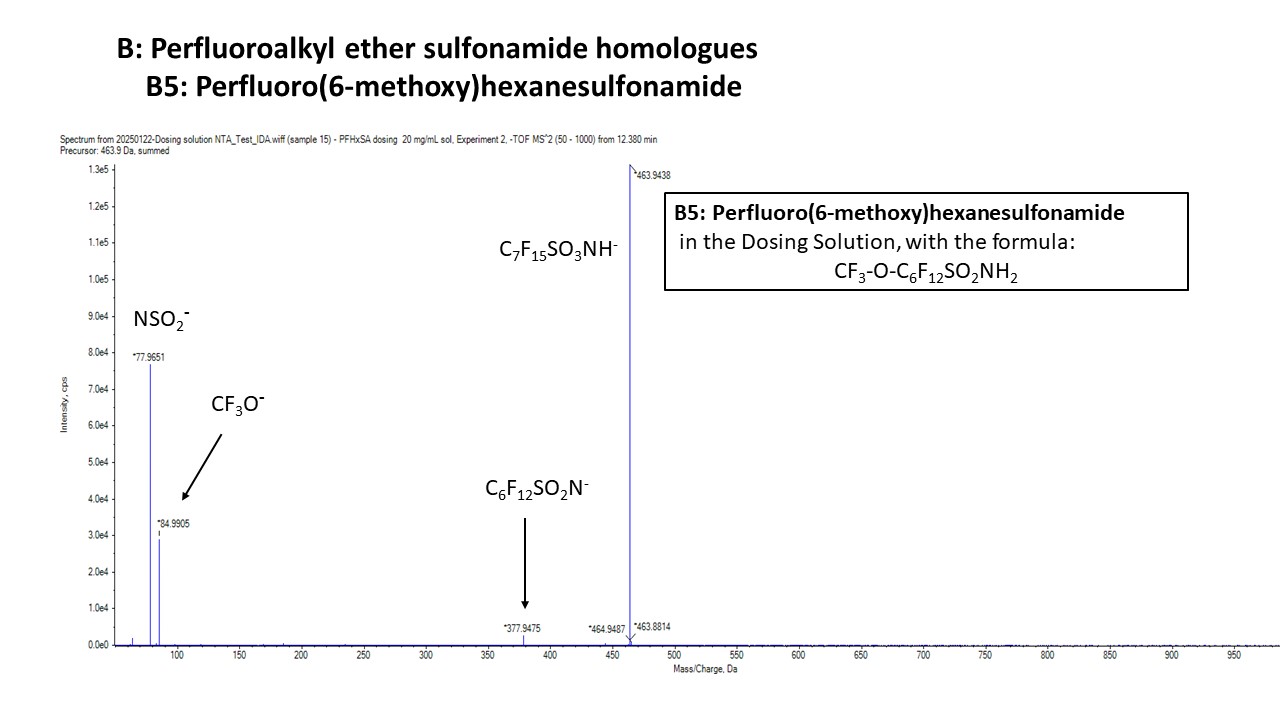

Supplement: Supplementary file 1 [file toxics-13-00523-s001.zip › SI Fig S2 Annotated Spectra/Slide8.JPG]

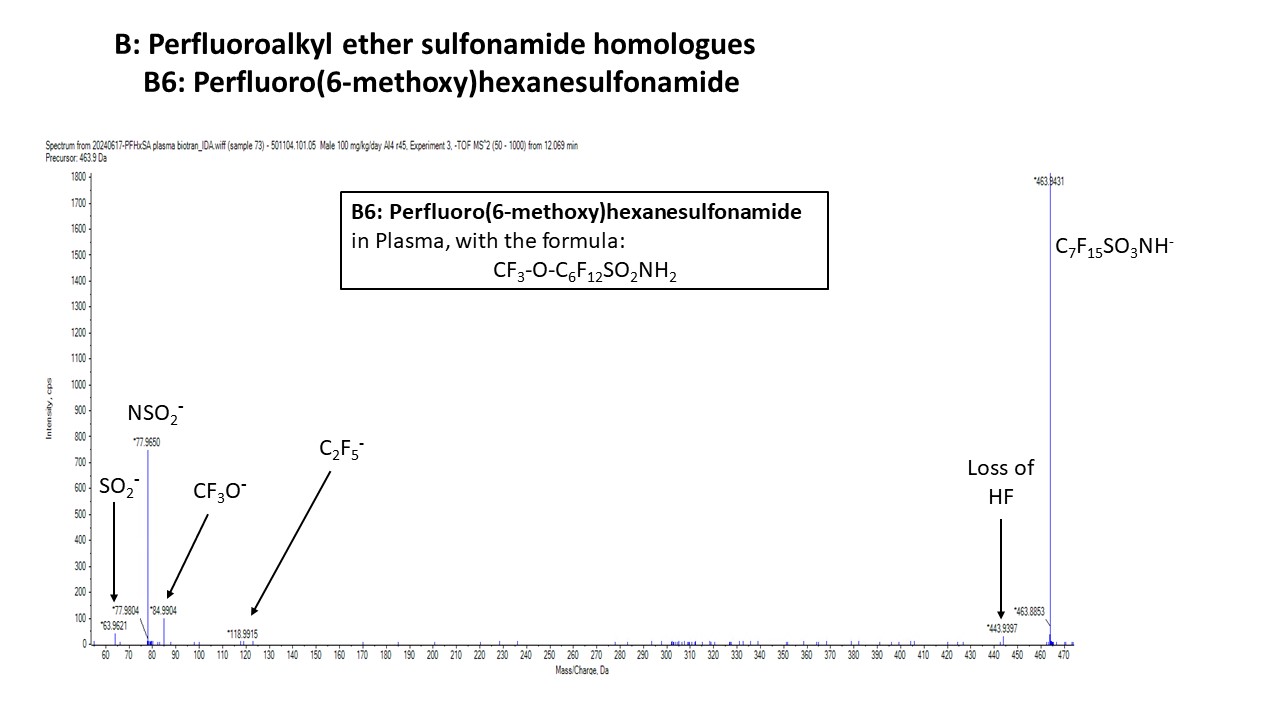

Supplement: Supplementary file 1 [file toxics-13-00523-s001.zip › SI Fig S2 Annotated Spectra/Slide9.JPG]

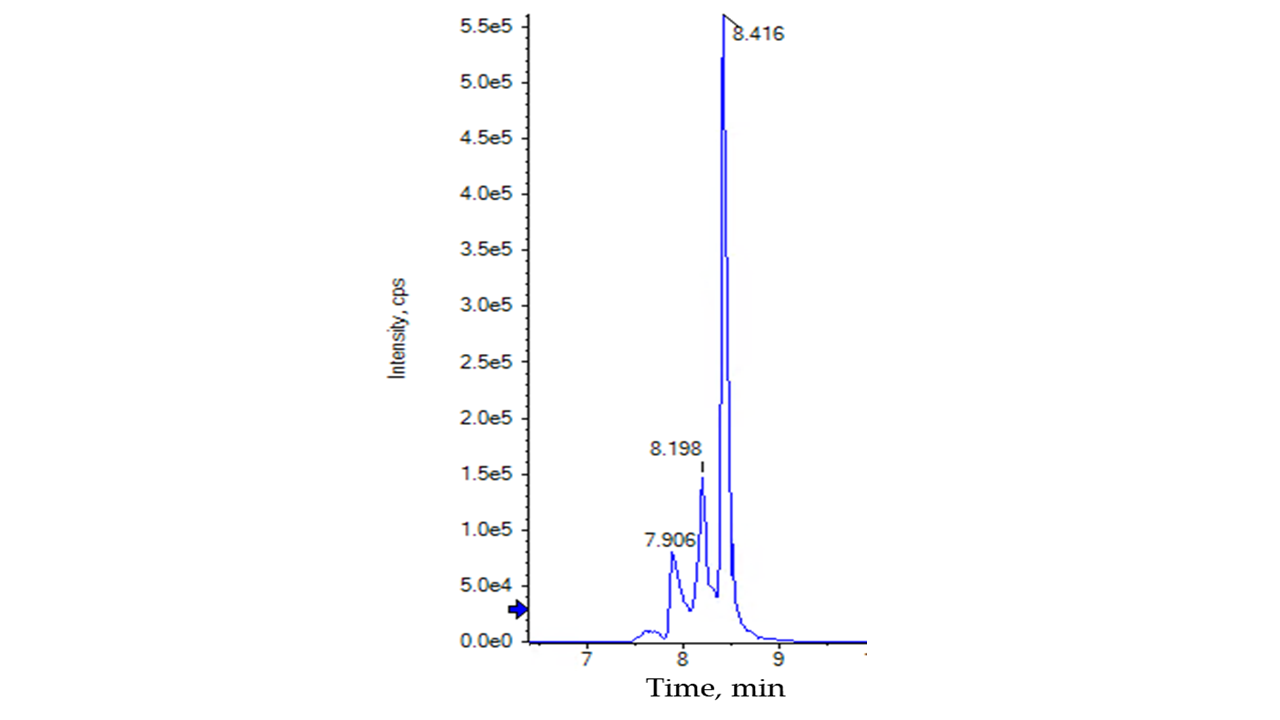

Supplement: Supplementary file 1 [file toxics-13-00523-s001.zip › SI Figure S3 PFHxSA-N-glucuronide chromatogram.png]

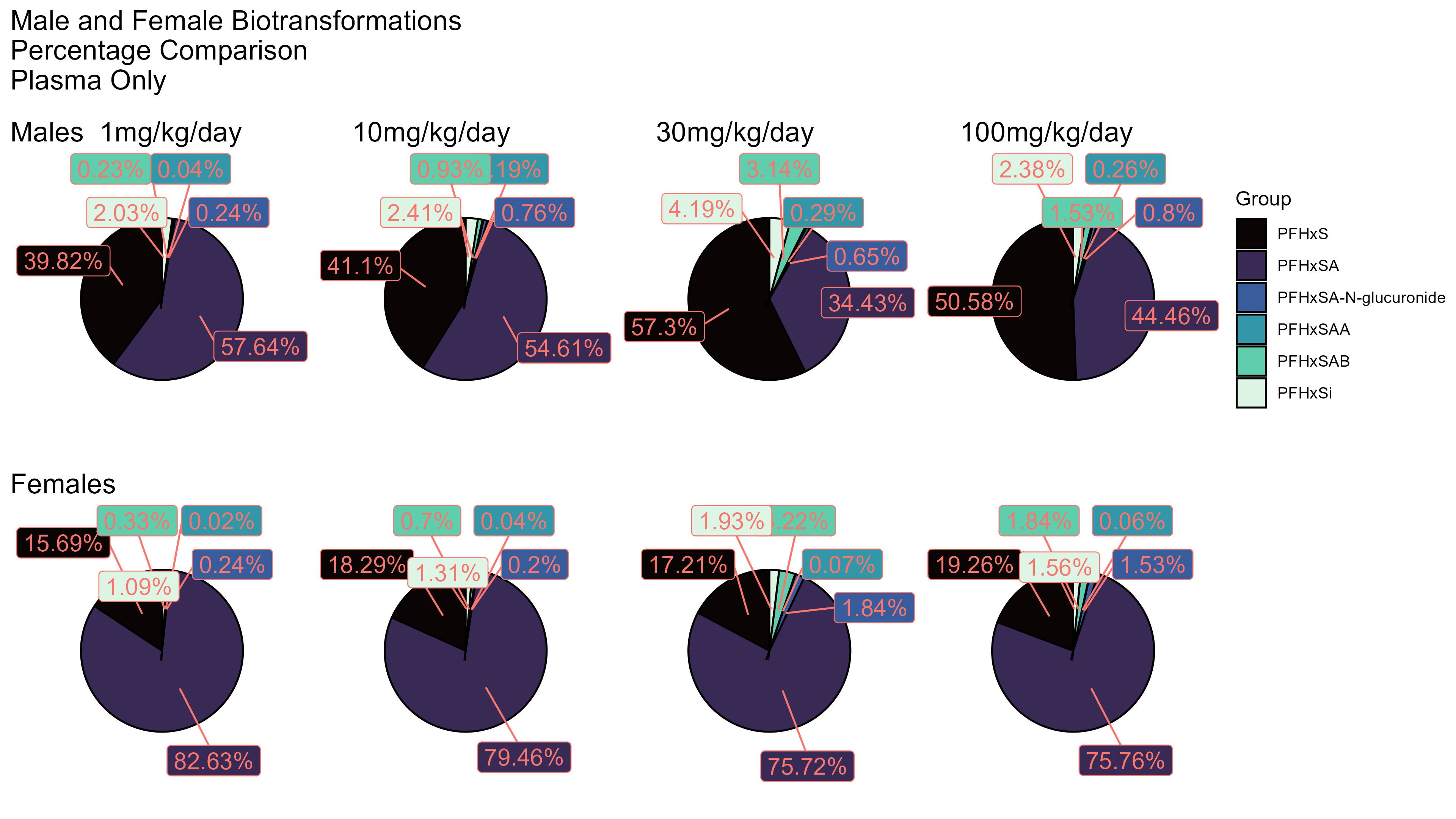

Supplement: Supplementary file 1 [file toxics-13-00523-s001.zip › Si Figure S5 6 Co-occuring PFAS.png]
